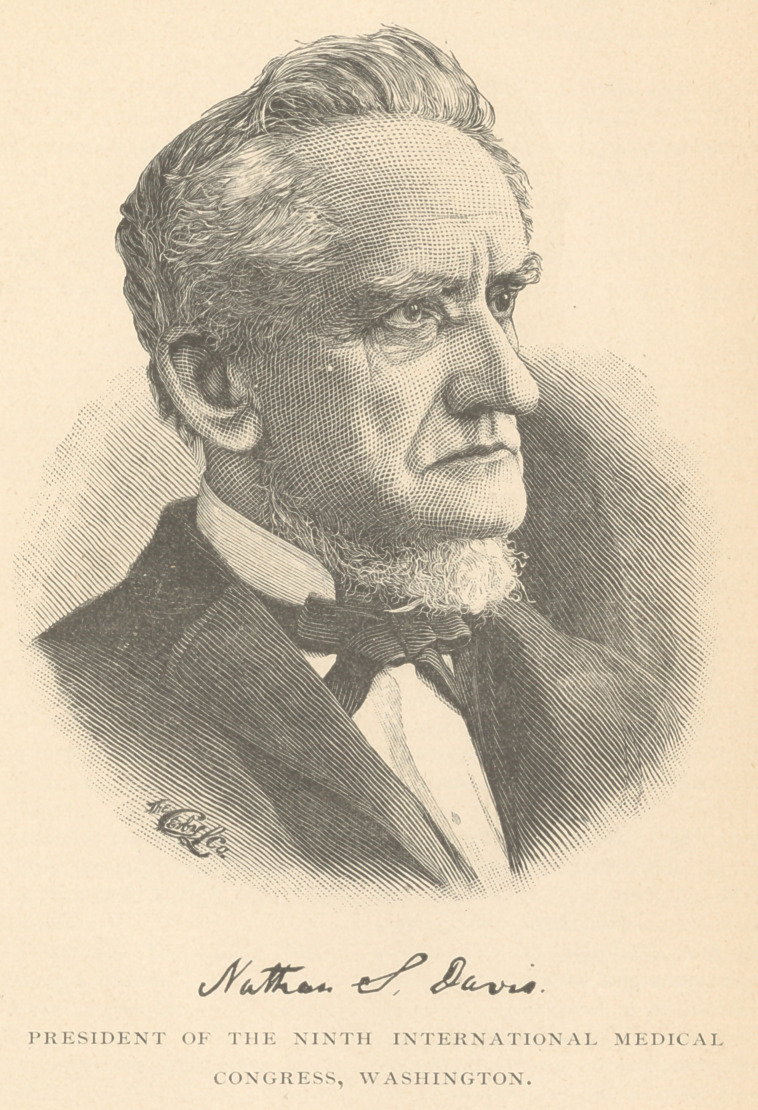# Ninth International Medical Congress

**Published:** 1887-09

**Authors:** 


### NINTH INTERNATIONAL MEDICAL CONGRESS.

HELD IN WASHINGTON, D. C., SEPTEMBER 5, 6, 7, 8, 9, AND 10, 1887.

[Abstracted chiefly from advance slips supplied by *The Medical Record*, of New York, from its Special Report.]

### FIRST DAY.

MONDAY, SEPTEMBER 5TH.

The Congress assembled in Albaugh's Opera House, and was formally opened at 11 A.M. by His Excellency Grover Cleveland, President of the United States, who said: "I feel that the country should be congratulated to-day upon the presence at our capital of so many of our own citizens, and those representing foreign countries who have distinguished themselves in the science of medicine, and are devoted to its further progress. My duty in this connection is a very pleasant and a very brief one. It is simply to declare that the Ninth International Medical Congress is now open for organization and for the transaction of business."

Dr. Henry H. Smith, of Philadelphia, Chairman of the Executive Committee, next named the following

OFFICERS OF THE CONGRESS.

PRESIDENT — Nathan Smith Davis, M.D., LL.D., Chicago, Illinois.

VICE-PRESIDENTS—J.McCall Anderson,M.D., F.R.C.S., Glasgow, Scotland; Thomas Annandale, M.D.,F.R.C.S. (Edinburgh), M.R.C.S. (England), Edinburgh, Scotland; Docteur Dujardin-Beaumetz, Paris, France; Cuthbert Hilton Golding-Bird, M.D., F.R.C.S.,London, England; Professor Carl Braun, M.D., Vienna, Austria; William Brodie,M.D.,Detroit, Michigan; Jno. Chiene, M.D., F.R.C.S., Edinburgh, Scotland; George Joseph Hamilton Evatt, M.D., L.R.C.S., East Indies; Sir B. Walter Foster, M.D., F.R.C.P., Birmingham, England; Ernest Hart, M.R.C.S., London, England; Johnathan Hutchinson, F.R. C.S., London, England; George Murray Humphrey, M.D., F.R.C.S., Cambridge, England; Sir Thomas Longmore, C.B., F.R.C.S., Netley, England; Frederick B. Jessett, F.R.C.S., London, England; Sir William Gull, M.D., LL.D. (Cantab.), F.R.S., D.C.L. (Oxon.), London, England; William Wirt Dawson, M.D., Cincinnati, Ohio; Thomas Michael Dolan, M.D., F.R.C.S., Halifax, England; Thomas Richard Fraser, M.D., F.R.S.,Edinburgh, Scotland; Sir James A. Grant, M.D., Ottawa, Canada; James Andrew S. Grant, M.D., LL.D., Bey, Cairo, Egypt; A.L. Gusserow, M.D., Berlin,Prussia; Dr.Hans Ritter von Hebra, Vienna, Austria; Thomas John Maclagan, M.D., M.R.C.P., London, England; Sir Douglas Maclagan, M.D , F.R.C.P., Edinburgh, Scotland; Withers Moore, M.D., F.R.C.P. (London), M.R.C.S., Brighton, England; John Marshal, F.R.C.S., London, England; Morell Mackenzie, M.D., M.R.C.S., London, England; Sir Henry Thompson, F.R.C.S., London, England; Sir William Roberts, M.D., F.R.C.P., (London), F.R.S., Manchester, England; George B. Macleod, M.D., F.R.S., Glasgow, Scotland; John S. McGrew, M.D., Honolulu, S.I.; Edward M. Moore, M.D , LL.D., Rochester, New York; Karl von Mosengeil, M.D., Bonn, Germany; Professor W. D. Müller, Berlin, Germany; W. Murrell, M.D., M.R.C.S., London, England; C. D. F. Phillips, M.D., F.R.C.S., London, England; Richard Quain, F.R.C.S., London, England; Tobias G. Richardson, M.D., New Orleans, Louisiana; William H. Savory, F.R.C.S., London, England; Sir John Tomes, F.R.C.S., Surrey, England; Sir John Watt Reid, K.C.B., M.D., LL.D, London, England; Sir William Stokes, M.D., F.R.C.S., Dublin, Ireland; Lawson Tait, M.D., F.R.C.S., Birmingham, England; John Burdon Sanderson, M.D., LL.D., F.R.C.P., Oxford, England; Lewis A. Sayre, M.D., New York, N. Y.; Professor Mariano Semmola, Naples, Italy; Dr. Leopold Servais, Antwerp, Belgium; Sir William Turner, M.B.,F.R.C.S.,Edinburgh, Scotland; Dr. P. G. Unna, Hamburgh, Germany; Dr. J. E. de Virji, Hague; Professor F. Winckel, M.D., Munich, Bavaria; A. Y. P. Garnett, M. D., Washington, D. C.; John Moore, M.D., Surgeon-General United States Army; Francis P. Gunnell, M.D., Surgeon-General United States Navy; Sir Edward H. Sieveking, M.D., LL.D., F.R.C.P., London, England; William Harris Lloyd, M.D., L.R.C.S., London, England; Dr. Nicholas José Guiteras, Havana, Cuba; Joseph Ewart, M.D., F.R.C.P., Brighton, England; Professor Friedrich Esmarch, Kiel, Germany; John Tweedy, M.D., F.R.C.S., London England; Sir William Jenner, M.D., K.C.B., D.C.L. (Oxon.), London, England; Dr. F. Dumont, Berne, Switzerland; Dr. A. Pearce Gould, London, England; Dr. Waldeyer, Berlin,

FIRST PRESIDENT-ELECT OF THE NINTH INTERNATIONAL MEDICAL CONGRESS, WASHINGTON.

Nathan S. Davis.

PRESIDENT OF THE NINTH INTERNATIONAL MEDICAL CONGRESS, WASHINGTON.

Germany; Dr. O. Morisani, Naples, Italy; Dr. Wilhelm Meyer, Copenhagen, Denmark; Jeffrey A. Marston, M.D., M.R.C.S., London, England; Joseph R. Smith, M.D., New York, N.Y.; John Dennis Macdonald, M.D., M.R.C.S., Surrey, England; Dr. William Murrell, London, England; Dr. John Marshall, London, England; Professor C. F. Durante, Rome, Italy; Dr. Theodore Kocher, Berne, Switzerland; Dr. John Tweedy, London, England; Professor Trelat, Paris, France; Dr. J. M. Toner, Washington, D. C.; Dr. Charpentier, Paris, France; Dr. Chervin, Paris, France; Dr. Léon Sable, Paris, France; Dr. Léon Le Fort, Paris, France; Dr. Vallin, Paris, France; Dr. Neudörfer, Vienna, Austria; Dr. Von Coler, Berlin, Prussia; Sir James Arthur Hanbury, M.B., K.C.B., F.R.C.S., London, England; William Alexander Mackinnon, C.B., L.R.C.S., London, England.

SECRETARY GENERAL.—John B. Hamilton, M.D., Washington, D.C.

TREASURER.—E. S. F. Arnold, M.D., M.R.C.S., Newport, Rhode Island.

Chairman of the Finance Committee.—Richard J. Dunglison, M.D., Philadelphia, Pennsylvania.

Chairman of the Executive Committee.—Henry H. Smith, M.D., LL.D., Philadelphia, Pennsylvania.

Chairman of the Committee of Arrangements.—A. Y. P. Garnett, M.D., Washington, D. C.

PRESIDENTS OF SECTIONS.

Section I. General Medicine—Abraham B. Arnold, M.D., Baltimore, Maryland.

Section II. General Surgery—William T. Briggs, M.D., Nashville, Tennessee.

Section III. Millitary and Naval Surgery and Medicine—Henry H. Smith, M.D., LL.D., Philadelphia, Pennsylvania.

Section IV. Obstetrics—De Laskie Miller, M.D., Ph. D., Chicago, Illinois.

Section V. Gynecology—Henry O. Marcy, M.D., Boston, Massachusetts.

Section VI. Therapeutics and Materia Medica—Traill Green, M.D., LL.D., Easton, Pennsylvania.

Section VII.—Anatomy—William H. Pancoast, M.D., Philadelphia, Pennsylvania.

Section VIII. Physiology—John H. Callender, M.D., Nashville, Tennessee.

Section IX. Pathology—Alonzo B. Palmer, M.D., LL.D., Ann Arbor, Michigan.

Section X. Diseases of Children—J. Lewis Smith, M.D., New York, New York.

Section XI. Ophthalmology—Julian J. Chisolm, M.D., Baltimore, Maryland.

Section XII. Otology—Samuel J. Jones, M.D., LL.D., Chicago, Illinois.

Section XIII. Laryngology—William H. Daly, M.D., Pittsburg, Pennsylvania.

Section XIV. Dermatology and Syphilography—Andrew R. Robinson, M.D., New York, New York.

Section XV. Public and International Hygiene—Joseph Jones, M.D., New Orleans, Louisiana.

Section XVI. Climatology and Demography—Albert L. Gihon, M.D., U. S. Navy.

Section XVII. Psychological Medicine and Nervous Diseases—Judson B. Andrews, M.D., Buffalo, New York.

Section XVIII. Dental and Oral Surgery—Johathan Taft, M.D., Cincinnati, Ohio.

REPORT OF THE SECRETARY-GENERAL.

Mr. President: According to the precedent set at former sessions of this body, the Secretary-General must make a report of the work performed since the session last preceding, but I will only occupy the time of the Congress for the briefest possible space.

It is now a matter of history that in May, 1884, the American Medical Association met in this capital, and passed a resolution inviting the Congress to honor America by holding its next session in the United States.

At the meeting in Copenhagen, in August, 1884, when the question came up for disposition, Washington was selected. The committee, having borne the invitation and secured its acceptance, returned home, and immediately began the work of organization, and shortly before the meeting of the American Medical Association in New Orleans, in May, 1885, they completed the preliminary organization. But it transpired that this committee were unable to frame an organization satisfactory to the majority of the members of the Association, and, after some discussion, a resolution was adopted which authorized the appointment of additional members of the committee, and, in accordance with our American system of representation, the committee consisted of one member from each State and Territory of the Union, to which was added one representative from each of the three public medical services, and these new members were elected by the State and Territorial delegations. The enlarged committee met in Chicago a few weeks after the New Orleans meeting of the American Medical Association, and several of the members of the first committee were present and acted harmoniously with the committee. In a short time, however, each of the original comn 'ttee had withdrawn, and the management was thus deprived of their experienced and valued services. The committee have, therefore, had to contend against more than the ordinary difficulties attending so great an undertaking, and its present success is due entirely to the zeal and energy of its chairman, Professor H. H. Smith, of Philadelphia, and the unflagging interest and industry of the remaining members of the committee.

Dr. A. Y. P. Garnett, of Washington, D. C., Chairman of the Local Committee of Arrangements, then announced the arrangements for the social entertainment of the members of the Congress and their families.

THE ADDRESS OF WELCOME

was delivered by Hon. Thomas F. Bayard, Secretary of State. In the name of his fellow-countrymen he expressed gratification at the visit of the delegates to Washington. The world is becoming acquainted and international intimacy is growing; a spirit of common brotherhood is increasing so that the word "stranger" will soon be obliterated from the vocabulary of civilization. If letters constitute a republic, science is a democracy. In the United States individual enterprise has produced great scientific institutions without the aid or interference of government. The proceedings of the Congress will be watched with interest by the sixty million people of this country.

RESPONSES

were made by the follwing gentlemen:

Dr. William H. Lloyd, of the Royal Navy; Dr. Léon Le Fort, of France; Dr. P. G. Unna, of Germany; Professor Şemmola, of Italy; Dr. Charles Reyher, of Russia.

Professor Lewis A. Sayre, of New York, occupied the chair during the delivery of

THE ADDRESS OF THE PRESIDENT OF THE CONGRESS.

Professor Davis began by paying an eloquent tribute to the memory of Austin Flint, M.D., LL.D., the first President-elect, and continued as follows:

With a full consciousness of my own deficiencies, and still with a heart overflowing with gratitude, I thank you for the honor you have bestowed in selecting me to preside over the deliberations of this great and learned assembly. It is an honor that I appreciate as second to no other of a temporal nature, because it has been bestowed neither by conquest nor hereditary influence, nor yet by partisan strife, but by the free expression of your own choice.

The living human body, the chief object of your solicitude, not only combines in itself the greatest number of elementary substances and the most numerous organs and varied functions, so attuned to harmonious action as to illustrate the operation of every law of physics, every known force in nature, and every step in the development of living matter, from the simple aggregation of protoplasm constituting the germinal cell to the full-grown man, but it is placed in appreciable and important relations with the material objects and immaterial forces existing in the world in which he lives.

Hence a complete study of the living man, in health and disease, involves a thorough study, not only of his structure and functions, but more or less of every element and force entering into the earth, the air, and the water, with which he stands in constant relation.

The medical science of to-day, therefore, embraces not only a knowledge of the living man, but also of such facts, principles, and materials gathered from every other department of human knowledge as may increase your resources for preventing or alleviating his suffering and of prolonging his life.

The time has been when medical studies embraced little less than the fanciful theories and arbitrary dogmas of a few leading minds, each of whom became for the time the founder of a sect or so-called school of medicine, with his disciples more or less numerous. But with the development of general and analytical chemistry, of the several departments of natural science, of a more practical knowledge of physics, and the adoption of inductive processes of reasoning, the age of theoretical dogmas and of medical sects blindly following some more plausible leader passed away, leaving but an *infinitesimal* shadow yet visible on the medical horizon.

The address closed with an appeal for the collective investigation of the phenomena of disease.

The Congress then adjourned.

---

### SECTION ON ANATOMY.

William H. Pancoast, M. D., Philadelphia, Pennsylvania, President.

Secretaries.—Dr. John J. Berry, Portsmouth, New Hampshire; Dr. William C. Wile, Danbury, Connecticut; Dr. C. Remy, Paris, France.

FIRST DAY—AFTERNOON SESSION.

The president's address was postponed to the second day.

The first paper read was by J. M. Matthews, M.D., of Louisville, Kentucky, on

THE ANATOMY OF THE RECTUM AND ITS RELATION TO REFLEXES.

He began by reviewing the anatomical bearings of these parts, which he thought had been much neglected. He thought that the reflexes had not been sufficiently considered, and spoke of the difficulty with which these parts were brought under the influence of an anæsthetic on account of their extensive nerve supply. He spoke of the many causes of constipation causing congestion of the mucous membrane and reflex spasm of the sphincter muscle. He related cases showing the large number and variety of symptoms rectal irritation could produce, and particularly a case of obstinate constipation simulating locomotor ataxia. He also dwelt upon the importance of examining the rectum in certain cases of constipation, and of exploring it high up.

Dr. Gervais, of Belgium, said he had seen many cases of reflex irritation from the rectum. He asked if injections of carbolic acid were practiced both for external and internal hæmorrhoids.

Professor F. C. Schaefer, of Chicago, Illinois, reported having seen cases in which most excruciating pain through the perineum had been removed by simply relieving the constipation, showing the reflex character of pain.

Dr. William C. Wile, of Danbury, Connecticut, related a case of hæmorrhoids from which the patient had become insane. The insanity was cured shortly after removing the hæmorrhoids.

Dr. Albert B. Strong, of Chicago, Illinois, mentioned a very severe case of gonorrhœa in his practice which was only cured after some existing piles had been removed.

Dr. Gallet, of Belgium, had witnessed cases similar to those related by Dr. Matthews.

Dr. J. M. Matthews, of Louisville, Ky., further stated that he did not spare the knife in operations for the removal of hæmorrhoids, but preferred it to all other means in its proper place. He spoke of the dangers attending the injection of carbolic acid. He laid great stress upon the point that pruritus ani should not be treated as a disease, but as a symptom, the cause of which must first be removed to effect a cure.

The President mentioned an instrument which he had used for some time for the treatment of rectal stricture. This instrument, which he had not yet exhibited before any society, consisted of a rectal sound formed on anatomical principles to conform to the curved direction of the rectum. He dwelt upon the importance of the proper curves in this instrument.

Dr. Ambrose L. Ranney, of New York, N.Y., then read a paper entitled,

DOES A RELATIONSHIP EXIST BETWEEN ANOMALIES OF THE VISUAL APPARATUS AND THE SO-CALLED NEUROPATHIC TENDENCY?

He thought that the neuropathic tendency an important factor as a cause of the largest mortality. He was of the opinion that many neuroses were caused by an eye defect. He quoted from the statistics collected by Dr. George Stevens, of New York, and from his own carefully studied one hundred cases. His deductions were as follows:

1. No one has yet shown in what this predisposition lies; hence it can be shown that eye defect is an important element in these conditions.

2. There is no recognized pathology in functional nervous diseases. The view that they are constitutional diseases is by no means established by pathological research.

3. Heredity is very common in these affections. It is one of the most marked features in this class of nervous diseases.

4. My records go to show that eye defects are found in a very large proportion of such subjects.

5. Many of the eye defects found can be shown to be congenital, being inherited like other peculiarities.

6. The manifestations of the neuropathic predisposition vary with each case. They are called forth often by extremely trivial causes. These accidental circumstances or simple coincidences are too frequently recorded as of great clinical interest.

Dr. Ranney tested all eyes with a four per cent. solution of atropine. He quoted many cases, principally of epilepsy and melancholia, which had been under the care of the best neurologists, and which had been cured by correcting errors of vision. He believed that there was absolutely no cases of migraine without eye defect. He said he could cure seventy-five per cent. of such cases.

Dr. Pierce, of Ohio, cited cases of chorea, caused by fright and cases in which the chorea had been cured and in which squint continued.

Dr. William C. Wile, of Danbury, Connecticut, then read a paper entitled

WHICH SHALL BE THE SITE OF A URINARY FISTULA?

in which he appealed to the Section to decide the question. He cited a case in which a very hypertrophied prostate gland, made the operation of entering the bladder exceedingly difficult.

The president said he was not at all afraid of the prostate gland, but treated it as a foreign body when operating.

The section then adjourned.

---

### SECTION ON DERMATOLOGY AND SYPHILOGRAPHY.

A. R. Robinson, M.D., New York, N.Y., President.

Secretary.—Dr. William S. Gottheil, M.D. New York, N.Y.

First Day—Afternoon Session.

Professor Robinson, the president, opened the Section with a few general remarks, and then read his opening address. He called attention to the inadequacy of the course of study in our American medical schools, necessitating a trip to Europe to perfect one's self in dermatology and other specialties. He regarded it as an error to combine in our medical schools the study of dermatology with genito-urinary diseases, rather than with general internal pathology, which holds such a close etiological relationship to many cutaneous diseases. The knowledge of dermatology obtained in other languages is apt to influence the student in his future course of study and in his practice. That there are no American views of dermatology and no American school is to be greatly regretted. It is not possible to learn dermatology in this country except under great disadvantages. He called attention to the serious evil of indiscriminate medical writing and compilation to the neglect of original work. The proportion of papers representing original research being noticeably low.

Dr. William Welch, of Philadelphia, Pennsylvania, read a paper on

VACCINATION DURING THE INCUBATION PERIOD OF VARIOLA.

The author endeavored to show that vaccination during the incubation-period of variola has, at least in his own hands, given gratifying results, preventing or modifying the small-pox eruption. The contrary view he believes to be unsupported by facts and not tenable.

Vaccination during the initial stage is valueless. If it be performed very early in the incubation stage it exerts a modifying influence and may prevent the attack altogether.

Vaccinia does not begin to exert its effect until the formation of the areola about the vesicles.

It must not be long delayed after the contagion has been received in the system if favorable results are expected. The character of the vesicle has much to do with the protection secured. When the vesicles are imperfectly formed or retarded the efficacy is to be feared. Animal lymph is too unreliable. Fresh, eighth-day lymph, from a typical vaccine vesicle is to be preferred.

By numerous insertions of the virus we are more sure of good results. He has no doubt the process of vaccinia is hastened by multiple insertions.

He has made observations upon 144 cases, from which he has been led to believe that vaccination in the incubation period gives a certain immunity from variola.

Dr. Cundell Juler, of Cincinnati, Ohio, said he believed that the lymph from the vesicle itself gave an immunity which the crust did not, and when used he did not consider revaccination necessary, but with the crust it was. The lancet should be kept scrupulously clean.

Dr. Rogers Parker, of Liverpool, England, a public vaccinator for the city of Liverpool, said that in the large cities of England human lymph was usually employed. Large vaccination stations were established to which the parents were required to bring the children at certain times, and the children vaccinated the previous week returned upon this day so that there was always an abundance of vesicles from which to choose in vaccinating the others. Revaccination is not compulsory, but was usually done in schools and public bodies at about the age of fourteen.

Dr. Gottheil, of New York, N. Y., said that he had frequently noticed a fungating sore about two weeks after vaccination, which he had regarded as a weak ulcer. With some sets of lymph it always appeared, and with others never.

Dr. Keller, of Hot Springs, Arkansas, related an incident of war times. He was in charge of a hospital on the Southern side. An epidemic of variola broke out. A supply of lymph was received, with instructions that it came from the North, and was suspected of being poisoned. Such stories were then current. He decided to use it, and, to avoid error, divided each point, vaccinating a citizen with one part and a soldier with the other. Nine-tenths of the soldiers had unhealthy sores following the operation, but none of the citizens, showing that they were due to the scorbutic condition of the soldiers, and not to impure virus.

Dr. Yeamans, of Detroit, Michigan, said his belief in the protective power of vaccination, after exposure had occurred, grew yearly less and less. He had had considerable experience in the matter, and thought we lose sight of the many other influences brought to bear which may modify the case under observation.

Dr. Lathrop, of Dover, New Hampshire, believed that he had seen vaccinia take the place of small-pox.

Professor Robinson, the president, asked why, if vaccinia depended on micro-organisms, which multiply so rapidly, more than one point of inoculation was necessary?

Dr. Welch, in closing, said he had been skeptical in regard to the necessity for multiple inoculations, and believed one typical cicatrix gave as good protection as many.

Animal virus, or virus of recent humanization, gives a more durable protection. That of long humanization produces a more superficial scar, and deaths were more frequent in those showing superficial scars. He does not believe in life-long protection of vaccination. The ulcers mentioned by Dr. Gottheil he thought were only found after animal lymph had been used. It is not true vaccination and gives no protection.

RECTAL ALIMENTATION IN DISEASE OF THE SKIN.

Professor J. V. Shoemaker, of Philadelphia, Pennsylvania, said: All substances in solution are speedily absorbed by the rectum. Medicines in the form of suppositories and powders are taken up more slowly, but are cleanly and easy of administration. In lupus of the mouth and lips, after scraping, the rectal method of alimentation and medication is recommended. Pemphigus and impetigo in children of all ages are often due to alimentary disturbance, in which this method has advantages over the administration of food and medicines by the mouth.

Acne due to stomachic irritability, with vomiting, nausea, etc., can be cured in from two to four weeks by giving milk and other nutritious enemata per rectum, care being taken to insure retention. They should be lukewarm, and given only once every four hours.

Rectal medication is of value in those to whom medicines by the mouth are distasteful.

In many cases of syphiloderm the eruption disappears after suppositories of five grains of calomel, or a fraction of a grain of the protiotide.

Scorfuloderma can be cured by rectal enemata of cod-liver oil, when it cannot be taken by the mouth.

Urticaria can at times be best relieved by an enema of an ounce each of castor-oil and glycerine.

Pruritus ani treated by suppository of powdered geranium in ten grain doses acts well.

Arsenic and antimony are of great benefit in some cases of psoriasis, but they may disorder the stomach. They can then be given with care by suppository.

Iodide of potassium in small doses in the form of suppository prevent many disagreeable symptoms produced by this drug.

Dr. P. G. Unna, of Hamburg, Germany, agreed with the reader of the paper in the good effects obtained by rectal medication in many affections. He asked concerning the experience of the author in regard to the administration of mercury. Whether diarrhœas and other unfortunate effects were not likely to occur.

Dr. Klotz, of New York, N.Y., would be afraid that the absorbent power of the rectum would be lowered in certain cases. For example, in pemphigus in children he would be afraid that the method would fail, as in other febrile diseases.

In closing, Professor Shoemaker replied to Dr. Unna's question that when the mercurial was given first in small doses and gradually increased, the good effects were secured and the ill effects obviated.

Dr. H. Klotz, of New York, N.Y., read a paper

ON THE OCCURRENCE OF ULCERS RESULTING FROM SPONTANEOUS GANGRENE OF THE SKIN, DURING THE LATER STAGES OF SYPHILIS, AND THEIR RELATIONS TO SYPHILIS.

Whenever we find a round-shaped, punched-out ulcer, with thickened, abrupt edges involving the entire thickness of the skin, with uneven floor, and a dark-green color, in a person with syphilis in its later stages, we generally consider it a syphilitic ulcer, and most frequently due to the breaking down of gummatous infiltration.

The author's observations have led him to believe that similar ulcers in syphilitics may result directly from circumscribed spontaneous gangrene of the skin. The cases have occurred mostly in dispensary practice, and the histories have not been as accurate as might be desired. In one case in private practice such ulcerations developed upon the leg, along with well-marked ulcers from the breaking down of gummata, and were carefully watched and studied. After the removal of the dry slough, such ulcers exhibit peculiarities which render it possible to recognize their origin. They bear a resemblance to the round ulcer of the stomach. Malignant precocious syphilides may give rise to similar ulcers, and the tuberculo-gangrenous syphilide of Bazin seems to be closely related to them. Syphilis may furnish adequate cause for direct spontaneous gangrene. It has been satisfactorily shown that in every stage of syphilis the virus exerts its action most constantly on and around the blood vessels, and we must look particularly to endarteritis if we can exclude other causes of gangrene. As to cerebral arteritis, its great importance has been sufficiently proved, and is none the less so because it is really not a specifically syphilitic process. That it owes its origin to syphilis in a great many cases is generally conceded. Lang, who has paid particular attention to the influence of syphilis on the vascular system, cites a number of instances. There is really no reason why the skin should be exempt from this process, and particularly if arteritis exists in smaller and terminal arteries. Proof will be furnished only when changes in an artery leading to the gangrenous portion of the skin can be shown to exist. This proof the author has not been able to furnish.

The author concludes :

1. Ulcers resembling the so-called gummatous syphilitic ulcer may occasionally result from circumscribed spontaneous gangrene of the skin, without the formation of a previous syphilitic neoplasm.

2. Such ulcers may be distinguished by several peculiarities of formation of floor and shape.

3. They are but little affected, if at all, by antisyphilitic treatment.

4. Spontaneous gangrene in such cases is probably due to endarteritis obliterans.

In the discussion, Dr. Unna, of Hamburg, said that endarteritis obliterans of the smaller vessels in syphilis is now a well established fact. It is astonishing that in the primary lesions of syphilis we do not have more sloughing than is generally seen.

### SECTION ON GENERAL MEDICINE.

Abram B. Arnold, M.D., Baltimore, Maryland, President.

Secretaries.—J. W. Chambers, M.D., Baltimore, Maryland; William F. Waugh, M.D., Philadelphia, Pennsylvania.

### First Day—Afternoon Session.

After calling the meeting to order, Professor Arnold, the president, read

#### AN ADDRESS IN MEDICINE.

He thought it especially appropriate upon such an occasion to review therapeutics and diagnosis.

In reviewing the therapeutic nihilism at one time prevalent, he contrasted it with that over-confidence placed in drugs which, although similarly irrational, had not been fairly tested.

The treatment of acute febrile diseases were particularly worthy of our study and attention. At present, instead of a uniform there is an English, French, German and American treatment for fevers. It was interesting to note that within the last three or four decades the rate of mortality in fevers had decreased. As illustrating, it was well to consider the therapeutic treatment of typhoid fever, especially in reducing the fever. All drugs which have been used have had the unfortunate action of overwhelming the system in a manner especially worthy of our attention. The evidence of the value of these remedies has not been established. Hyperpyrexia itself is truly a source of great danger. There is no remedy so good as the judicious abstraction of heat from the body. Those who advocate active treatment ought to review the history of the treatment of pneumonia. The superiority of non-interference over active measures allowed this disease to make the fortune of homœopathy.

Inconsiderate medication is due to an erroneous conception of the true nature of the disease. The general principles formulated by the older physicians were on the whole excellent, so far as the general management of their cases was concerned. They treated expectantly, and were ever on the alert for complications. One part absolutely remedial is good nursing and sound hygienic rules. There is a conservative medicine as well as a conservative surgery.

Preventive medicine, which has been fairly organized, constitutes a new epoch in the history of medicine. Bacteriology and vital statistics teach us valuable lessons. The increased attention to hygiene by the people, the amelioration of the poorer classes by removing young children from the factories, and the diminution of the hours of labor, contribute largely to the increase in the longevity of the race.

Pathology has been advancing, yet therapy not equally so. The practitioner classes his diseases in sclerosis, cirrhosis, etc., with far greater ease than he applies his remedies to them. The text-books have dropped the subjects dropsy, paralysis, etc., and are changing clinical into anatomical diseases. Physicians once thought they had specifics, such as calomel and various alteratives. Perhaps we have too completely thrown them aside.

Symptomatic treatment, however, may not be neglected, it is often curative. Structural changes also may be modified by remedies which affect the functions of organs.

The criticisms of experimental therapy are untenable, as by this method we arrive directly at a knowledge of the sphere of action of the remedies. Virchow said, "therapy alone tolerates rubbish," a statement not so true now as when he said it. We ought, therefore, not only to know better the therapeutic action of our remedies, but also to increase the number of these agents by experimentation. The numerical method is imperfect, yet is still the best way to compare different methods.

In closing, he referred to the difficulties of medical practice, and the necessity of earnestness and a keen insight into physiological medicine.

Dr. Ignacio Alvara, of Mexico, read a paper entitled

#### SOME SUGGESTIONS UPON THE PATHOGENESIS OF YELLOW FEVER.

Although previous pathology ascribes the etiology of yellow fever to a certain microbe, the large experience with the disease in Vera Cruz,

Mexico, has led them into the theory that yellow fever is an auto blood-poisoning, either by the acid phosphate of soda of the same blood having been burned from the basic into the acid, or by the phospho-glyceric acid set free from the lecythin by the reactions in both cases that have been produced by the living of the microbes upon the constituents of the sanguineous fluid.

The idea of yellow fever being a case of poisoning by phosphorus is not a new one. Owing to similarity of the symptoms they were referred to phosphorated hydrogen. The two etiological factors in yellow fever are, first: fermentation—the one to which the name yellow fever is fitted, and second, the lesions resulting therefrom. Division of this disease is of course artificial, because many of the stages are at times absent. Having taken the abnormal temperature as the basis for the study of the malady, having noted the symptoms that accompany each one of the three stages of the abnormal heat ascent, fastigium, and descent, and by the facts observed, have arrived at the conclusion that all the cases of yellow fever must be included in two classes: those ending at the fastigium of the abnormal heat, convalescence setting in at once; again, those going on through the descent. Yellow fever has two phases of evolution. The first stage is recovered from, excepting, of course those cases where the stages are indistinct and run together. The theory was advanced that the symptoms of the first stage were caused by an excess of lactic acid in the blood, since poisoning by lactic acid causes aching in the body, dizziness, somnolence, and gastro-intestinal disturbances, identical with those of yellow fever.

Again, it is inferred that the excess of lactic acid, reacting on the remaining constituents of the blood, produces the acid phosphate (or phosphorus-element), which in turn gives symptoms identical with those of the second stage of yellow fever.

He found eighty-eight per centum of his cases had characteristic pains in the muscles of the eyes when they were turned upward. Lumbago was present in ninety-five per cent.

In yellow fever there are two conditions, abnormal heat and excessive pathological changes in the blood. This latter condition needs to be investigated by men competent in that department.

Among methods attempted to cure this disease, Doctor Alvara mentioned intravenous injection of ether, yet without especially favorable results.

Dr. Walter P. Geike, Ontario, Canada, read a paper entitled

PNEUMONIA AS MET WITH IN VARIOUS PARTS OF THE DOMINION OF CANADA.

Pneumonia he had found to be far more frequently secondary than primary; the former was probably most frequently seen as a complication of typhoid fever. In preparing this paper he had corresponded with physicians practicing in newly and sparsely settled countries, and he had found that in these localities, both East and West, it was a rare disease. It never occurred in epidemics. He asks this question: Is it because there are so few inhabitants that there never occurs an epidemic ?

As sanitary methods increase low and asthenic forms and epidemics decrease.

In the recent epidemic in Toronto, the disease seemed to affect both the weak and strong alike. Investigation showed that the disease is more acute in rural, and less so in populated districts. There had been cases in the recent epidemic in which it seemed to be contagious. The specific character of the fever would naturally support such a view. Realizing fully the predisposition to the disease which arises from the abuse of alcoholic stimulants, yet improper drainage and water were the great causes.

There was no uniform treatment.

Dr. J. A. Ochterlony, of Louisville, Kentucky, reviewed some of the different theories now and formerly held in regard to the local or specific origin of this disease.

Dr. W. J. Scott, of Cleveland, Ohio, said that he found it difficult to reconcile the anatomical changes with any of the advanced hypotheses. He believed it to be infectious.

Dr. J. B. Lester, of Kansas City, Missouri, thought pneumonia an infectious disease, sometimes influenced by malaria, by unsanitary influences, and at other times by septic conditions. He had cases which did well by immediately instituting the tartar-emetic treatment with a blister. In apparently similar cases this treatment had little effect upon, owing to malarial complications, which rapidly disappear on administration of quinine. Again, the cases of septic origin he treated very successfully with whisky and milk.

Dr. H. D. Didama, of Syracuse, New York said that the microscope has demonstrated the micrococcus. What has it to do with pneumonia? Is it accidental? Experiments have shown that by inoculation they will produce the disease. In typhoid fever we have many types, yet they all are caused by the same kind of poison. Why could not this be also the case with the different forms of pneumonia? We ought to bear in mind that the latter disease has a regular course.

Blisters he approved in cases complicated with pleurisy.

Dr. James Stewart, of Montreal, Canada, inquired whether pneumonia was more frequent and also more fatal in malarious districts.

Professor William F. Waugh, of Philadelphia, thought it difficult for the disease in sparsely settled districts to be infectious.

Dr. Theodore H. Boysen, of Egg Harbor, New Jersey, practiced in a district where there was no malaria. Pneumonia in his section was a great rarity.

Dr. Geike replied that pneumonia was not more frequent, but more tedious and of a lower form, when complicated by malaria. It was also more fatal, in so far as it was of a lower form.

The Section then adjourned.

---

### SECTION ON GENERAL SURGERY.

William T. Briggs, M.D., Nashville, Tennessee, President.

Secretaries—Dudley P. Allen, M.D., Cleveland, Ohio; Karl Mayde, M.D., Vienna, Austria; J. R. Weist, M.D., Richmond, Indiana; Arthur H. Wilson, M.D., Boston, Massachusetts.

### First Day—Afternoon Session.

The Section was called to order at 3 P.M. by Professor Briggs, the president, who first welcomed the foreign representatives. He then said that this might be called the age of surgical activity. The whole field is undergoing a complete supervision, and this afternoon session would be devoted to the consideration of

### ABDOMINAL SURGERY.

The first paper on this subject was read by Professor Charles T. Parkes, of Chicago, Illinois.

Professor Parkes stated that the subject of gun-shot wounds of the abdomen, had of recent years been more generally understood; that he wished to place in review cases of other gentlemen and those coming under his own experience, so that deductions could be made. Up to 1885 the profession had not looked it in the face; but a few years ago surgeons were opposed to any interference. In 1884 the speaker read a paper upon this subject before the American Medical Association, since which time 36 cases have been reported, recovery taking place in 9 of them, and since coming here he had heard of two more, making 38 reported cases and 11 recoveries. The operation in these cases has been performed under all sorts of surroundings and the cases were not chosen ones. The writer considered that every case should be reported, whether resulting in recovery or death, in order to secure correct statistics.

The diagnosis with the abdomen unopened was at best uncertain, but when to open the future must decide. The writer referred to the cases of Drs. Bull, McEwen, Senn, Hamilton, Dennis and others already reported. He considered that a gun-shot wound with but one opening would give us hope that the ball might not have passed into the abdominal cavity; the location of the point of entrance and the direction the ball passed, together with weight and distance from which the ball was fired, materially increased the difficulty of diagnosis. The symptoms of shock and hæmorrhage were also important factors; also the natural temperament of the patient when in normal health. The writer considered that after an operation had been decided upon the incision should be made in the mesian line, as giving greater access to all parts of the abdominal cavity. If the ball passes up through the diaphragm he considered there was but little to be done. Shock he considered was no certain indication of perforation having taken place, and that it was a very difficult matter to know when to decline to operate, as many cases that have died have revealed at the post-mortem that the patient could have been saved had an operation been performed. The writer quoted two instances:

Case I.—J. S——, was shot in two places with a thirty-two calibre ball; he was seen three hours after. One ball went through his body, coming out at his back; he had eaten a hearty dinner just before the shooting. Considerable blood was found in the abdominal cavity, and five perforations were found, one being in the kidney. He seemed to do well for twenty-four hours, but then he died. It was found that death resulted from hemorrhage of the kidney. The speaker considered the kidney should have been removed at the time of the operation.

Case II.—P. J——, aged forty-five, was shot on July 4th. He was seen fourteen hours later, when a large wound was found in the iliac region. Respiration was thoracic, and the abdomen hard and tense. On opening the abdomen, fæcal matter was discharged and six inches of gut brought out; the wound was discovered to be very large. After the operation abdominal breathing was resumed. The patient died sixteen hours after the operation: had he been seen earlier the writer thought he would have recovered. He drew attention to the method of treating these wounds, where the mesentery was involved being much more difficult to treat. In these cases he was in the custom of uniting the wound diagonally, commencing the stitches at the mesenteric edge. He had never as yet found it necessary to put in more than one line of stitches, care being taken to draw the peritoneum together. Wounds of the liver should be sewn together with deep sutures. The spleen bleeds so freely and is difficult to suture, being so brittle. He thought it might be best to remove it, and this might also be necessary in wounds of the kidney.

The next paper was by Professor Nicholas Senn, of Milwaukee, Wisconsin, and entitled:

AN EXPERIMENTAL CONTRIBUTION TO INTESTINAL SURGERY, WITH SPECIAL REFERENCE TO THE TREATMENT OF INTESTINAL OBSTRUCTION.

Artificial intestinal obstruction stenosis.—(*a*) partial enterectomy and longitudinal suturing of wound. Traumatic stenosis from this cause becomes a source of danger from obstruction or perforation in all cases where the lumen of the bowel is reduced more than one-half in size Longitudinal suturing of wounds on the mesenteric side of the intestine should never be practiced, as such a procedure is invariably followed by gangrene and perforation by intercepting the vascular supply to the portion of bowel which corresponds to the mesenteric defect.

(*b*) Circular constriction of intestine. The immediate cause of gangrene in circular constriction of a loop of intestine is due to obstruction of the venous circulation, and takes place first at a point most remote from the cause of the obstruction.

2. Flexion.—(*a*) Flexion produced by partial enterectomy and transverse suturing of wound. On the convex surface of the bowel a defect an inch in width can be closed by transverse suturing without causing obstruction by flexion. In such cases the stenosis is subsequently corrected by a compensating bulging, or dilatation of the mesenteric side of the bowel. Closing a wound of such dimensions on the mesenteric side of the bowel by transverse suturing may give rise to intestinal obstruction by flexion, and to gangrene and perforation by seriously impairing the arterial supply to, and venous return from, the portion of bowel corresponding with the mesenteric defect.

(*b*) Flexion caused by inflammatory and other extrinsic causes gives rise to intestinal obstruction only in case the functional capacity of the flexed portion of the bowel has been diminished or suspended by the causes which have produced the flexion, or by subsequent causes independently of the flexion.

3 .Volvulvus.—As in flexion a volvulvus gives rise to symptoms of obstruction when the causes which have given rise to a rotation upon its axis of a loop of bowel have at the same time produced an impairment or suspension of peristalsis in the portion of bowel which constitutes the volvulvus, or when a diminution or suspension of peristalsis follows in consequence of the rotation.

4. Invagination.—Accumulation of intestinal contents above the seat of invagination is one of the most important factors which prevents spontaneous disinvagination, and which determines gangrene of the intussuscipiens.

Spontaneous reduction is not more frequent in ascending than descending invagination.

The immediate cause of gangrene of the intussuscipiens is obstruction to the return of venous blood by constriction at the neck of the intussuscipiens. Ileo-cæcal invagination, when recent, can frequently be reduced by distentions of the colon and rectum with water, but this method of reduction must be practiced with great care and gentleness, as over-distention of the colon and rectum is productive of multiple longitudinal lacerations of the peritoneal coat, an accident which is followed by the gravest consequences.

The competency of the ileo-cæcal valve can only be overcome by over-distention of the cæcum, and is effected by a mechanical separation of the margins of the valve ; consequently it is imprudent to attempt treatment of intestinal obstruction beyond the ileo-cæcal valve by injections per rectum.

Enterectomy.—Resection of more than six feet of the small intestine in dogs is uniformly fatal. The cause of death in such cases is always attributable to the immediate effects of the trauma. Resection of more than four feet of the small intestine in dogs is incompatible with normal digestion, absorption and nutrition, and often results in death from marasmus.

In cases of extensive intestinal resection the remaining portion of the intestinal tract undergoes compensatory hypertrophy, which, microspically, is shown by thickening of the intestinal coats and increased vascularization.

Physiological Exclusion.—Physiological exclusion of an extensive portion of the intestinal tract does not impair digestion, absorption and nutrition as seriously as the removal of a similar portion by resection.

Fæcal accumulation does not take place in the excluded portion of the intestinal canal.

The excluded portion of the bowel undergoes progressive atrophy.

Circular Enterorraphy.—A modification of Jobert's invagination-suture by lining the intussusceptum with a thin, flexible rubber ring, and the substitution of catgut for silk sutures is preferable to Czerny-Lembert sutures.

The line of suturing on neck of intussuscipiens should be covered by a flap or graft of omentum in all cases of circular resection, as this procedure furnishes an additional protection against perforation.

In circular enterorrhaphy, continuity of the peritoneal surface should be secured where the mesentery is detached by uniting the peritoneum with a fine catgut suture before the bowel is united, as this modification of the ordinary method furnishes better security against perforation on the mesenteric side.

INTESTINAL ANASTOMOSIS.

The formation of a fistulous communication between the bowel above and below the seat of obstruction should take the place of resection and circular enterorrhaphy in all cases where it is impossible or impracticable to remove the cause of obstruction, or where the pathological conditions which have given rise to the obstruction do not constitute an intrinsic source of danger. Gastro-enterostomy and jejuno-ileostomy should always be made by approximation with partially or completely decalcified, perforated bone-plates.

In making an intestinal anastomosis for obstructions in the cæcum, or colon, the communication above and below the seat of obstruction can be established by apposition with decalcified perforated bone-plates, or by lateral implanation of the ileum into the colon or rectum. An ileo-colostomy, or ileo-rectostomy, by approximation with decalcified perforated bone-plates or lateral implanation, should be done in all cases of irreducible ileo-cæcal invaginations where the local signs do not indicate the existence or occurrence of gangrene and perforation. In all cases of threatened gangrene and perforation the invaginated portion should be excised, both ends of the bowel closed, and the continuity of the intestinal canal restored by making an ileo-colostomy by approximation with perforated decalcified bone-plates, or by lateral implanation. The restoration of the continuity of the intestinal canal by perforated approximation plates, or lateral implanation, should be resorted to in all cases where circular enterorrhaphy is impossible on account of the difference in the size of the lumina of the two ends of the bowel.

In cases of multiple gun-shot wounds of the intestines involving the lateral, or convex side of the bowel, the formation of intestinal anastomosis by perforated decalcified bone-plates should be preferred to suturing, as this procedure is equally, if not more safe, and requires less time.

Adhesion Experiments.—Definitive healing ot an intestinal wound is only completed after the formation of a network of new vessels in the product of tissue-proliferation from the approximated serous surfaces. Under favorable circumstances quite firm adhesions are formed between the peritoneal surfaces within six to twelve hours which effectually resists the pressure from within outward. Scarification of the peritoneum at the seat of approximation hastens the formation of adhesions and the definitive healing of intestinal wounds.

Omental grafts, from one to two inches in width, and sufficiently long to completely encircle the bowel, retain their vitality, become firmly adherent in from twelve to eighteen hours, and are freely supplied with blood-vessels in from twenty-four to forty-eight hours. Omental transplantation, or omental grafting, should be done in every circular resection, or suturing of large intestinal wounds, as this procedure favors the healing of the visceral wound, and furnishes an additional protection against perforation.

The speaker presented some specimens in which the operation had been performed on dogs; these specimens fully illustrated the value of the method advocated, and its entire feasibility, the union in some of these specimens being most remarkable. He called attention to its use in stenosis of the pyloric orifice. The duodenum, or the first convenient coil of intestine being connected with the stomach by the method advocated, adhesions would form in from fourteen to twenty-four hours.

The adhesions in the specimens shown were wonderfully firm and strong.

---

### SECTION ON GYNECOLOGY.

Henry O. Marcy, M.D., Boston, Massachusetts, President.

Secretaries.—Georges Apostoli, M. D., Paris, France; Ernest W. Cushing, M.D., Boston, Massachusetts; Horatio R. Bigelow, M. D., Washington, D. C.; Carl Pawleck, M.D., Vienna, Austria

FIRST DAY—AFTERNOON SESSION.

The President omitted the formal address, and reserved the privilege of a more scientific contribution at a later date. He welcomed the foreign guests in the name of the profession of the entire country, and said that the Executive Committee had spared no effort to make their coming together one of marked pleasure and profit.

It seems, said Dr. Marcy, not invidious to mention the name of Marion Sims, honored by the world but doubly dear to America, with both pride and sorrow. With pride, because he conferred on our country the honor of being recognized as the birthplace of gynecology; with sorrow, since in the fulness of a ripe manhood he was all too soon taken from us, when we might have hoped his noble presence and eloquent speech would have welcomed you here instead of poor words of mine. May the memory of his gentle presence abide with us, and the earnest spirit with which he sought to know the truth find imitation in our councils.

Professor Nathan Bozeman, of New York, read a paper, entitled

ARTIFICIAL AND COMBINED DRAINAGE OF THE BLADDER, KIDNEY, AND UTERUS THROUGH THE VAGINA, WITH AND WITHOUT GRADUATED PRESSURE.

In this paper the author described an instrument which he had devised recently, This draws the uterine away from the mucous membrane, and in the most perfect manner. He has also been able to combine in the same instrument drainage with the dilatation of the cicatricial tissue of the vagina. The form of the instruments, which concerns us here, is intended for drainage alone, and I have called them intra-vaginal and vulvo-vaginal drainage supports. The intra-vaginal instrument is applicable in most cases to all positions of the body. The vulvo-vaginal form is suited to the recumbent position and to cases where the perineum is lacerated. These can be introduced and removed by the patient when necessary. They are small and simple, free from angles and sharp bodies, are readily kept clean, and excite no discomfort or irritation of the vagina. They do not press on the rectum or vagina, nor do they interfere with locomotion.

The author closed his paper with

THE FOLLOWING CONCLUSIONS:

1. The importance of completion of the operation of fistula has not been duly appreciated. This forms, in many cases, the principal difficulty in the successful performance of the operation for the closure of the fistular opening. In other cases, when the fistula is cured but the complications left without treatment, they lead sooner or later to the death or suffering of the patient. The greatest care should therefore be taken to discover and remove them.

2. Kolpokleisis, occlusion of the os uteri, and incision of the cervix in the bladder or rectum, are unjustifiable operations. They destroy the functions of the generative organs, lead to cystitis, then form venereal and vesicular calculi, pyelitis, and other diseases. Moreover, they are unnecessary. By means of the preparatory treatment of the complication by the aid of my button-suture, and my dilating speculum, I have been able to overcome all the difficulties which have been described as indications of operation.

3. The association of combined drainage in the dilatation of the vagina is a great improvement. The inconvenience and evil effects of incontinence of urine are thereby lessened and the evil effects are lessened, and the due relation of the treatment shortened by the more rapid healing of the incisions, and the formation of less cicatricial material in the reparative process.

4. We now propose a means of palliating the suffering due to incontinence of urine in a small proportion of cases of fistula which are incurable by this method—even the dangerous one of kolpokleisis. I believe that some form of drainage may be instituted in every case, and the patient may be thus restored to enjoy life and the performance of its duties.

5. The possession of a system of combined drainage will widen the scope of the operation of kolpo-cystotomy, done for cystitis, by removing the evils of incontinence of urine, now the chief objection to its performance.

6. Finally, I think the operation which I call kolpourethro cystotomy, followed by the exploration and treatment of the disease of the ureters and pelvis of the kidney, has a brilliant future before it. In the treatment of pyelitis, renal calculi, and obstruction of the ureters, it will restrict within narrow limits the operation of nephrotomy and nephrectomy.

Professor Grailey Hewitt, of London, England, was called on to open the discussion. He said it gave him great pleasure to take this occasion to visit America and meet those gentlemen whose efforts in the advancement of gynecology had elicited his admiration. The paper just read had shown decided advance in this line of thought. Conservative surgery, in this our day, seems to be running a race with operative surgery. He closed by endorsing the views of the essayist thoroughly.

Dr. Horatio R. Bigelow, of Washington D. C., read a paper on

CONSERVATIVE GYNÆCOLOGY.

The paper was divided into the following heads:

1. Plan and purpose of this communication.

2. The conservation of energy in its relation to conservative medicine.

3. What is meant by conservative gynæcology?

4. General medicine and its relation to specialism.

5. The tendency to operative measures a dangerous one. Experience gained at a great cost of life. Operations often performed unnecessarily.

6. Conservatism as applied to the treatment of uterine tumors. Recent results from the use of ergot and from the electric current. Exceptionally only do myoma call for laparotomies. The constitutional treatment.

7. Conservatism applied to tubal diseases and inflammations of the ovary. The psychic element. The subjective and objective symptoms. Rest treatment, electricity, Swedish movement, and bathing.

8. The relation of gynecology to the general environment of the patient. Minor disorders magnified into undue prominence by social factors in the every-day life of women. The treatment of such cases.

9. A consideration of oöphorectomy for hysteria, nervousness, and kindred disorders. Seldom warranted.

10. The restriction of abdominal surgery to men competent to practice it.

11. Closing remarks.

Dr. W. W. Potter, of Buffalo, New York, read a paper on

THE USE OF THE VAGINAL TAMPON IN PELVIC INFLAMMATION.

A married woman, twenty-two years of age, come under the author's care about a year ago, with a previous history of abortion, which happenad eight months before, from which time her invalidism dated. Upon his first examination, early in June, he found great tenderness of the intra-pelvic organs and tissues, with partial fixation of the womb. The left tube was enlarged, and presented a banana-shaped mass to the touch, while the surrounding cellular tissue was more or less hard and tense. This finally grew soft, and late in August pus was discharged through the uterus, after which the tube diminished in size. There was relief from pain, and the general health improved. The improvement continued until November, when the tube again grew tender and swollen; but another discharge of pus soon brought relief again. From this time onward the gain was uninterrupted, and she was dismissed cured on April 1, 1887.

The treatment consisted in the regular and systematic tamponement of the vaginia twice a week, copious vaginal lavements of hot water, frequently administered, and constitutional measures, principally of iron and arsenic. The vagina was insufflated just prior to each packing with iodoform, bismuth, mineral earth, or other powder, and then small pledgets of cotton, wool, or jute were introduced in sufficient numbers to make gentle, firm, and even pressure, as well as afford comfortable support. Though there was much more of detail, he said these were the essentials of the management, and their continuous employment for a period of nine months brought recovery.

Reference was made to the advancement made of late in the differentiation of pelvic diseases of women, whereby the domain of gynecological therapeutics had been so enlarged that it was no longer possible to group all the ailments of woman under the

INEXACT AND MISLEADING HEADS, "ULCERATION," "PROLAPSUS," AND "INFLAMMATION."

One of the important consequences of this more accurate classification of pelvic disease had been to invite surgical aid and interference in regions previously considered unsafe for the knife, and for maladies hitherto regarded incurable. The road to fame was so direct through the open gate-way of a brilliant abdominal section, that it was just possible that some ambitious men had removed an innocent ovary or unoffending tube, now and then, to obtain a "record" as spayers of women. He would not disparage the work of Battey, Tait, Hegar, Goodell, and others in this field, for he believed it of inestimable worth, nor would he deny that excision was often necessary; but, on the other hand, he thought the appendages might often be saved by timely and judicious management. The case reported, one of a number he had seen of a similar nature, was sufficiently typical to illustrate his purpose, and from which the following deductions might fairly be drawn:

1. That many cases of disease of the uterine appendages might be arrested in their progress and diverted to successful issue without operation by appropriate treatment resorted to in their earliest stages.

2. That the early employment of regular, prolonged, and systematic vaginal tamponement afforded one of the safest, surest, and simplest ways of preventing the ravages, in whole or in part, of the maladies in question, and of averting that mutilating of the sexuality of women consequent upon excision.

VAGINAL TAMPONNEMENT.

This occasion gave him the opportunity of offering some remarks upon the employment of vaginal tamponnement in the treatment of vaginal disease in general, a subject which was again creeping into medical literature through society discussions and papers.

In order to obtain the full benefits of this treatment, it was of the first importance that the packing be well done; that it be so placed as to afford ample support, give secure rest to the parts, make firm pressure, and not become dislodged during its wearing; while at the same time, it must not produce discomfort, interfere with the functions of the pelvic organs, nor cause irritation in the least degree.

In giving instructions as to its use he states the tampon must be multiple, and not made up of a single wad or mass, as was too often done.

For the past ten years he has made systematic use of the

KNEE-CHEST POSTURE

in the reduction of pelvic visceral displacements and of the multiple tampon in that connection, while latterly he had also employed them in the treatment of pelvic inflammations, the whole comprising many hundred cases. As a result of this experience he had reached the following conclusions:

1. In retro-deviations of the uterus the reposition of the organ should be made in the genu-pectoral posture without the aid of any other repositor than the finger; it should then be shoved up and held in place by the multiple tampon.

This treatment should always precede the employment of a pessary for a long or shorter period, according to the peculiarities of the case.

2. The foregoing applies with equally cogen force to prolapses and inflammations of the ovaries whenever these principles can be suitably adjusted to such cases.

3. In abrasions, erosions, and ulcerations of the os, in the hyperplastic womb, in subinvolution, incystocele, in rectocele, and in all conditions of disturbed or impaired nutrition of the pelvic organs, it affords a most efficient form of preparatory or curative treatment, tending to give the organs rest, restore their tone, deplete engorgement, remove blood stasis, improve locomotive power, and arrest retrograde tendencies in general.

4. In pelvic inflammations, whether of cellular, peritoneal, tubal, or other origin or involvement, it will often change their current or arrest progress, prevent suppuration or abridge its ravages, and thus often guide to a successful issue without a final appeal to a formidable and perhaps dangerous operation.

Dr. J. E. Burten, LL.D., of Liverpool, England, read a paper on the subject

WHEN SHALL WE OPERATE IN TUBAL PREGNANCY?

The author considered these operations dangerous to life, more so, perhaps than ovariotomies, while the diseases for which they are recommended and performed are, as a rule, not so. The operation is by no means a striking success therapeutically; many of the cases operated on are no better for it, some are even worse, while in good cases it takes at least twelve months for the patient to completely recover from its effects. We hear of "brilliant" cases that have taken two or three years before the "brilliant" results became manifest. It may be fairly assumed most of these would have recovered in that length of time without operation.

When the results are the best possible the woman is mutilated for life, an offence against conservative surgery as well as the first canon of medicine, *non nocere.* The mutilation entailed by the operation is particularly offensive to the sentiments of all civilized nations, and reduces a woman to the position of a female eunuch. Her loss and degradation (?) will certainly be remembered when the recollection of her sufferings has faded.

The objections to the operation being so grave, it ought to be performed only in justifiable cases after (1) prolonged treatment by less heroic and radical measures; (2) consultation with colleagues; (3) full explanation of the nature of the operation and its results to the patient herself and her nearest friend.

As regards the

OPERATION ITSELF IT IS JUSTIFIABLE

in:

1. Rapidly growing or bleeding myomata after other treatment patiently carried out has failed.

2. Pyosalpinx, if life is threatened by repeated attacks of peritonitis.

3. Chronic ovaritis (especially inflammation of the albuginea when Graafian vesicles burst through), when the pain is fixed and constant and months have been spent in unavailing treatment.

4. Parametritis, which, though it may not be dangerous to life at the time, may render the patient a permanent invalid.

5. Cystic degeneration of the ovaries, under the same condition as to pain as No. 4.

6. Neuroses distinctly of ovarian origin that have withstood years of treatment.

The operation is not justifiable in:

1. Myomata, except as noted.

2. Pyosalpinx, if the disease has become quiescent, if pain and fever have subsided and the pus has become inspissated.

3. Hydrosalpinx at any time, unless an associated perimetritis demand removal of the parts. A less radical operation will usually suffice.

4. Perimetritis, unless the disease promises to render the patient a permanent invalid.

5. Ovaritis, except under conditions noted.

6. Cystic degeneration of ovaries, except under conditions noted.

7. Hæmatocele and hæmatosalpinx under any conditions. Laparotomy and drainage may be called for, but removal of the organs, never. The same applies to ectopic gestation.

---

### SECTION ON MEDICAL CLIMATOLOGY AND DEMOGRAPHY.

Albert L. Gihon, M.D., Medical-Director U. S. Navy, President.

Secretaries.—Charles Denison, M.D., Denver, Colorado; Isambard Owen, M.D., London, England; A. Wernich, M.D., Coslin, Germany.

### First Day—Afternoon Session.

The opening address of Dr. Gihon, the president, was entitled

#### ON THE DOMAIN OF CLIMATOLOGY AND DEMOGRAPHY AS DEPENDENCIES OF MEDICINE.

A place was claimed for climatology as one of the sisterhood of medical sciences. The science must be taught in a manner befitting the importance of the subject. Preventive medicine, to which climatology and demography are contributory sciences, is more important than curative medicine. The views of the late Professors Austin Flint and Samuel D. Gross were quoted in support of this assertion.

The climatological study of the future must be based upon more rational methods of investigation. The mere recording of meteorological factors is not sufficient. Determinate climatic characters are not easy to formulate. Malaria is not a climatic disease, because the cause of malaria is a removable one and within the control of man, as proved by the experience in the marshes of Savannah, the lowlands of Holland, the Maremma of Tuscany, and the Roman Campagna, whose poisonous exhalations have been converted into innocuous vapors. The drainage of the Agro Romano has reduced the death-rate of the Italian army to one-third of its former magnitude. There are few specific climatic diseases. Local conditions of insanitation are more responsible for the production of diseases than the general influence of climate. By appropriate regulation of habits, clothing, and diet, the morbific effects of climate may be modified or averted, or its sanitary or therapeutic influence heightened.

Medical demography was defined to be that branch of medicine which is concerned with the study of vital phenomena among masses of men, rather than with individual diseases—the consideration of the effects of climatic and other influences upon collective man, embracing data as to race nobility, race fecundity, race morbility and mortality—the increase of a people or its decadence as from induced conjugal unfruitfulness, from introduced disease, etc. Thus Collective Investigation is its appropriate first chapter, and Vital Statistics is the fruit of this Collective Investigation, of which medical nomenclature is the instrument.

The data for future generalizations must be furnished by accurate and laborious collective investigation. Vital statistics must in future be something more than mere records of so many deaths, births or marriages. Morbility records must form the principal data for the vital statistics of the future. To have these records accurate, voluntary effort cannot be depended upon; they must be made under governmental authority. A rational nomenclature is a necessity, if our vital statistics shall serve as the basis of trustworthy generalizations. Mere symptomatological nosology is useless for statistical purposes.

Professor George H. Rohe, of Baltimore, Maryland, read a paper upon the

#### METEOROLOGICAL ELEMENTS OF CLIMATE AND THEIR EFFECTS UPON THE HUMAN ORGANISM.

The writer stated that medical climatology presented a much more complex problem than physical climatology. While the recorded observations of meteorological phenomena must form the basis, other conditions, such as those of the soil, must be taken into account. In studying the most characteristic climatic diseases, such as cholera, yellow fever, and epidemic dysentery, an intermediate factor—namely, the special virus of the disease—must be considered. A hot climate alone will not produce the diseases mentioned.

Sanatory or morbific effects are, however, produced by varying meteorological conditions. The effects of greatly diminished pressure upon the human organism are well known. Paul Bert and others have shown that these effects are not merely due to the physical condition of diminished pressure, but that the relative diminution of oxygen in rarified air is an important factor in their production. While cases of phthisis usually do well in a moderately rarified atmosphere, the effects of diminished pressure are not always beneficial, as has been pointed out by Dr. Loomis, who warns against the danger of sending patients with heart disease to high altitudes. It is not probable that diurnal or accidental variations of pressure have any appreciable influence upon health. Investigations conducted by the writer have failed to yield any positive results.

The primary classification of climates into tropical, temperate, and polar indicates the influence ascribed to temperature as a climatic factor. Although recent writers have attributed a determinate climatic influence to humidity, it is probable that this is of far less importance than some have supposed. The temperature must still be regarded as our best index of climate, but too much dependence must not be placed upon it. Many of the unfavorable effects attributed to moisture in the atmosphere ought to be ascribed to coincident insanitary conditions. The sanitary or morbific effects of air-currents have not been sufficiently considered as a climatological factor heretofore.

The tendency among climatologists at present is to deny to ozone any sanitary or disease-producing influence. Hydrogen peroxide is believed to be an antiseptic agent of importance in the atmosphere by some clinicians, who also ascribe therapeutic effects to the aromatic exhalations of certain plants. Very little of a definite character is known of the effects of these conditions. Further investigation is needed. The climatology of the future must be studied upon a broader basis, and ethnological, geographical, and epidemiological data must be taken into account before drawing conclusions.

Dr. W. T. Parker, of Newport, Rhode Island, read a paper on

THE IMPORTANCE OF THE STUDY OF CLIMATOLOGY IN CONNECTION WITH THE SCIENCE OF MEDICINE.

He animadverted upon the prevailing want of knowledge upon climatology among physicians. The science should be more widely studied. A number of health-resorts were mentioned as combining the requisite climatic with the appropriate sanitary conditions to qualify them as resorts for the sick. A wagon-trip across the plains was recommended as one of the best means to obtain the advantages of a climatic health-resort.

---

### SECTION ON MILITARY AND NAVAL SURGERY AND MEDICINE.

Henry Hollingsworth Smith, M.D., LL.D., Philadelphia, Pennsylvania, President.

Secretaries.—William Browning, M.D., Brooklyn, New York; J. McF. Gaston, M.D., Atlanta, Georgia; Eli A. Wood, M.D., Pittsburgh, Pennsylvania.

### FIRST DAY—AFTERNOON SESSION.

Professor Smith, the president, in his inaugural address referred to the greatness of our country, the climatic differences met with over its immense territory, its great extremes of temperature, its geographical characteristics, the large yearly immigration, and the peculiarities, social and physical, which would largely result from these conditions. He drew a graphic picture of the desolation of the alkaline plains of the West, with their inhospitable soil, in comparison with the more favored sections of our country, the fertility of the prairies, and their bountiful flora and fauna.

Referring to the social characteristics of the race, these were largely due, he said, to the struggles and hardships of the early settlers, who had a hard time of it in defending the soil reclaimed by great hardships and privations from its aboriginal inhabitants, who were constantly on the war-path against them. The speaker also adverted to the great opportunities that even a man born in the humblest of conditions has of attaining the highest round of the social ladder by energy and perseverance, and gave as examples the lives of some of our presidents. The soldier, he said, also had greater opportunities than in other countries in rising from a simple private to the highest rank, as was so well exemplified by scores of instances during the late war. The wonderful resources of the country in time of emergencies were alluded to, and comparison was drawn between the small regular army of the United States in 1861 to the enormous number of men enlisted up to 1865.

A large majority of these, he said, had returned to peaceful pursuits. The speaker adverted to the remarkable growth of the medical corps of the army, and of the enormous sum of over $47,000,000 having been expended for medical supplies during the late war. He feelingly alluded to the inestimable services of the late Professor Frank H. Hamilton, of New York, whose recent death was mourned by the nation.

The services rendered by American surgeons in the Crimean, Franco-Prussian, and other European wars were alluded to. The great value of the medical and surgical history of the war of Rebellion is admitted, he said, by all Europeans. The speaker then alluded to the great improvements made in the disposition of field hospitals, and to the desirability, which was being recognized, of treating the wounded under canvas, as their chances for recovery and freedom from complications were far greater under these conditions. The importance of selecting the proper site for such hospitals, sheltered from cold winds and naturally drained, was also touched upon.

The first paper was read by title, and was by Surgeon-Major Francis Patrick Staples, M.K.Q. C.P., Ireland, of the medical staff of Aldershot, England, entitled

A SHORT SCHEME FOR WATER ANALYSIS IN THE FIELD, IN CONNECTION WITH WHICH WILL BE SHOWN A SMALL PORTABLE CASE FOR REAGENTS AND APPARATUS.

The writer being absent, extracts were read from it by Dr. Marston, of England.

The second paper of the day, was entitled

ON THE NECESSITY FOR A MORE CAREFUL EXAMINATION OF THE WATER-SUPPLY OF MILITARY POSTS, WHERE AN UNUSUAL AMOUNT OF SICKNESS PREVAILS, AND EXAMINATION OF HYGIENIC SURROUNDINGS, by Morse K. Taylor, M.D., Major and Surgeon United States Army.

The author briefly reviewed the history of army sanitation in this country, and spoke of the alarming death-rate at some of the military posts, due mainly to enteric and malarial fevers; an improvement at these military posts could only be had by giving proper attention to the water-supply and general sanitation of such military posts. The author showed that very little attention had been given to these subjects, and that there had resulted a vast amount of sickness in the army. This sickness is remediable, as typhoid and malarial fevers are preventable; for it is shown that where they exist in excess, it is because of inattention or neglect to seek their cause and apply the proper remedies. Wherever efforts have been made to supply those military posts and camps with an abundant supply of pure water, there has resulted great improvement in the health of the soldiers and a consequent reduction in the mortality. Many cases were related, reinforced with copious figures, in proof of the author's assertions.

In the brief discussion which followed, Dr. William H. Lloyd, Inspector-General of the British Navy, spoke of the very great importance of an abundant supply of pure water at military posts. He had noticed a certain relation between the rain-fall of a region and the prevalence therein of malarial fevers, and that an increase in the development of these fevers was generally the immediate result of a great fall of rain in the region where these diseases prevail; the speaker was unable to explain the mysterious influence which is here at work. He also, in closing, alluded to the fact that analyses of water for the use of the British navy are regularly made, and that an improvement in the general health has been directly traceable to the pure water in use therein.

Dr. Joseph R. Smith, United States Army, read an able paper on the question of

THE BEST RATION FOR THE SOLDIER.

The author endeavored to show the difficulties in the way of ascertaining the exact amount of food that will suffice for the soldier in service, and compared the rations of different European armies, and the diets devised by various observers and experimenters. He showed that in general they err on the side of too great abundance, and briefly alluded to the proportion of carbon and nitrogen which the ration of the soldier should contain. He suggested two rations for armies, whose mean proportions of carbon and nitrogen would be about five thousand grains of the former and about three hundred grains of the latter. The paper was illustrated by numerous tables, showing the composition of the rations designed by Moleschott, Playfair, Pettenkofer, and Parkes.

In the absence of the author, Dr. John Denis MacDonald, of the British Army, his paper was read by Dr. Lloyd, of the Royal Navy. It described

A NEW FORM OF STRETCHER AND STRETCHER-SLING

for use in the field, which displayed great ingenuity. The stretcher-sling exhibited possesses the advantage of generally distritbuing the weight, and of combining the support of the shoulders, loins, and hips.

The next paper in order, by Dr. Valery Havard, United States Army, was read by title.

Dr. Jeffery A. Marston, of the British War Office, then read an instructive paper on

HUTS AND HUT HOSPITALS

as used by the British Army in Egypt and India. He described the construction of these in detail, and offered general remarks upon the subject of camp sanitation. A hut designed by Major Marsh, Royal Engineers, illustrated with plans and sketches, was especially recommended for its portability, durability, facility of erection and removal, and low cost. These huts are constructed with projecting eaves, and a constant change of air is effected by means of ridge ventilation.

The next paper entitled

THE CONSTRUCTION OF FIELD HOSPITALS, AS ILLUSTRATED IN THE DEPOT FIELD-HOSPITAL OF THE ARMY OF THE POTOMAC AT CITY POINT, VIRGINIA, IN 1864–65, was read by Dr. James Collins, of Philadelphia, Pennsylvania.

Beginning with some general remarks on the great advantages of tents for field hospitals, he described at length the construction and general disposition of the field hospital established in 1864–65 upon the shore of the Appomattox river, one mile from City Point, Virginia. The hospital occupied an area of two hundred acres, and had a capacity of 10,000 men, and there was treated therein the enormous number of 71,223 men. He alluded to the excellent sanitary condition of the camp, of the means adopted for sheltering the tents from the cold winds by the transplantation of small pine-trees, the various attempts made to heat the tents by ingenious devices, with more or less success, and to the organization of the hospital. In conclusion, the speaker feelingly adverted to a visit of the late President Lincoln to the camp, on April 12, 1865.

Dr. W. Varian, of Titusville, Pennsylvania, agreed with the writer in recommending tents for hospital use, dwelling upon the fact that by their use the liabilities to the development of enteric fever and other camp diseases was greatly lessened, and that wounds of all kinds, as a rule, do well under canvas. He emphasized the importance of allowing free circulation of air between the tents, and of making the streets of a width of fifty feet, at least. The various methods of heating the camps, spoken of by Dr. Smith, he had also tried, and found that open fires made in front of the tents was the most satisfactory.

Dr. Joseph R. Smith, U. S. Army, thought it was not an easy thing to decide as to the innocuity and harmfulness of a given water, for it was a well-known fact that many waters, even when containing a large amount of organic substances, could at times be used with impunity, while at others its use was attended with baneful results. It was impracticable, he said, to make use of the microscope in the field ; the eyes, nose and tongue of a soldier were his best guides, he thought, in the selection of water for drinking purposes, but there were times while in the field, when the soldier could not exercise his judgment in such selection, and must drink any water that can be found, irrespective of its color, smell or taste, and his drinking such water was not to be prevented. As to the water supply of military posts, he thought the surgeon was powerless in preventing the pollution of the water, particularly at posts situated within populous districts ; but that, in such cases, in public sentiment and appropriate legislation of the several states lay the only remedy.

Dr. Marston, of England, briefly reviewed certain epidemics which he had observed among soldiers, which were undoubtedly caused by the use of impure water ; the latter was not the only cause assignable, however, in the development of malarial fevers ; that the influence of freshly disturbed soil was apt to be overlooked. In certain malarial districts of China this influence was so commonly recognized that it is a popular belief, and one which would seem to be warranted by observation, that even so slight a disturbance of the soil as the scratching of the soil by domestic fowls in quest of food is sometimes followed by the occurrence of cases of malarial fever in the neighborhood.

In conclusion, the doctor called attention to the undoubted influence of water containing the salts of lime in solution in the causation of goitre and gave an illustration of an epidemic observed by him, when the disease was directly traceable to the use of such water.

---

### SECTION ON OBSTETRICS.

DeLaskie Miller, M.D., Ph.D., Chicago, Illinois, President.

Secretaries.—W. W. Jaggard, M. D., Chicago, Illinois; Joseph Kucher, M.D., New York, N. Y.; J. Williams, M.D., London, England.

### First Day—Afternoon Session.

#### president's address.

Professor Miller, in his opening address, extended cordial greeting and fraternal welcome to the distinguished guests present, and spoke of his appreciation of the labors of those connected with the section. The physician's labor was for the good of humanity, and all his achievements and inventions of utility, being generously added to the stock of general knowledge, would be ever preserved, for they became the property of the world. New rules and new applications were constantly arising; yet what rules, new to us, had been ages ago applied. We sought truth along ways beset with difficulties. A symptom might be accepted for a fact in pathology, while nothing was more variable—hence our deductions were liable to prove faulty. Yet no cavity should be too deep to deter us from direct investigation. We should not expect too much, and be not disappointed to find certain questions no nearer solution than they were one hundred years ago.

Sixty thousand obstetricians in the United States were anxiously awaiting true teachings, and our object should be to lead them through their numerous difficulties.

In speaking of certain obstetric difficulties, Professor Miller hoped that craniotomy in contracted pelves would be but rarely adopted and only in exceptional cases. It was too frequently assumed to be without danger to the mother. The inference from his researches was that the maternal mortality exceeds that reported. Under the new regime the interests of the child became more important. The requisite skill for other operative interference could now be found nearly everywhere.

In ectopic gestation diverse views on treatment prevailed. We have need of more concise rules. He considered early diagnosis of the greatest importance, and then electricity to arrest vitality.

While endeavoring to render the puerperal state aseptic, we should not fail to remember the danger from the ordinary agents used, especially where the kidneys were impaired, and he would not employ them in ordinary cases. Cleanliness was a most valuable means of asepsis.

In medicine we tolerated innovation and welcomed progress. We accept that which is fortified by experience and justified by results. The speaker then sketched the history of American obstetrics and obstetricians, beginning at the time of Samuel Guard, the family physician of George Washington, and ending on the verge of our own time.

The address concluded with the mention of those distinguished in the specialty who had died within the past year—Alfred H. McClintock, who in his last address had said, "My thoughts are with the dead;" the genial Alfred Meadows; and Carl Schroeder, one to be emulated, and whose memory should be cherished by all.

A paper sent by J. Braxton Hicks, M.D., F. R.S., of London, England,

ON THE CONTRACTIONS OF THE UTERUS THROUGHOUT PREGNANCY, AND THEIR VALUE IN THE DIAGNOSIS OF PREGNANCY, BOTH NORMAL AND COMPLICATED, was then read.

Fifteen years ago the author had first directed attention to the fact that the uterus contracted throughout pregnancy at intervals of from five to twenty minutes; since then he had added much to his previous knowledge.

Before the fourth month bimanual palpation was necessary, later external examination was sufficient for its detection. The pregnant uterus was very soft, and offered no appreciable resistance to palpation except during contraction. In a young girl suspected of pregnancy abdominal palpation was often all sufficient, though internal examination might be necessary. It was of great advantage to obtain decisive proof before making any allusion to pregnancy. A soft condition of the uterus with a localized lump, often pointed toward the death of the fœtus or to ectopic gestation. The uterus might contract about fibroids. A knowledge of the contractions often rendered easy a diagnosis otherwise difficult, as in ovarian tumor, ovarian tumor and pregnancy, ectopic gestation, and normal gestation, twin pregnancy, and hydramnios (palpation and the stethoscope as aids). With a dead fœtus the walls might be rigidly contracted. We should always look for corroborative signs.

Several cases were then cited in which the diagnosis was rendered certain only by this sign. The conclusions were:

1. That the uterus contracted at intervals of from five to twenty minutes during the whole of pregnancy, remaining contracted for from three to five minutes.

2. The uterus is firm when contracted, and the fœtus cannot be distinctly felt, though when the uterus is soft the fœtus is easily mapped out.

3. By noticing the contractions we are often enabled to diagnose normal pregnancy from other conditions.

4. The contractions have the physiological use of emptying the uterine veins of the carbonized blood.

5. The carbonized blood probably excites the contractions.

Professor Alexander R. Simpson, of Edinburgh, Scotland, thought the phenomenon of uterine contraction during pregnancy was now a widely recognized fact. We often met cases requiring all our diagnostic skill, and should employ all known means.

The sign mentioned was especially valuable before the fœtal heart-sounds could be distinguished, and in the third month when it could be employed in addition to Hegar's sign. One important result of these contractions was that when the uterus contracted forcibly, its contained blood was suddenly emptied into the surrounding parts, distending them, and thus favoring the dilatation of the parturient canal.

Professor A. F. A. King, of Washington, D. C. said there was sometimes difficulty in recognizing the contractions of the uterus, and they might be excited by polypi, by the retention of menstrual fluid, or by fibroids. They were principally of value after the third month. During the first and second months we had no positive means of diagnosis. In single women the diagnosis of pregnancy could not be certainly made by uterine contractions alone. An important point in searching for this sign was to irritate the uterus slightly to make it contract.

Professor Charpentier, of Paris, France, appreciated thoroughly the value of Dr. Hick's sign, and related a case of hydramnios where its presence made the diagnosis possible.

Duncan C. MacCallum, M.D., M.R.C.S. (England), of Montreal, Canada, read a paper on

VICARIOUS MENSTRUATION.

After a *résumé* of the literature of the subject and the diverse opinions of modern authorities, the reader cited four cases:

1st. Mrs. W——, aged thirty-eight; six children. Never nursed. Good health. Two months after birth of child had molimena and vomited blood. Treated by rest, ice, and gallic acid. No unpleasant after-effects and no further hæmorrhage for four weeks, when she again had molimena followed by hæmatemesis. At next period menses reappeared and have been normal since. Continued good health.

2d. Healthy woman; single. On the first day of a menstrual period was exposed to cold, and menses stopped; next day vomited blood; no vaginal discharge; regular since and healthy.

3d. Patient, aged thirty-three; healthy. First menstruation at fourteen years of age. Soon after had scarlatina, followed by amenorrhœa until eighteen. At twenty-three menstruation became very scanty and was accompanied by epistaxis for six periods, when it became regular again. Recently has again become scant and is accompanied by the epistaxis as before.

4th. Healthy women. Pregnant three months. Six weeks before had received a severe fright. Had a profuse hæmoptysis on two successive mornings, and three days later aborted. Four weeks later molimena and hæmoptysis, but since normal menstruation. Chest perfectly sound; good health In this case the ovum was killed six weeks before ovulation became established, and obstruction being offered to the usual flow, hæmorrhage took place from the weakest point.

To constitute vicarious menstruation there must be (*a*) absence of menstrual blood flow, (*b*) blood from some other organ than the uterus, and (*c*) no other assignable cause for the hæmorrhage than the increased premenstrual blood-tension.

A hæmorrhage under these conditions is truly supplementary and clearly vicarious.

Professor Charles T. Parkes, of Chicago, Illinois, mentioned a case occurring in a single woman, twenty-three years of age, sick eighteen months. For four months defecation had been at intervals of from one to seven weeks, and during this time only one ounce of urine had been passed daily. Severe fæcal vomiting. No normal menstruation for two months, but at the time for the periods molimena and vomiting of pure blood. Patient was a physical and mental wreck. Exploratory abdominal incision showed intestines filled with scybalæ. The ovaries, much enlarged, were removed. Urine at once increased to a pint daily, many scybalæ were passed, and general health rapidly improved. For two periods the patient had molimenæ and spat blood. Heart and lungs normal.

Dr. Opie, of Baltimore, Maryland, though cases of vicarious menstruation rare and illy defined. He believed that when the menstrual flow was impeded the vascular tension would seek relief at the weakest point.

Dr. Nelson, of Chicago, Illinois, recalled a case where bleeding from rectal hæmorrhoids occurred at menstrual periods, there being no uterine flow. The piles being cured and the cervix dilated, the menses appeared and gradually became normal.

Dr. Rodney Glissan, of Portland, Oregon, in thirty-nine years had seen three cases; in one menstruation had been regular, but after a serious illness hæmoptysis appeared every month and no vaginal flow for a year, when menstruation again became regular and the cough ceased. Now healthy.

Professor T. Lazarewitch, of St. Petersburg, Russia, presented a paper on

### THE MECHANISM OF LABOR AND THE NORMAL FORCEPS.

After calling attention to the factors concerned in the mechanism of labor, and the necessity of an accurate knowledge of the mechanics of the process, he described a forceps which he had devised, having straight parallel blades, and locking with a simple tenon and screw.

His conclusions were: 1. That forceps be considered as a continuation of the hand as feelers.

2. That the less the dimensions of the blades the better they could be guided.

3. That detrimental action increases with the size of the blades.

4. Convex margins should not be so thin as to cut, or so thick as to obstruct.

5. That the instrument should lock easily but allow slight longitudinal rotation of the blades.

6. Blades should be parallel.

7. Handles designed for convenience in guiding and the avoidance of injurious pressure.

8. Should be of smooth metal so as to be easily made aseptic.

9. That the pelvic curve was injudicious, detrimental, and difficult to employ.

10. That his parallel normal forceps filled all these conditions.

W. S. Stewart, M. D., of Philadelphia, Pennsylvania, exhibited an

### IMPROVED FORCEPS WITH PARALLEL BRANCHES.

The advantages claimed are: 1. That either blade may be applied first. 2. The impossibility of its slipping when properly applied. 3. Moderate and even compression, the degree of compression being regulated by the amount of resistance. 4. Great facility for making traction.

Dr. Opie thought that most forceps had merit in proportion to the skill and familiarity in their use by the individual operator. It was not so much the instrument as the man. We should not try to do by mechanism what the skillful hand may execute. A properly educated touch and hand were the best means of warding off dangers incident to the use of the forceps. He believed in the use of a moderate pelvic curve.

### SECTION ON OTOLOGY.

S. J. Jones, M.D., LL.D., Chicago, Illinois, President.

Secretaries—S. O. Richey, M.D., Washington, D. C.; H. B. Young, M.D., Burlington, Iowa.

### First Day—Afternoon Session.

Professor Jones, the president, in a brief address welcomed those present who had assembled from widely separated parts of the world to consider anew and together the long-neglected but important subject of Otology. He noted the advances in the last quarter of a century, and the effort that has been made, during that time, to remove the subject from the position which it so long held among the *opprobria* of medicine; that whilst much work had been done in the minute anatomy, the physiology and the pathology of the ear as well as its therapeutics, yet many open questions in otology remain, and he expressed the hope that the conferences during the congress might contribute materially towards the settlement of many of those questions. The need of a reliable and uniform standard for testing and recording the degree of acuteness of hearing, —one that should approximate in accuracy the tests now used for acuteness of vision—is universally recognized, and the subject should be considered during the sessions of the section. The value of bone-conduction as a test of hearing and a means of diagnosis between affections of the conducting and of the perceptive apparatus has not yet been fully established and was worthy of consideration by the section.

He noted the growing evidence of the general recognition, in the last few years, of the importance of the subject of otology as shown in the fact that a quarter of a century ago but few medical colleges in the United States gave special instruction on the subject, whilst now scarcely any of them, of recognized standing, are without competent instructors in otology, and the older institutions of other countries are affording more and better facilities for the study of the subject than were offered in former years. The influence of affections of the ear upon man's well-being in his intellectual condition, in his social relations, and as a member of the commonwealth were dwelt upon, and, in further evidence of the practical aspect of the case, in suppurative inflammation of the ear, the fact was cited that the danger to life of the sufferer excludes him from the benefits of life insurance, and governments will not admit him to their military or naval service, because of such risk to life or of unreliability for service.

Since abnormal conditions of the ear are often but a local expression of remote pathological conditions of the system. Otology, like ophthalmology, may often become a valuable aid in diagnosis in general medicine and surgery.

The first paper read was by Dr. S. S. Bishop, of Chicago, Illinois, being a statistical

REPORT OF 5,700 CASES OF EAR-DISEASE, CLASSIFIED BY AGE, SEX, OCCUPATION, AND DISEASE; CAUSATION; CONCLUSIONS:

1. Youth is a predisposing cause; more than one-fourth of the whole number of cases were under fifteen years of age.
2. Sex does not figure in the etiology of ear diseases.
3. About eighty per centum were chronic diseases, and about ninety per centum were diseases of the middle ear.
4. The causes of naso-pharyngeal catarrh are the proximate causes of middle ear disease.
5. The nervous temperament was predominant.

DISCUSSION.

Professor G. E. Frothingham, of Ann Arbor, Michigan, said that he had been much interested in the valuable statistical report presented by Dr. Bishop. It is upon carefully collected and carefully considered statistics, that we must largely rely for progress in the development of otology. The statistics presented by Dr. Bishop tend to establish the fact that those who live in large cities, especially if exposed to sudden changes of temperature, as are residents of Chicago, become more frequently affected with inflammatory conditions of the middle ear than those who reside in the country, since the institution from which the statistics were gathered receives patients from all parts of the state.

The statistics also show that a larger proportion of the foreign population were thus afflicted. He believed we might find an explanation of these facts in considering the important relation existing between naso-pharyngeal catarrh, and inflammatory affections of the middle ear, and the important factors in producing nasal catarrh. The air of cities is loaded, often heavily, with germs that have a tendency to excite inflammation in mucous membranes with which they come in contact. Their power to produce inflammation of the air passages, and even to produce phthisis, has long been recognized. That they may escape from this influence, we send patients who are afflicted or threatened with consumption, to a high mountain-region where the air is not only free from such germs but is also free from particles of dust and vitiating gases. The nasal passages serve the purpose of a strainer for the air passing to the lungs, and they not only warm and moisten the inspired air, but they partially free it from solid vitiating substances. These particles, lodged upon the mucous membranes of the nasal passages and pharynx, act as irritants, and so do many of the gases that are inhaled, in such an atmosphere. If we combine with this irritation the effect of a sudden change of temperature, by which the surface of the body becomes cooled, and the cutaneous exhalations are checked, we have the important factors in the production of naso-pharyngeal catarrh, and the consequent liability to middle ear disease. When such a sudden lowering of the temperature occurs, and the external surface of the body is not duly protected, the mucous surfaces most irritated are most likely to become involved in inflammatory action. Baron Larrey, who investigated the causes of the prevalence of conjunctivitis in Egypt, has ascribed it to a similar cause, namely, the irritation of the eye by exposure to dust and bright light during the day, and the exposure of the surface of the body to considerable change from the warm atmosphere, commonly experienced by day, to the cool atmosphere which prevailed during the night. He declared that those who protected themselves by proper covering at night, did not suffer from Egyptian ophthalmia, as did those who neglected this precaution. In the same way inflammation of the mucous lining of the intestines is produced more frequently when irritated by the ingestion of unripe fruit, and other indigestible food, during that period of the year when the days are warm, or hot, and the nights are cool, the body, commonly, being insufficiently protected from the chilling atmosphere.

That the germs lodged upon the naso-pharyngeal mucous membrane by breathing such an atmosphere play an important part in the production of the aural disease, Professor Frothingham is quite convinced, and that prompt and efficient treatment of the post-nasal catarrh is very important in the prevention and treatment of aural catarrh. In this treatment germicides are of great importance, as he had become convinced, by resorting to them for this purpose. He had found an application, by means of an atomizer, of a solution of bichloride of mercury, 1-2000 parts, one of the most efficient remedies for post-nasal catarrh, and he believes it acted thus beneficially, by destroying germs deposited from the air upon the naso-pharyngeal surface, which, in certain conditions of the system, might excite more reaction than at other times, when the vital forces of the patient might successfully resist their morbific influence. That the foreign population furnished the larger proportion of these cases is consistent with such an explanation. It furnished the larger proportion of laborers, and those who lived under bad hygienic circumstances, sleeping in small, illy-ventilated bedrooms, in which the atmosphere literally swarmed with germs.

Dr. R. Tilley, Chicago, Illinois, said: "I wish to express my appreciation of the presentation of the statistics relative to the study of otology. There is, however, in connection with their presentation, a factor which has scarcely received its due weight in the tabulation. It is stated that youth is a predisposing cause. Now, I fail to perceive that youth, in itself, can, under any circumstances, be a *cause* of any disease whatever. If, under certain circumstances, the youthful organism is ushered into the world under a decided disadvantage, and, on that account, certain diseases are developed, we must not say that it is the *youth* which is the cause. Relative to the percentage of cases of youth, in proportion to the cases of adult life, the important fact of the much larger proportion of children in the world relative to adults must not be forgotten ; but for the decease of a large number of the youthful members of the race, the proportion would be different. A similar observation should be made relative to those whose occupation requires them to spend their time, for the most part, in-doors ; unless we know the relative number of the individuals engaged in in-door and out-door work, the proportion of those afflicted is of comparatively small importance. I fail to find in the report, notwithstanding the special reference to occupation, any such occupations as unquestionably tax the aural tissues ; I refer to the vocation of boiler-makers. It might have been an advantage, also, to have referred to such as are engaged wholly with the telephone. If there were such care in the table it would be desirable to mention the absence of persons so engaged. Of the influence of a mere change of temperature from warm rooms to that of the external atmosphere, those who visit and live in such regions should furnish some evidence, which would contribute to the elucidation of the question.

Professor E. DeRossi, Rome, Italy: I agree perfectly with Professor Frothingham, that a very a large part of the diseases of the ear are caused by diseases of the nose and pharynx. Therefore I desire that the congress express wishes that studies on laryngo-rhinology be united with those on otology, rather than to unite this last one with ophthalmology, with which it has no connection, and by which nothing would be gained in the interest of scientific progress.

Dr. H. B. Young, Burlington, Iowa. As regards the influence of change of climate in the prevalence of ear diseases, it is questioned whether the sudden changes of temperature, with moisture, necessarily increases ear-diseases. Pointing to this is the fact, that during the past year the thermometric changes have been slight and the rainfall at the minimum, at least in the northern part of our country, and yet there has been an unusual increase relatively of ear troubles, both new, and relapsing old, cases.

Dr. Hobby, Iowa City, Iowa, endorsed the remarks of Dr. Young, and especially in the statement that the temperature was extraordinarily high and remarkably uniform in the Mississippi Valley during the last summer, and that there was a great increase in the number of diseases of the ear and nose, especially acute diseases, while the heat lasted, and before the evenings became cool.

Dr. G. W. Allyn, Pittsburgh, Pennsylvania, remarked that the warm winds from the Gulf of Mexico, and the cold winds from the great lakes made of Western Pennsylvania a perpetual battlefield. As a result, there were great and rapid changes in temperature and the moisture of this region, and if such changes were directly, or indirectly, the cause of middle-ear disease, these troubles should prevail there, and nose, throat and ear troubles are very prevalent there.

Professor J. F. Fulton, St. Paul, Minnesota, spoke of the increasing interest which is being taken in otology. He said that climate is not only a great factor in causation of diseases of the ear, but a most important one in the treatment and cure of such diseases; that many cases which could not be benefitted in one climate, may be cured by a change of climate. He referred to the favorable influence of a high and dry region in many forms of middle ear catarrh; referred to a distressing case of chronic suppurative inflammation, which could not be cured in its native climate, but which soon recovered by a change of climate.

Dr. S. O. Richey, Washington, D. C., said that in the moist climate of Washington, during the season of high temperature, catarrhs are frequent; not virulent, but, also, not very tractable, though slight. In the fashion of the day bacteria are made responsible for much of which they are doubtless innocent. They are mischievous, but nasal catarrhs *may* be due, entirely, to chilling of the surface of the body, with determination of blood to the more central and better protected regions.

Professor Frothingham said that he wished to correct the impression, if such had been created by his remarks, that he regarded all, or nearly all, cases of post-nasal catarrh, and consequent aural disease, as due to these inspired germs. What he did intend to assert, was his belief that this is a frequent cause, and that germicides should be more frequently used in the treatment, and that, as a germicide for these cases, a solution of the bichloride of mercury is, by far, the most efficient of any that he had used.

In closing the discussion of this paper Dr. Bishop said :—

In answer to the doubt expressed relative to my inference from the tables, that youth is a predisposing cause of diseases of the middle ear, I will say that since it is generally conceded that youth is a predisposing cause of exanthemata, as is illustrated in the cases of scarlet fever and measles; and, as it cannot be denied that a large proportion of ear affections are directly traceable to the diseases of childhood; and, in view of the facts that children are particularly prone to attacks of caryza, which is a proximate cause of tympanic inflammation; and, that a large percentage of this class of patients refer their ear troubles to the ear-aches and running ears of childhood; and, that the tables show that children, under the age of 15 years, constituted about 28½ per centum of the whole number of cases; it appears to be a logical deduction, that *youth is* a predisposing cause of diseases of the middle ear.

Professor Fulton remarked that one-half of all cases of middle ear diseases are associated with a catarrhal condition of the naso-pharynx. He resides in what is regarded as a high and dry part of our country, St. Paul, Minnesota; while the patients, who afforded the material for this paper live in a low, moist climate, lying between Lake Michigan on the East, the Mississippi River on the West, and the Ohio River on the South. Under these circumstances it is not surprising that a larger proportion than one-half of the patients, in the statistical report, were afflicted with one or another form of naso-pharyngeal catarrh. The most prevalent form was of a hypertrophic nature. There were frequent cases of enlarged tonsils, associated with granulations on the posterior wall of the pharynx, adenoid vegetations in the vault of the pharynx, and a thickened, roughened, red and boggy appearance of the mucous membrane generally, lining the pharynx and post-nasal space. In others, the tendency was not to cell proliferation, but to an atrophic condition. The columns of the fauces were extremely thin and pale; the membrane covering the posterior wall of the pharynx, pale, thin and shining, with tortuous vessels, in some cases dilated and injected with blood, and conveying the impression of a weakened state of the coats of the blood-vessels. This condition is quite often found in those individuals of middle age who have suffered much in early life from coryza and pharyngitis.

The second paper, by Dr. B. Loewenberg, of Paris, France, was on

THE TREATMENT AND THE BACTERIOLOGY OF AURAL FURUNCLES, and was read by Dr. S. O. Richey, in the absence of Dr. Loewenberg.

In 1880 and 1881, I published the results of my researches on the practical nature of aural furuncles, together with the theoretical and practical applications, the substance of which can be given as follows:

1. Boils are caused by an affection from outward, viz., through the ducts of the cutaneous follicles.

2. The successive outbreak of furuncles on the same individual takes place by auto-contagion, that is, through transport of the cocci upon the skin.

3. Infection from one person to others is possible, and originates from the same process as in No. 2.

These fundamental points have led me to a plan of treatment for boils and for furunculosis in general. I shall now expose this method with regard to the furuncles of the external ear, together with the results hitherto obtained.

My course of treatment is about the same as the one formerly proposed by me for otorrhœa, that is, the use of an over-saturated solution of one part of extremely fine powder of boracic acid to five parts of stong, even absolute, alcohol. This compound I use in installations into the meatus, to be repeated three to four times a day.

As long as the boil is not yet opened, a simple saturated alcoholic solution of boracic acid is sufficient, but when pus is already discharging I prefer the over-saturated solution, as it deposits a certain amount of boracic powder, dissolving gradually in the pus and thus exercising a continual anti-bacterial action.

Alcohol, besides its efficaciousness against micro-organisms, is moreover designed to facilitate the penetration of the compound into the ducts of the follicles, the seat of the disease. The fatty lining and contents of these capillary canals oppose, according to physico-chemical laws, a resistance to the entering of watery liquids, while alcohol, according to its affinity to fats, easily penetrates.

Incision of boils certainly sometimes facititates this course of treatment, but it is often very difficult to practice it just so as to divide the follicle-ducts, which seems to me the desideratum.

Cocaine, though applied upon the epidermis, often procures passing relief.

RESULTS.

An early application of the saturated solution of boracic acid in strong alcohol often arrests the boils; even in the cases where this abortive treatment should fail, the perseverant use of the over-saturated solution always stops the otherwise nearly unavoidable succession of boils, originated by auto-contagion, as I have called it (*loc. cit.*) These results seem to me of great importance, firstly, because aural furuncles are known to be extremely painful; secondly, because, according to my experience, the longer this local furunculosis lasts the greater is the tendency of the boils to form in parts situated nearer and nearer the drum, and consequently to prove more and more painful. These results to the best of my knowledge, have not been obtained before my researches.

Many female patients suffer, often for years, from aural boils arising before or during each menstrual period, a fact an explanative theory of which will be found in my paper. In such cases my treatment arrests these boils, or, at least, prevents their return. Nay, their formation may even be successfully prevented by a prophylactic use of this treatment begun before the catamenial epoch.

The same results can be obtained with persons who are regularly attacked by this affection in spring or fall.

BACTERIA IN EAR-FURUNCLES

I have undertaken bacteriological researches in a certain number of cases of still unopened boils of the meatus. In each case I first syringed this canal and then filled it for ten minutes with a luke-warm solution of bichloride of mercury ($\frac{1}{2000}$). A small parcel of the pus was inoculated into agar-agar or nutrient gelatine and plate cultivations were made of the whole.

I obtained the following results: The micro-organism most frequently found was staphylococcus albus, which was absent in only one case, then came staphylococcus aureus and sometimes staphylococcus citreus. Only in one case all these three staphylococci were traced together.

These results differ from those obtained by my friend N. Kirchner, from Wurzburg, who only found staphylococcus albus.

Dr. L. Turnbull, of Philadelphia, Pennsylvania, followed with a paper on

THE CAUSE AND TREATMENT OF AURAL FURUNCLES.

Professor G. E. Frothingham, Ann Arbor, Michigan, thinks the subject introduced by the two papers important, and that the paper of Dr. Loewenberg suggests, that the microbe is not yet "mustered out of service," as it were. Originally, Professor Frothingham said, he had been a skeptic as to the part that micro-organisms played in inflammatory affections, and still he can not give his consent to all the claims made in this direction. Careful observation of diseases, and the effect of anti-bacterial remedies had, however, convinced him of the great influence these organisms have in the production of various affections, and he believes we have not yet fathomed the subject, and that new developments are yet in store for us. The detection of these pathogenic organisms in aural furuncles by Dr. Loewenberg constituted a positive contribution to the subject, which we must accept, unless other sufficiently extensive observations should prove him mistaken. Dr. Loewenberg's theory of the production of furuncles would explain certain long-observed facts, which have before received no such plausible explanations. It had long been observed that furuncles have a tendency to appear in successive outbreaks, and that the new crop commonly appears near the part originally affected. Popular belief, the result of experience, exists, that the early opening of furuncles seems to conduce to this succession. One of the most experienced surgeons of his acquaintance, and indeed he himself, had come to that conclusion a dozen years ago. Professor Frothingham believes that, in the absence of antiseptic precautions, such early openings may lead to auto-contagion, and such precautions have not, heretofore, been taken. Some, it is true, might resist this contagion, as they do that of syphilis or gonorrhoea. General constitutional conditions and certain conditions of the nerve centres, play, undoubtedly, an important part, resisting bacterial action in some cases, allowing even exaggerated action in others. This is true of syphilis and other well.established contagions.

Dr. R. Tilley, Chicago, Illinois, said: In reference to the question of the influence of Micrococci in the production of furuncles, we must give full importance to the observations of Dr. N. Kirchner, of Wurtzberg, Germany.

According to Dr. Loewenberg's note, the observations of Dr. Kirchner demonstrated only the presence of Staphylococcus Albus in such cases, so that we have only the testimony of Dr. Loewenberg relative to the presence of the Staphylococcus Aureus, which is the important organism in question. Under these circumstances, while we give due importance to Dr. Loewenberg's observations, it would be advantageous to refer to the observations of other investigators in connection with the presence of Micro-cocci in the pus of unopened abscesses. Without looking up the question in particular, I am persuaded that the evidence is against the conclusions of Dr. Loewenberg.

The statement of the power of alcohol to dissolve the fat in the sebaceous follicles, requires more definite reference. Alcohol will dissolve fatty acid, but it will *not* dissolve ordinary fat. I have personally used alcoholic solutions of boric acid, with an addition of ether, to dissolve the fat in connection with suppurative conditions; not, however, on the furuncles. I refer, with some hesitation, to the use of the term "*Furunculosis.*" It is important, as scientific men, that we exert special care in the selection of appropriate terms. I am unable to see the special appropriateness of the term for the expression of the idea involved, and, if I am in error, I should be glad to be corrected.

Dr. Turnbull, in closing the discussion, said: "We differ from our distinguished friend, Dr. Loewenberg, in that, we believe that in the great majority of cases, boils depend upon some constitutional derangement of the system, more especially the blood, and arise in the system, not, as he states, 'by an affection from without; viz: through the ducts of the cutaneous follicles.' Secondly, that the number of these furuncles depends upon the defective system, and a loss in certain constituents of the blood, or, on the other hand, an increase of the fat globules, not alone 'by auto-contagion,' else the same results would follow on the skin of other parts of the body, when boils are opened. Thirdly, we have never known of a case of 'infection of one person by another.' In a recent instance of a boy in a family of six boys, no case occurred but one, and they slept and were in constant contact with each other; and in the severe case reported by me, the girl was one of a large family of girls, sleeping together, and yet no other case occurred. So far as we have been able to notice, no reports of any series of severe cases of furuncles have been given following out the treatment of Dr. Loewenberg.

In the use of boracic acid, we have found it to cause irritation, and sometimes small abscesses in and around the meatus. Boracic acid is one of the most feeble of the class of antiseptics; it does not compare with the bichloride of mercury, or even with carbolic acid. Incisions of a free character, the use of iron with the salts of potash, wine and, above all, nourishing diet to complete the cure, with change of air subsequently, is the one successful method of treatment for the relief of boils of the ear. No operation should ever be attempted upon the tender and sensitive ear, without the administration of an anaesthetic, like bromide of ethel, or the use of a five per cent. solution of cocaine, dissolved in one or two per cent. of pure phenol, or crystallized carbolic acid, and applied hypodermically.

### SECTION ON PATHOLOGY.

Alonzo B. Palmer, M. D., LL. D., Ann Arbor, Michigan, President.

Secretaries—H. M. Biggs, M.D., New York; Isaac N. Himes, M.D., Cleveland, Ohio.

FIRST DAY—AFTERNOON SESSION.

Professor Palmer, the President, in his inaugural address, discussed the progress of medicine toward exact science.

Dr. George R. Elliott, of New York, N. Y., read a paper on

PRESSURE PARALYSIS OF POTT'S DISEASE, in which he reported a case and presented the specimen showing pressure of the cauda equina, accompanied by secondary ascending degeneration of the posterior columns of the spinal cord.

The author's conclusions were based upon (1) experimental physiology; (2) pathological findings; and (3) clinical manifestations. They demonstrated that the lesion is a mechanical one in the majority of cases, and not a true myelitis, as ordinarily described—the latter being the lesion in only a small number of cases. They further demonstrated how circulation is interfered with and how such interference leads to the production of many forms of the paralysis recognized clinically. The specimen was interesting in showing the compression lesion of the cauda equina, and as probably being the first case reported where the pressure of the cauda equina, leading to secondary degeneration, was due to spinal caries.

Diagrams were used to illustrate the points made.

### SECTION ON PSYCHOLOGICAL MEDICINE AND NERVOUS DISEASES.

Judson B. Andrews, M. D., Buffalo, New York, President.

Secretaries—G. A. Blumer, M. D., Utica, New York; M. le Dr. A. Bouchereau, Paris, France; E. D. Ferguson, M. D., Troy, New York.

FIRST DAY—AFTERNOON SESSION.

The Section was called to order by the president, Dr. Andrews. After the reading of the list of Vice-Presidents and Secretaries, the President delivered his address, entitled,

THE DISTRIBUTION AND CARE OF THE INSANE IN THE UNITED STATES.

After delivering an eloquent eulogy on Professor John P. Gray, who was the first president of this section, he said: The amount of insanity bears a close relation to the duration of the social and governmental life of the people. Dividing the country into two great belts of north and south, there is an almost regular proportionate decrease of lunacy as w leave the older settled parts of the country along the Atlantic coast, till we reach the extreme western slope.

The New England states lead with one insane person to every 359 inhabitants. This decreases until we reach the newer states and territories with one insane person to every 1,263 inhabitants. In the seaboard states of the southern belt we have one to every 610 inhabitants, and the extreme Southern States with but one to every 935 of the population. This emphasizes the statement that the pioneers of our newer settlements are the more hardy and vigorous citizens, and that the feeble and dependent are left in their former homes to enjoy the comforts of the hospitals and asylums, which are the special growth of the older civilization.

New York is the first Irish city in the world, and Berlin and Hamburg are the only cities which contain as many Germans as our own metropolis. We have among the negroes in the United States one insane person to every 1,097 of inhabitants. In the negro race the proportionate increase of insanity is far greater than in any other division of the population. From 1870 to 1880 there was an increase in the census of the colored race of 34.85 per cent. while for the same period there was an increase of 25.8 per cent. of the insane. This large multiplication has occurred since emancipation from slavery, and the consequent change in condition and life. The causes are briefly told—enlarged freedom, too often ending in license; excessive use of stimulants; excitement of the emotions, already unduly developed; the unaccustomed strife for means of subsistence, educational strain, and poverty. Among the Chinese and aborigines there has been but a small increase of insanity. There is among them less of the refinements of civilization, less competition and struggle for place, power, or wealth, and, as a consequence, less tendency to mental deterioration.

Dr. Andrews presented a list of state institutions in the United States, number of patients in each, and also the number of medical officers attached to each institution. One hundred and twenty-one asylums were represented, fifteen of these having been built since 1880. In 1880 there were 39,093 patients in these asylums. In 1886 the number had increased to 61,411, an annual increase of nine per cent. At the present rate of increase, before the end of the decennial period we shall have 75,000 in our asylums.

Dr. Andrews next referred to the diversity in lunacy laws in the various states of the union, comparing them with the general governmental laws of Great Britain. Special accommodation for the criminal class—a highly desirable innovation, has been entered upon by two states only—New York and Illinois.

With the exception of Delaware and Vermont, every state in the Union has adopted the state system of caring for the insane, and New York was the first state to erect separate asylums for the chronic insane. Asylum architecture has undergone material changes during the past twenty years, the plan now most generally adopted being that of building detached blocks of structures in different parts of the asylum grounds, within easy reach of the administration building, these to accommodate the feeble and helpless, the epileptics and acutely maniacal patients. In many portions of the country, buildings separate and complete are joined by connecting fireproof corridors.

The President next outlined some of the important changes occurring in the interior management of asylums at the present time. Among them, the introduction of electricity for lighting purposes, of direct radiation for heating purposes, and of natural ventilation as taking the place of the blower-fans much in vogue in former years.

A higher medical standard is to be noted in the asylums of the United States. The medical officers are in very few cases appointed through political favoritism, and in the state of New York the civil service requirements insure the sound medical equipment of assistant physicians. Oöphorectomy is now recognized as a legitimate mode of treatment, and castration in appropriate cases has, at present, some able advocates ; electricity is now being used with more intelligent knowledge of its power. Its hand-maid, massage, less powerful and less mysterious, but not less practical, has gained a position of prominence in the treatment of insanity in many institutions. The experiments in mesmerism, mind-reading, and the faith-cure have led to a closer investigation into the relation between mind and body, with a result of finding in expectant attention a valuable and legitimate help in the treatment of mental diseases.

D. Hack Tuke, M. D., F.R.C.P., London, England, sent a paper, which in his absence was read by Dr. G. F. Blandford.

THE VARIOUS MODES OF PROVIDING FOR THE INSANE AND IDIOTS IN THE UNITED STATES AND GREAT BRITAIN, AND ON THE "RAPPROCHEMENT" BETWEEN AMERICAN AND BRITISH ALIENISTS IN REGARD TO THE EMPLOYMENT OF MECHANICAL RESTRAINT.

It was largely a comparison of the systems pursued in both countries. At the last official census there were 80,000 idiots and insane in England and Wales, 72,000 of them being paupers and 8,000 belonging to the private class. In the workhouses of England fourteen per cent. of the paupers were cared for, and Dr. Tuke believed well cared for. In some of the workhouses there are very good lunacy wards, and these, in his opinion, provided far better for this class than do American almshouses. The Doctor thought well of the increasing tendency in this country to segregate the insane, as shown at Willard and Kankakee. He deplored the fact that so many patients were still retained in the almshouses of the United States, and instanced the abuses in the Cook County Asylum at Chicago. The Doctor thought the plan now being adopted in Massachusetts of boarding the chronic insane in private families was attended with considerable danger to the families, and might also result in overcrowding of patients, but, as a whole, commended the new departure.

Dr. Tuke concluded with remarks upo then little difference at present existing in the practice of British and American alienists, as regards non-restraint. He said that the system of Connollyism or strict non-restraint was not a feasible one in either country, and was glad to know that in the United States judgment had been used in the use or none-use of restraint, instead of blindly clinging to an ideal.

Dr. Savage, of London, England, said that he was glad to know that enlightened American superintendents had not tried to carry out the principles of strict Connollyism. There could be no absolute non-restraint so long as asylums existed, because life in an asylum in itself meant restraint. It was a mere matter of degree. The humane treatment of insane patients was in direct relationship to the civilization of a country. Let the principles of non-restraint be preached at all times, and let your attendants know this ; otherwise you will have trouble. Youthful alienists might preach the doctrine of Connollyism pure and simple, but so high an ideal could not be attained so long as the preservation of society and the preservation of the patient's life is to be considered.

Dr. Andrews said the practice of American alienists was for the minimum of restraint in all cases. In former years there had been in the minds of Americans a misconception of the real practice of their English brethren. A study of the Blue Books, however, had served to show that strict non-restraint—Connollysm—had not been carried out in Great Britain. There had been considerable feeling manifested in the past between English and American mental specialists upon this topic, and Dr. Andrews was glad to say that as a more perfect understanding had come in regard to English practice, there was found to be little actual cause for dispute or disagreement.

The last paper of the day was read by Dr. Henry M. Hurd, of Pontiac, Michigan, on

THE RELIGIOUS DELUSIONS OF THE INSANE.

He first treated of the religious delusions which accompany the mental development of over stimulated and injudiciously educated children. These delusions are apt to take the form of morbid fear. When the child fails to derive pleasure and an emotional glow from prayer or praise, he fancies that some duty has been neglected or improperly performed, and is tormented by doubts and overwhelmed by remorse. An interesting case was then given by Dr. Hurd. The next class treated of by the author of this paper were patients suffering from the religious delusions of the insanity of masturbation. In the beginning morbid fear is predominant, and a study of the Bible and a habit of introspection is present. The patient is scrupulous in all religious observances, and there is a silly vanity in religious matters. Hallucinations are present. The patient becomes egotistical in his religious ideas. Dr. Hurd gave an illustrative case of this form of mental disorder. The patient had striking hallucinations of hearing, and constantly communicated with invisible persons. The doctor next passed to the religious delusions of paranoia. In the congenitally abnormal class referred to there is, as early as puberty, a precocious sexual excitability which gives rise to an unnatural religious susceptibility. The patients have a strong religious bias and are apt to embrace peculiar views, or to be attracted by the latest novelty in religion. The immediate cause of the development of religious delusions is generally some physical ailment or shock. One patient, after praying for several nights in a cornfield, in great agony of mind, felt the burden of sin fall from his aching shoulders and saw it glide away in the darkness, dark, sinister, and, to use his own expression, "like a small woodchuck."

Dr. Hurd cited many interesting cases, taken from the records of his asylum, as illustrative of this form of mental disease.

Dr. Hurd said the connection between sexual impulses and the development of religious delusions was not from any association of ideas, but rather from the close association and inherent unity of emotional states. The religious delusions of over-stimulated children are generally relieved by rest, freedom from study, and a judicious correction of the educational errors which produced them.

---

### SECTION ON THERAPEUTICS AND MATERIA MEDICA.

Traill Green, M.D., LL.D., Easton, Pennsylvania, President.

Secretaries—Frank Woodbury, M.D., Philadelphia, Pennsylvania; Alfred S. Gubb, M.D., London, England; L. Lewin, M.D., Berlin, Germany; F. Dronke, M.D., Berlin, Germany.

### FIRST DAY—AFTERNOON SESSION.

Professor Green, the president, delivered a brief address at the opening of the session, which dealt chiefly with the history of the development of the study of therapeutics and materia medica, especially referring to the labors of Dr. John Morgan, of Philadelphia, who delivered the first lectures upon the subject in this country. The instruction which he gave was based upon the teachings of the University of Edinburgh, and its traditions have largely influenced medical education in this country.

Dr. Phillips, of Ventnor, Isle of Wight, Vice-President of the Section, occupied the chair during the opening address, and at its conclusion returned thanks for the interesting remarks, and said that English therapeutists felt under great obligation to Wood, Dunglison, Stillé, and other American workers in this field. He referred to his recent labors upon the subject of the action of diuretics, the results of which he had embodied in a paper which he is shortly to read before this section.

Dr. J. M. G. Carter, of Waukegan, Illinois, presented a "Synopsis of the Medical Botany of the United States," in which 140 orders, 620 genera, and over 1,300 species are described. The study of these is much simplified by the knowledge that the different species of the same plant resemble each other in therapeutic effect, differing principally in degree. There are similar differences in the same species, according to the conditions under which the plants are grown. Vogel has pointed out that *conine* does not appear in conium plants grown in Scotland; cinchona plants grown in hot-houses do not produce quinine. The mistletoe and the black haw he considered the most valuable additions recently made to our therapeutics, the former in urethral, the latter for uterine, irritation.

Dr. Coghill had been much impressed by the value of some American indigenous drugs, but he pointed out the necessity of better appreciation of their physiological effects in order to ascertain their real value in therapeutics.

Dr. Phillips said that with reference to one drug mentioned, grindelia, he had obtained excellent results, and had a long series of notes of its successful use in asthma and emphysema with dilated heart.

Dr. William Murrell, of London, had noticed a great variation in the strength of drug preparations; as the rule, the samples sent for trial were made with more care than the ordinary article supplied on prescription.

Professor Frank Woodbury, of Philadelphia, mentioned the employment of mistletoe in weak heart, and in post-partum hæmorrhage, owing to its effect upon unstriped muscular tissue. Rhamnus purshiana he considered a useful substitute for rhubarb, and equally efficient as a cholagogue.

Dr. F. E. Stewart, Wilmington, Delaware, read a paper entitled,

A PROPOSED INVESTIGATION OF THE MATERIA MEDICA OF THE WORLD BY THE GOVERNMENT OF THE UNITED STATES.

in which he advocated governmental supervision over novel therapeutic agents, and the placing of some restraint over the commercial enterprise of drug manufacturers, who introduced new drugs at extortionate prices. He also denounced the proprietary medicine business as an abuse of the copyright privilege. He suggested the establishment of a bureau for the examination of proposed new remedies, proprietary or otherwise, by the government, and to include a supervision over the entire drug supply of the country.

Dr. H. H. Rusby, by invitation, opened the discussion. He considered the present a very opportune moment for agitating such a measure as the paper advocated. By a combined effort of the physicians and pharmacists, a committee might be appointed to improve the botanical work done under the direction of the United States government. Hitherto, the amount appropriated had been lamentably inefficient, and had been carried on under the department of geology ; it should be transferred to the department of agriculture. If the International Medical Congress were to pass a resolution calling upon Congress to give adequate means and energy to the botanical work, its value would be greatly enhanced, and its importance in a medical point of view would be very great.

Some remarks were made by different members, denouncing the patent medicine abuse in this country.

Dr. Stewart, in closing the discussion, said that a patent was never intended to secure unlimited secrecy, but on the contrary, to insure publication of an invention after a period of time. Moreover, the so-called patent medicines are not patented, and cannot be patented ; they are sold under a misapplication of the copyright law.

---

### SECTION ON LARYNGOLOGY.

William H. Daly, M.D., Pittsburgh, Pennsylvania, President.

Secretaries.—William Porter, M.D., St. Louis, Missouri ; D. N. Rankin, M.D., Alleghany, Pennsylvania; Ottakar Chiari, M.D., Vienna; Hermann Krause, M.D., Berlin, Germany; E. G. Moure, M.D., Bordeaux, France.

### AFTERNOON SESSION.

The Section was formally opened at 3 P.M. with

THE INAUGURAL ADDRESS OF DR. DALY, THE PRESIDENT.

It was most gratifying, he said, to see so many gathered together from all parts of the Union to welcome our guests who have come from afar, and to redeem the pledge made to them at Copenhagen in 1884. It was moreover a source of the highest pleasure to meet so many of those from foreign lands, whose names are far too familiar to admit of their being regarded as new acquaintances. We appreciate the honor of entertaining such representative men, and extend to them our heartfelt greeting, and cordial welcome to all.

The President then called attention to the status of laryngology in 1876, compared with its present advancement. In rhinology much has been done for the successful treatment of hay-fever since the appearance of a paper by the president, in 1881, upon the predisposing intra-nasal causes of this disease. Now a large percentage of those who formerly fled to the mountain tops in search of relief, may be cured at home by proper and rational surgical treatment. The lamented Hack and other able workers claimed more from this plan of treatment than he then advised ; but seven years of observation strengthen his belief that a large proportion depend upon a chronic intra-nasal disease, without the presence of which pollen, dust, and other agents are innocuous. A hope for the future is that more perfect local treatment may cure a still larger percentage.

The laryngologist of the future must give more attention to the nasal passages, and the rhinologist must be more of a surgeon than a physician. Who has not noted the rapid improvement of puny children following the treatment of enlarged tonsils and hypertrophied tubinated tissues, so as to give free breathing space? Many have with him verified by years of experience that a large proportion of inflammatory diseases of the larynx are secondary to intra-nasal diseases of a like character. The aid that modern rhinology extends toward the treatment of diseases of the internal and middle ear is appreciated by every aurist.

The President called attention to our indebtedness to the persevering work and ability of the now silent members, such as the late Elsberg,

Krishaber, Foulis, Bruns, Waldenburg, Burow, Bocker and Hack.

The first paper was read by Dr. Richard H. Thomas, of Baltimore, entitled:

A CONTRIBUTION TO THE CAUSES OF SO-CALLED HAY-FEVER, NASAL ASTHMA, AND ALLIED AFFECTIONS, CONSIDERED FROM A CLINICAL STANDPOINT.

The term hay-fever he uses, not because scientifically correct, but because it is well understood and convenient. The factors that appear to enter into the causation of hay-fever and other nasal neuroses are:

1. The general nervous system. Although a neurasthenic condition is so often present with a nasal, neurosis, the frequency of its occurrence, without reflex phenomena associated with the upper air-passages, shows it inadequate to explain the phenomena.

2. The theory that the condition of nervous system is similar to epilepsy has not been proved.

3. The intra-nasal condition is usually that of hypertrophy, or such disease as polypi, deflected septum, etc., tending to more or less nasal obstruction.

The author has noted, however, (*a*) that nasal obstruction in most cases is not associated with reflex phenomena, and even in neurasthenic individuals, as Dr. Bosworth, of New York, claims, only the " proper pollen " is needed to produce hay fever. One unique case improved as occlusion advanced. (*b*) Obstruction is not first symptom in asthmatic manifestations, nor is it always present. (*c*) Cases have been reported of reflex phenomena associated with atrophic rhinitis. Morrell Mackenzie has seen cases where there was nothing abnormal beyond hyperæmia, and author corroborates as far as nasal asthma is concerned. (*d*) Temporary attacks are brought on by touching sensitive areas, independent of nasal obstruction.

4. The author has seen cases where hay asthma followed chronic nasal trouble, and other cases where it apparently preceded structural change. Special sensitive areas are claimed to exist in the upper air passages. Irritation, disease, hypertrophy, polyp, etc., gives rise to reflex phenomena. They are variously located by different authors on septum and turbinated bodies. When all are considered, there is very little mucous membrane that is not included.

5. After experimenting, Dr. Thomas concluded that the sensitive areas varied greatly with different individuals. Examinations were made between the attacks. Sensitive areas on one side at times bear no relation to those on the other. He could not decide which portions of nasal tract were most sensitive, unless it were septum. The favorite location is that part supplied by the olfactory. Phenomena noted were paroxysmal sneezing or cough, and temporary asthmatic dyspnœa, headache, etc.; none where mucosa was healthy.

Reflex sensitive areas are found in hay-fever, but not in normal nose.

6. Modern research shows that the nervous element enters to large extent. A peculiar condition either of nerve centers or endings gives perverted action under certain forms of irritation. He agrees with Lublinski that the olfactory, trigeminus, and sympathetic are involved in producing a paroxysm. No such disturbance is caused by pathological conditions until exciting cause is present.

Exciting causes.—(1) Inert substances floating in air, dust, pollen, etc.; (2) psychical impressions; (3) metêorological changes, sunlight, wind, etc.; (4) morbid changes or growth; (5) irritation reflected from distant part of body.

Any of these may give rise to paroxysm when idiosyncrasy exists, not otherwise.

Pollen of Indian-corn, ragweed, luxuriant vegetation, smell of flowers, flour, meal, dust, odors of close room, where oysters have been opened, dissecting-room, dust of tobacco, smoke, etc.

Mental impression and often sympathy with another who is afflicted will cause an attack.

Hereditary tendency is often noted. What affects one may have no effect on another.

Treatment.—Remove patient from irritant or irritant from patient if internal, as when polyp, etc. Irritation of distant organs should be treated. It is often difficult to account for the curative effect of change of locality.

Use of cocaine gives temporary relief, but there is danger to tissues from protracted use. Powders are of doubtful benefit. Flannels give some relief. Radical treatment is called for. If nervous tissues are at fault, treat accordingly. Iron, quinine, arsenic, hydrobromic acid (Gardiner's syrup), valerianate of zinc, etc., build up health. We are indebted to Dr. Daly for the introduction of local treatment of hay fever. Cure co-existing nasal disease and cauterize sensitive areas. Galvano-cautery preferable to acetic acid or chromic. Avoid destroying too much tissue. Prevent pain by cocaine and follow up with Dobell's solution. Carry on treatment between attacks and leave no sensitive areas untreated. Each patient requires separate study.

---

SECTION ON PHYSIOLOGY.

John H. Callender, M.D., Nashville, Tennessee, President.

Secretaries—R. W. Bishop, M.D., Chicago,

Illinois; Bernard Fraenkel, M.D., Berlin, Germany; Randolph Barksdale, M.D., Petersburg, Virginia.

FIRST DAY—AFTERNOON SESSION.

In his inaugural address, Professor Callender, the president, directed special attention to the influence exerted by the cell on the processes of development and decay.

Dr. Daniel G. Clark, of Toronto, Canada, read a paper on

THE BASAL GANGLIA OF THE BRAIN AS CENTERS OF PSYCHIC AND FUNCTIONAL POWER.

The author of the paper maintained that these ganglia are psychical centers: (1) because of their greater activity physiologically; (2) because they are the focal centers to the hemispheres; (3) they are vital points of greater significance than any other part of the brain, and (4) experiments point to their directing and controlling power.

The paper gave rise to discussion, which was participated in by Drs. Love, Wythe, Stockman, of Edinburgh; Kleinschmidt, of Washington; Hallibert, Professor Madden, and Boenning, of Philadelphia.

Dr. Richard Caton, of Liverpool, England, then read a paper entitled

RESEARCHES ON ELECTRICAL PHENOMENA OF CEREBRAL GRAY MATTER.

These were the chief results obtained. Owing to the great difficulty of the investigation, more than half of the experiments were valueless.

1. Electrical currents are present in the gray matter of the convolutions.

2. These currents are increased during the arrest of functional activity caused by anæsthetics or death. After death the current diminishes and disappears.

3. In regions of the brain related to a special function, negative variation appears to take place during functional activity.

4. The occurrence of negative variation in an area of the brain assumed to be related to a special act at the movement of the performance of the act affords further evidence of localization.

5. These experiments afford some evidence that areas of brain related to movements of special muscles are also related to some form of sensibility in the skin adjacent to those muscles.

---

### SECTION ON DISEASES OF CHILDREN.

J. Lewis Smith, M.D., New York, President.

Secretaries—I. N. Love, M.D., St. Louis, Missouri; Henry Coggeshall, M.D., Mount Vernon, New York; Dillon Brown, M.D., New York; Lucien Darvansville, M..D, New York.

FIRST DAY—AFTERNOON SESSION.

Professor Smith, the president, in his opening address, referred to the practical value of the papers about to be presented, and congratulated the section on the large number of papers which had been contributed by prominent men in active practice in England, Scotland, France, Germany, and South America. The subject of intubation of the larynx would be considered in several papers. Important contributions in this connection would be read from M. Bouchut and Dr. O'Dwyer. The name of the former will always be honorably mentioned for his experiments in this direction, and Dr. O'Dwyer would be remembered by posterity as one of the benefactors of mankind.

The first paper read before the section was by Dr. Jules Simon, of Paris, who called attention to

A FORM OF CEREBRAL IRRITATION IN CHILDREN, independent of organic lesion, and not the result of heredity or syphilis, but due to the deplorable tuition of young infants, even those at the breast, who are, in many cases, constantly harassed by the nurse with loud singing and sudden lights, and are liable to be excited by tea, coffee, or spirits, either directly or through the milk of the nurse. Add to these causes the feverish excitement which spreads around the cradle of the infant in modern society, and the result is a condition of cerebral irritation in which the child is unduly agitated by the most trivial causes. Sleep is light and frequently interrupted; exaggerated reflexes produce vomiting, subsultus, and convulsions. The signs of precocity become more painfully evident when the child reaches the age of two or three years. He is in constant motion. The eye is restless and the expression vacant. The mind is alert, but incapable of application.

The cerebral irritation thus manifested may appear in the first months of life, or it may gradually unfold itself at a later period. It terminates toward the age of five years, either by cure or by cerebral sclerosis, epilepsy, or meningitis.

It is the duty of the physician to secure a strict hygiene, with special view to prevent nervous excitement caused by unusual noises, or sights, or stimulating food and ill-advised friendly and social attentions. The open air and residence at the sea-shore or in the country are desirable, and medication, when required, should be by the bromides.

Dr. S. H. Charlton, of Seymour, Indiana, had recognized the condition described by Dr. Simon, and had traced the cause in some cases to malaria and prolonged hot and dry weather. He emphasized the influence of heredity in this class of patients.

A brief communication presented from M. de Saint-Germain was as follows:

"Not being able to attend the congress, I send a note from my surgical experience. What I bring is not a stone for the edifice, not even a pebble, only a grain of sand. But each must do the best he can. Please accept the will for the deed.

"He who invents a surgical operation receives great credit. Recognition, at least, should be given him who substitutes for two operations two simple procedures, less dangerous, quite as effective, and more easy to perform. I propose ignipuncture of the tonsils and preputial dilatation in place of tonsillotomy and circumcision.

"IGNIPUNCTURE OF THE TONSILS.

"Tonsillotomy is not free from the possibility of fatal accidents. To mention uncontrollable hæmorrhage and invasion of the wound by diphtheria is to make it clear that the operation is not so harmless as has been supposed.

"Krishaber tried the thermo-cautery, but his application was so superficial that treatment was indefinitely prolonged.

"I operate with the aid of a modified Smith's gag, thrusting the thermo-cautery into the tonsil to the depth of three-eighths of an inch. Two to four applications, at weekly intervals, reduces the tonsil to a shrivelled and insignificant stump.

"As to

PREPUTIAL DILATATION, it may well take the place of circumcision, which is sometimes followed by serious hæmorrhage, diphtheritic invasion of the wound or partial gangrene.

"I reserve circumcision for those cases alone (about one in three hundred) in which dilatation is impracticable. I used a two-bladed dilator instead of the three blades of Nelaton, introducing it and slowly expanding the orifice. The operation is finished by separating the adhesions with a grooved director and is followed by daily massage, in which the glans is alternately exposed and covered.

"With both of these simple procedures I have always secured excellent and durable results, and have met with no untoward complications. In view of the great frequency of these two classes of cases, am I not right in presenting these simple and effective procedures as a surgical advances?"

Professor Lewis A. Sayre, of New York, then read a paper on

THE DELETERIOUS RESULTS OF NARROW PREPUCE AND PREPUTIAL ADHESIONS.

His first paper on this subject was published in the "Transactions of the American Medical Association." He was the first to draw the attention of the public to this important subject. It is now generally admitted that paralysis, and various other nervous symptoms, including a want of coordinating power, are in some cases induced by the pressure of the prepuce on the glans. The remedy is removal of the constriction and of the retained and concrete smegma, and such an arrangement of the parts that the prepuce shall glide *easily* to and fro over the glans, without restriction, permitting cleanliness, and thus removing one great source of danger.

For this proper arrangement of the prepuce it is necessary in some cases to perform circumcission, or an actual removal of a small portion of the prepuce, and sometimes to dissect it from *actual adhesion* which is a very different thing from ordinary normal agglutination. But there is no occasion for removing any tissue, unless there is great redundancy *with constriction.* And in the great majority of cases the object sought can be easily accomplished by pushing a grooved director as far back as possible, and then dividing with the curved bistoury enough tissue to allow of tearing back the prepuce and uncovering the glans. The next step is to make a slight nick with the scissors, or bistoury, though the thickened fold of the edge of the frænum. Having done this, it is easy with the thumbs and forefingers to tear down the frænum and other adhesions, expose the glans, and remove from the sulcus behind the corona the hardened smegma, sometimes containing chalky concretions. In this procedure there is little loss of blood, and no loss of tissue whatever. A stitch on either side of the incision, between the skin and the mucous membrane, may or may not be required. Thus the glans is left partially covered, and it may as well be *freely* and *easily* uncovered. Having been responsible for bringing the subject before the profession, the writer wished to raise his voice against the mutilation and disfigurement of the organ which is often seen, which by too free removal of the prepuce leaves the glans entirely unprotected, as well as against the indiscriminate performance of the operation in cases where it can be of no avail.

The object of the paper was to harmonize two views—that of those who would operate in cases of infantile paralysis, and that of those who deny the existence of a paralyzed or even muscular inco-ordination from reflex genital irritation—by showing that there are cases of anomalous and extraordinary nervous manifestations certainly dependent on some irritation of the genital organs, in which an operation is not only justifiable, but absolutely demanded, and that in many instances the relief from all the strange symptoms has not only been immediate, but permanent after the operation, without any other medical or surgical treatment. It is also equally certain that any attempt to relieve a nervous disturbance dependent on some central lesion of the brain or spinal cord would result in no benefit whatever. The views of the writer were sustained by a large number of cases occurring in the practice of physicians in different parts of the country.

Dr. De F. Willard expressed his belief in the existence of reflex symptoms from genital irritation, and that many cases can be relieved by uncovering the glans. He advocated exposing the glans by manipulation with the thumbs and forefingers, without incision of the prepuce or frænum, continued and repeated until the prepuce is no longer tight.

Dr. I. N. Love, of St. Louis, Missouri, had for many years practiced circumcision. He believed in the Mosaic law from the standpoint of sanitation, morality, and the general well-being of the child. He had not succeeded in inducing mothers and nurses to secure absolute cleanliness when the sulcus is habitually covered by the prepuce.

Dr. S. C. Gordon, of Portland, Maine, also advocated circumcision and believed that it should be more radically performed than it usually is.

Dr. P. R. Furbeck, of Gloversville, New York, recognized the importance of uncovering the glans and securing easy motion of the prepuce, but believed in the value and importance of dilatation, because many parents refuse to accept the use of the knife. He recalled a case in which a child of six years was relieved from choreic symptoms by dilatation after circumcision had been strongly opposed.

Professor Charles Warrington Earle, of Chicago, Illinois, read a paper entitled

AN INVESTIGATION TO DETERMINE WHETHER THE ABSENCE OF SEWERAGE AND OF WATER-POLLUTION DIMINISH THE PREVALENCE AND SEVERITY OF DIPHTHERIA.

He presented the results of a study of the causes of diphtheria in localities remote from sewer-gas influence in the less thickly populated Western States and Territories. He had received communications from a large number of physicians widely scattered over this great region.

His conclusions are briefly summarized as follows:

1. Diphtheria occurs in the mountains and prairies of the great Northwest with the same malignancy as in the East.

2. And with equal virulence in vicinities remote from sewers.

3. When once introduced, the residents of damp sod houses suffer with marked severity.

4. The infection is transported thousands of miles in some unrecognized vehicle.

5. There is abundant testimony that it follows the lines of railroads and steamers, making it imperative to increase the watchfulness and improve the methods of disinfection by railroad and steamboat companies.

6. The desirability of legal enactments obliging people of all classes to recognize their responsibility in regard to the control of contagious diseases.

Dr. W. Foster, of Putnam, Connecticut, reported the apparent connection between diphtheria and exposure to filth in two cases occurring in a town of 7,000 inhabitants, otherwise entirely free from the disease. The boys affected had been playing almost constantly for several days in and about a barn, the cemented cellar of which received sink-water and house-refuse as well as manure. Isolation and thorough disinfection prevented the spread of the disease.

Professor F. E. Waxham, of Chicago, Illinois, believed that diphtheria is due rather to the absence of sewers than to their presence. An impure atmosphere and the presence of filth and decomposing vegetable matter are important factors. Absolute cleanliness, which, of course, includes disinfection, is our best resort.

---

### SECTION ON DENTAL AND ORAL SURGERY.

Jonathan Taft, M.D., of Cincinnati, Ohio, President.

Secretaries.—A. M. Dudley, M.D., of Salem, Massachusetts; F. H. Rehwinkel, M.D., of Chillicothe, Ohio.

### First Day—Afternoon Session.

Professor Taft, the president, welcomed those present.

Drs. I. V. Metnitz, of Austria; B. McLeod, of Scotland; and Greevers, of Holland, replied in behalf of the countries they represent.

The president then delivered his address, in which he reviewed the progress of dentistry in the last fifty years, and concluded by saying that although the past record was an excellent one, yet the goal is not yet reached. He urged the profession, through those present, to work in all earnest for a yet higher standard.

Dr. R. J. Porre, of Cincinnati, Ohio, read a paper on

CHRONIC PYÆMIA FROM DENTAL ORIGIN.

The history of the first case was as follows: The patient, male, good constitution and habits, suffered for the last thirty years from neuralgia, besides having constantly recurring furuncles and eruptions in various parts of the body, which would often for months become running abscesses. He experienced burning and itching eruptions of hands and feet, which would finally change to stubborn ulcerations. His bowels were either stubbornly constipated or exhaustingly loose. He suffered from frequent rigors and febrile attacks of varying intensity, profuse night-sweats, retention of urine, serious constriction of the bowels and urethra. Lancinating pains darted from the maxilla of right side to bowels, bladder, limbs, hands and feet, or to whatever part that was locally affected at the time. This latter peculiarity, together with the discovery of a little pus exuding from the locality of the wisdom-tooth, led to a final correct diagnosis of his case.

The tooth referred to was extracted, and a speedy and complete recovery followed. As other sources leading to pyæmia and having their starting-point in the oral cavity may be mentioned pyorrhœa alveolaris, alveolar abscess, abscess of the antrum, and dental caries.

Dr. Porre related ten other cases similar to the above, which all yielded to the simple remedy of removing the offending teeth.

Professor J. Frank Lydston, of Chicago, Illinois, said that both physicians and dentists should appreciate the important relation which morbid conditions of the mouth and jaws, and especially those which may be produced by septic absorption, bear to different general conditions. Septic matter is quite generally found about the roots of teeth, and may, under favoring circumstances, be absorbed into the blood, and there produce disturbances of greater or less degree.

The paper was further discussed by Drs. Walker, of London, England; Barrett, of Buffalo, New York; W. J. Younger, of San Francisco, California, and Chance, of Oregon.

### SECTION ON OPHTHALMOLOGY.

J. J. Chisolm, M.D., of Baltimore, Maryland, President.

Secretaries—A. Alt, M.D., of St. Louis, Missouri; J. A. White, M.D., of Richmond, Virginia, and R. L. Randolph, M.D., of Baltimore, Maryland.

### First Day—Afternoon Session.

Professor Chisolm, the President, on taking the chair, expressed his high appreciation of the honor conferred upon him, and referred in feeling terms to the regret felt at the absence of Professor E. Williams, of Cincinnati, the unanimous choice for the position. He referred to Professor Williams as the pioneer in ophthalmology, the first of the specialties to branch out as a separate department of medical science, and the difficulties he had met with when devoting himself to diseases of the eye and ear exclusively. To-day it is acknowledged that progress in general medicine is attained only through studies and researches in special departments, and every organ has its investigator. The assembly of this International Medical Congress is, to a large extent, an assemblage of eminent specialists from great distances, at personal expense, to interchange their thoughts and conclusions upon various subjects. He referred to the vastness of the subject of the human eye alone, and that there is no disease of the eye so thoroughly mastered that further information is not desired. Operations are still far from perfect, and the origin and nature of glaucoma is not clearly defined.

The microbic element in eye affections was also mentioned, and the intimate relation of eye diseases to distant reflexes.

In concluding, he repeated his welcome to the members of the Section as friends and co-workers in the department of ophthalmology.

The first paper of the session was on

EYE TROUBLES IN THEIR RELATION TO OCCIPITAL DISEASE, by Dr. A. Mooren, of Düsseldorf, Germany.

Dr. Mooren introduced his subject by a recital of the initial observations of Huguenin and the experimental researches of Munk, who removed the visual spheres in the occipital lobe of the dog and found that it rendered the animal blind, but left intact all the functions and movements not dependent on vision. Then, after referring to the clinical observations of Hirschberg, Pooley, Pflüger, Hughlings-Jackson, Gowers, and others, he entered into analysis of 42 different cases of his own in which hemianopsia was present, 14 of which were on the right side, 19 on the left side, 4 temporal and 5 nasal restriction. The causes were various.

He found that hemianopsia dependent on occipital lobe disease is not complicated with mydriasis or capillary apoplexies on the insertion of the optic nerve. It is the merit of Willbrand to have collected the different observations made in this sense. He formulates as follows:

The color-sense is to be placed on the most exterior cortex of the occipital lobe; beneath this another layer, the center for acuteness of vision. In a third layer beneath this again there exists next to Gratiolet's visual radiations, the lightcenter, *i. e.*, the center for visual field. Destruction of the upper or color-perception layer may take place without affecting the underlying layers, as is demonstrated by the observations of Bjernum and Samelsohn, and Dr. Mooren's observations of the loss of color-perception in his cases without necessary impairment of vision, are in accordance with those of previous observers.

He referred to the determination by Nothnagel of the occipital limits of pathological changes, by means of the method of the smaller focus. After the observations of Haab, Huguenin, Feré and Seguin, this neurologist places the center of optic perception in the cortex of the cuneus and first occipital convolution. This is confirmed by sections made by Curschmann.

Dr. H. Gradle, of Chicago, Illinois, mentioned a case which appeared to him to have a bearing on the subject under discussion. A child, during an attack of what was supposed to be scarlet fever, had pain in the back of the head, which was drawn backward. Some time afterward, when seen by the narrator, it appeared to be blind, but the pupils were both quite active to light. Treatment by potassium iodide appeared to produce a beneficial result, and when last seen vision seemed to be completely restored.

The President presented in this connection a case of blindness with occipital disease. Some years ago pain began to be felt behind the left ear, shortly followed by swelling in the same region. The swelling gradually increased, and now a softish tumor of considerable size can be seen and felt, over which the bone has been absorbed. In the later period of its growth the hearing in the left ear became dull, and the right eye became blind. He could still read with the left eye. At that time both optic nerves appeared white and atrophic, one not more than the other. The pain has now almost disappeared, but he has only perception of light in the left eye.

The discussion was also participated in by Dr. Dickinson, of Missouri, Dr. Beerman, and others.

Dr. Ole Bull, of Christiania, Sweden, read a paper entitled

PATHOLOGICAL CHANGES IN THE RETINAL VESSELS.

He had seen eighteen cases of disease of the retinal vessels in six thousand five hundred cases of eye disease. In some there were emboli, in others what appeared to be constriction or narrowing of the retinal vessels. He noted in some of the cases occasional spasmodic constriction of the arteries, with resulting anæmia and loss of vision, with some recoveries of vision, as distinguished from true emboli. Thrombus and gumma were referred to as causes of pathological changes in the vessels, in some cases distention, in others constriction, resulting. In all cases of organic change, serious impairment of vision or total blindness resulted.

Professor P. D. Keyser, of Philadelphia, questioned as to whether in many of the cases it was a true embolism or a contraction of the arteries. A case which came to him blind from what appeared to be embolism, recovered good central vision, but had a field only two inches square. The treatment was galvanism. He had questioned whether, if the treatment had been commenced earlier, the vision and field might not have been entirely restored.

Dr. A. Heyl, of Philadelphia, Pennsylvania, questioned as to whether it was embolism or a loss of blood-tension, as in a case of blindness after post-partum hæmorrhage, where the blood-amount is much lessened and tension much diminished. He also believed that one element in the blood-tension is the contractile force of the endothelium of the blood-vessel. When the tension is diminished this force comes into play, especially in the retinal artery as a terminal vessel without anastomosis. He cited a case of sudden loss of vision in a young lady with no heart or kidney disease, not especially anæmic, but of a somewhat waxy cast of complexion. It looked like embolism. Treatment was by palpation or percussion over the frontal and temporal adjacent region, by means of a rubber ball on a stick, and had seemed to be beneficial. He also thought that acute glaucoma might be a congestion caused by a sudden disturbance of the same nature in the circulatory system.

Dr. Bull closed the discussion by expressing his belief that many of these troubles are due to trophic affections, with no disease or affection of any organ elsewhere.

The paper by Professor E. Smith, of Detroit, Michigan, on "Treatment of Abscesses and Ulcerations of the Cornea by Jequirity," was postponed till a later session by unanimous consent.

Dr. Leartus Connor, of Detroit, Michigan, read a paper entitled

HOT WATER IN THE TREATMENT OF EYE-DISEASES.

Dr. Connor stated that that remedy was sought after which would most certainly induce, first, good feeding of the tissue; second, removal of morbid products and morbific agents; and third, the promotion of speedy repair. Such an agent is hot water in a great variety of eye affections such as mild catarrhal and phlyctenular conjunctivitis, corneitis, affections of the sclera and iris, and even, in some cases, retinal hyperæmia. In iritis, where the pupil refuses to respond to mydriatics, hot water will exert a marked effect in assisting dilatation of the pupil. Similar beneficial results in reducing inflammatory action had been noticed by him in catarrhal and purulent ophthalmia, in relieving the pain in glaucoma and acute dacryocystitis.

There is no morbid state of the eye on which it may not exert beneficial influence. The results reported by divers observers vary with the different modes of using it. The water should be as hot as the end of the forefinger will bear without discomfort. The method preferred by the essayist was to take a common tumbler, fill it to the brim with hot water, and inclining the head slightly forward, apply the rim of the tumbler to the side of the nose and to the brow and cheek about the eye, which brings the eye itself actually into the water. The amount of water loses its heat slowly, and does not require frequent changing, and the eye may be kept in hot water with very little trouble for hours at a time, Antiseptics may be added, and the remedy is easily attainable with means for application. It is safe without the watchful care of the physician, while moist heat by any solid substance, as poultices, should never be used except under the direct supervision of the attendant.

Poultices are unsafe and unreliable means of applying heat to the eye; also dirty, especially on denuded surfaces. Compresses are less objectionable, and may be used as a substitute for hot water.

Local effects: 1. Contraction of blood vessels in and about the eye. Controls hæmorrhages better than cold water and blanches the tissues in conjunctivitis, blepharitis, phlyctenulæ; and after the use of hot water the ophthalmoscope shows the retinal vessels to be reduced in size. In one case where drawings were made of the vessels before and after, the difference was very marked, and the relief of retinal congestion and improvement of vision very noticeable.

The temperature of the water must vary with the sensation of the patient. The tissues should not be exhausted.

2. Hot water will wash away or destroy all morbific secretions or excretions. At a temperature of 132° F. it destroys the bacillus of anthrax and many others, and many eyes can bear a somewhat higher temperature.

3. It promotes the healthful activity of reparative tissue or protoplasm.

4. It exerts direct power in relieving muscular fatigue and spasm.

Professor Dudley S. Reynolds, of Louisville, Kentucky, asked whether the reader of the paper used hot water, as he had stated, immediately after a strabismus-operation, to increase the effect by causing contraction of the cut muscle.

In reply it was stated that contraction was stimulated in the muscle antagonistic to the cut muscle, and the effect increased in that way.

Dr. J. L. Thompson, of Indianapolis, Indiana, defended the poultice as used in affections of the anterior part of the uveal tract and sclera. He had had excoriations produced by hot water, and then had to use poultices. Chamomile or slippery elm, in cheese cloth, was with him the preferable form.

Professor P. D. Keyser, of Philadelphia, Pennsylvania, spoke of DeWecker's use of hot water a long time ago in conjunctivitis diphtheritica, as he said, to soften the tissues and hasten absorption. The speaker had found diphtheritic conjunctivitis rare in this country, but membrane on the conjunctiva not so uncommon. He had used hot compresses over the eyes, and kept them hot by spraying with steam directed on the compresses from an ordinary kettle by a forked tube. The compresses should be applied over the eyes alone, and not over the frontal sinus between the eyes.

Dr. H. Power, of London, England, was in favor of dry poultices rather than moist ones. He has chamomile flowers or hops heated in a dry kettle, stirred till hot through, then applied in a bag or pillow to the part affected. He objects to the use of the steam from the kettle.

Professor Eugene Smith, of Detroit, Michigan, fully agreed with Dr. Power. He thinks moist heat not advisable in ulcerative keratitis.

Dr. P. T. Huckins, of Los Angeles, California, believes in the use of heat in these affections, but finds that the length of time during which the heat should be applied has not been spoken of.

Dr. A. Blitz, of Minneapolis, Minnesota, has never seen bad results, in all cases of conjunctival or uveal inflammations, from the use of hot water.

Dr. Abadie, of Paris, France, thinks that not so much stress is now laid upon the employment of hot water in many eye troubles. It has gone out of use in phlyctenular conjunctivitis, and purulent and croupous ophthalmia. In corneal affections, internal medication and proper antiseptic applications are far superior. In infectious corneal ulcers, he uses iodoform powder and antiseptic washings. On the other hand, in iritis with much pain hot applications are good, but morphia, leeches, and internal administration of quinia sulphate, salicylate of soda, etc., are often all that is necessary.

Dr. Herbert, of Philadelphia, Pennsylvania, wished to call attention to a mode of applying heat that had not been mentioned, that by means of irrigation; the hot water flowing over the inflamed surface was thus kept at a constant temperature.

Professor A. W. Calhoun, of Atlanta, Georgia, spoke of an apparatus which he had sometimes used, a thin rubber bag of proper size filled with hot water and applied to the eye. It could have an inlet pipe to supply fresh hot water, and an outlet to carry away the cooler portions.

Professor Hotz, of Chicago, Illinois, remarked that the diversity of modes of application of heat, with such uniformly good results, tends to show that there must be a principle underlying the mere material, or the manner in which it is applied. He should lay down the principle, therefore, to apply heat where there is a stagnation of blood to be broken, and it will reduce pain and promote recovery. The feeling of comfort that the patient experiences is the best gauge by which to tell the proper temperature in each particular case.

### SECTION ON PUBLIC AND INTERNATIONAL HYGIENE.

Joseph Jones, M.D., New Orleans, Louisiana, President.

Secretaries.—Dr. Castallanos, Dr. Felix Formento, and Dr. J. R. Le Monnier, of New Orleans, Louisiana; Dr. B D. Taylor, of Columbus, Ohio, and Dr. Walter Wyman, U.S. Marine Hospital service.

FIRST DAY—AFTERNOON SESSION.

Professor Jones, the President, delivered an address on the general subject of hygiene, which was the only subject considered, other than reports of committees.

He considered the question under three heads —domestic, national, and international hygiene. The first related to families and households; the second included the organization of boards of health and sanitary administration; the third, vital statistics and race distinctions.

### GENERAL SESSION.

SECOND DAY—TUESDAY, SEPTEMBER 6TH.

The congress was called to order at 10 A.M. by the president.

Professor Austin Flint, M.D., LL.D., of New York, delivered a general address on

### FEVER, ITS CAUSES, MECHANISM AND RATIONAL TREATMENT.

After discussing the subject of animal heat, the following conclusions were reached by the author:

1. Fevers, especially those belonging to the class of acute diseases, are self-limited in their duration, and are due each one to a special cause, a micro-organism, the operation of which ceases after the lapse of a certain time.

2. We are as yet unable to destroy directly the morbific organisms which give rise to continued fevers; and we must be content, for the present, to moderate their action and to sustain the powers of resistance of patients.

3. The production of animal heat involves oxidation of parts of the organism or of articles of food, represented in the formation and discharge of nitrogenized excrementitious matters, carbonic acid and water.

4. As regards its relations to general nutrition and the production of animal heat, water formed in the body by a process of oxidation is to be counted as an excrementitious principle.

5. Fever, as observed in the so-called essential fever, may be defined as a condition of excessive production of heat, involving defective nutrition or inanition, an excessive production and discharge of nitrogenized excrementitious matters and carbonic acid, with waste and degeneration of the tissues, and partial or complete suppression of the production and discharge of water.

6. Aside from the influence of complications and accidents, the ataxic symptoms in fevers, the intensity and persistence of which endanger life, are secondary to the fever, and are usually proportionate to the elevation of temperature. These symptoms are ameliorated by measures of treatment directed to a reduction of the general temperature of the body.

7. The abstraction of heat by external cold and the reduction of temperature by antipyretics administered internally, without affecting the special cause of the fever, improve the symptoms which are secondary to the pyrexia.

8. In health, during a period of inanition, the consumption of the tissues in the production of animal heat is in a measure saved by an increased production and excretion of water.

9. In fever, the effects of inanition, manifested by destruction and degeneration of tissues, are intensified by a deficient formation and excretion of water.

10. Alimentation in fever, the object of which is to retard and repair the destruction and degeneration of tissues and organs, is difficult mainly on account of derangements of the digestive organs; and this difficulty is to be met by the administration of articles of food easily digested, or of articles in which the processes of digestion have been begun or are partly accomplished.

11. In the introduction of hydrocarbons, which are important factors in the production of animal heat, alcohol presents a form of hydrocarbon which is promptly oxidized, and in which absorption can take place without preparation by digestion.

12. Precisely in so far as it is oxidized in the body, alcohol furnishes matter which is consumed in the excessive production of heat in fever, and saves destruction and degeneration of tissue.

13. The introduction of matters consumed in the production of heat in fever diminishes, rather than increases, the intensity of the pyrexia.

14. As the oxidation of alcohol necessarily involves the formation of water and limits the destruction of tissue, its action in fever tends to restore the normal processes of heat-production, in which the formation of water plays an important part.

15. The great objects in the treatment of fever itself are to limit and reduce the pyrexia by direct and indirect means; to limit and repair destruction and degeneration of tissues and organs by alimentation; to provide matters for consumption in the abnormal production of heat, and thus to place the system in the most favorable condition for recuperation after the disease shall have run its course.

---

### SECTION ON ANATOMY.

### Second Day—Morning Session.

The first paper read was on

THE ANATOMY AND SURGICAL IMPORTANCE OF THE PERI-RENAL CELLULO-ADIPOSE TISSUE AND RENAL CAPSULE, by L. H. Dunning, M.D., South Bend, Indiana. After outlining the position of the kidney and its anatomical relations, he then called attention to a double form of the peri-renal cellulo-adipose tissue, which, he thought, was a wise provision of nature and of great surgical importance. He explained the anatomy of the renal capsule, the arrangement of the fat, and the position of the cellular tissue, thus preventing the stretching of the nerves and the accompanying pain. In speaking of the capsule, he thought it acted as a barrier to prevent inflammation to and from the kidney, and also furnished a surface for the attachment of the peri-renal cellulo-adipose tissue. The surgical importance of this peri-renal cellulo-adipose tissue and of the renal capsule lay in the fact that one or both were often subject to inflammation and morbid changes, and that they were often invaded by the surgeon. Tumors often occurred within or upon the fibrous cellulo-adipose tissue of the kidney. The question was now, if there were real danger attendant upon these invasions into the renal territory. In the three most frequent operations upon the kidney—nephrorrhaphy, nephro-lithotomy, and nephrectomy—the peri-renal cellulo-adispose tissue was cut through. The adipose and fibrous tissue, which was very vascular in this region, was very liable to inflammations. In operating, all loose portions of fat should be removed and the fibrous capsule, or even a small portion of the cortical portion of the kidney, might be left. The author asked also what should be done with the cellulo-adipose structure and the fibrous capsule left behind. His supposition, from the careful observation of several cases and a second operation on one, that after removal of the kidney the cavity was partially collapsed and filled with granulations, and this scar-tissue took the place of the kidney. These fibrous bands continued to contract, displacing the surrounding tissue. His final opinion was to remove all tissue when it was not too vascular.

The President spoke of the important point mentioned by Dr. Dunning, in regard to the close connection between the movements of the diaphragm and of the abdominal organs. He was of the opinion that the whole kidney could be destroyed and the capsule be left.

Dr. W. T. Oppenheimer, of Richmond, Virginia, then exhibited several photographs of

AN APPARATUS FOR THE TREATMENT OF FRACTURES OF THE SURGICAL AND ANATOMICAL NECK OF THE HUMERUS.

He spoke of the great difficulty in keeping the fractured extremities of the bone in apposition, and thought that his apparatus covered the three necessities of extension, counter-extension, and fixation. The apparatus consisted of strong iron bands, which were fastened around the thorax by means of plaster of paris, and which extended to the fractured arm, holding it firmly at the shoulder, upper arm, and forearm. He had used it in several severe cases of fracture of the neck of the humerus, and suggested using it in fracture of the clavicle and in excision of the elbow joint.

The President asked when passive motion was commenced.

Dr. Thomas, of Pittsburg, Pennsylvania, thought it did not matter what apparatus was used, just so the arm was held out from the body. He admitted, however, that he had never treated a case in that way, but thought it formed the principle of Dr. Oppenheimer's apparatus.

Dr. Oppenheimer replied that in about five weeks passive motion was commenced. He said when the plaster jacket was put on well, and the bands were strong, that no movement of the body could cause crepitation.

The President asked if a corset of leather or of other material could not be used instead of the plaster of paris, which, in his opinion, seemed a little clumsy for such treatment. He also thought that in using crepitus there was danger of tearing the periosteum, and thus cutting off the blood-supply of the fractured ends.

### Afternoon Session.

Professor Pancoast, the president, after greeting the members of the section, began by saying that anatomy was sometimes called a dead study, but he thought it was a live subject. He thought that many advances had been made since the last meeting of the congress, and especially in the anatomy of the brain. Great progress had also been made in microscopical anatomy. The study of the electrical sensibility of the brain and the operations for the cure of epilepsy by Hughlings Jackson, Ferrier, and others had proved itself to be very successful. The ability to localize brain-troubles by the part paralyzed was evidence of great practical advances in the study of brain-surgery. He then related a very interesting case of his own of a gun-shot wound of the brain which he had successfully treated by aid of a careful study of the symptoms and the use of the induction balance. He did not think that anatomy made it sufficiently distinct that all muscles from one bone to the bone below were articular muscles. This was shown in a paralysis of the deltoid muscle making a simulated dislocation. He defined a joint at that division of the skeleton made to deaden shock and cause motion. He spoke of the low vitality of the joint-cartilage and the snowy-white membrane observed on the articular cartilage in white swelling. He did not altogether approve of extension and counter extension in diseased joint, and thought that rest was important and that it was sufficient to prevent the rubbing together of the diseased surfaces. He believed that there were only four metacarpal bones in each hand, and referred to the development of the hand and foot as a proof. He mentioned several anomalies of muscles which had come under his observation, such as two heads to the latissimus dorsi, and particularly of an unequal development of the soleus and gastrocnemius, wherein the latter muscle was too long. In certain forms of talipes with this muscular condition a cutting of the tendo Achillis to effect a cure. The tense soleus had to be alone divided by slipping the knife between the two muscles and cutting the soleus. He thought that the deep fasciæ were not only for protection but acted as ligaments. He mentioned fractures of the coronoid process of the ulna and their causes, and was of the opinion that muscular contraction of the brachialis anticus could not break the coronoid process because the latter was covered by the anterior ligament of the joint, He thought the word anastomosis as applied to nerves and tissue incorrect, and should only be used when speaking of vessels. He explained the direction of the nutrient arteries in the long bones of the extremities and the peculiar position of the fœtus in utero causing their direction, and the importance of understanding the entrance of these nutrient vessels into the bones in some fractures. He thought many names might be simplified, and spoke of the recent study of the skull and spinal column. He concluded by quoting the authorities on the advances made in the study of anatomy in different parts of the world.

Dr. Albert B. Strong, of Chicago, Illinois, then read a paper on

FROZEN SECTIONS OF THE MALE PELVIS, SHOWING RELATIONS OF PERITONEUM TO RECTUM, BLADDER AND THE MEDIAN LINE OF ABDOMINAL WALL, WITH SPECIAL REFERENCE TO SUPRAPUBIC CYSTOTOMY.

He exhibited some very well-executed reproductions of photographs of elaborately prepared specimens. He found air better than water to lift the bladde rand its peritoneal reflection out of the pelvis. He found that distending the rectum first and the bladder afterward, and moderately distending both, met the indications the best.

Dr. Gervais, of Belgium, always punctured the bladder when the patient was standing and the bladder filled.

Professor F. C. Schaefer, of Chicago, Illinois, then read a paper entitled

ANATOMICAL POINTS INVOLVED IN THE LOSS OF COMPLETE SCALP, INCLUDING ONE EAR AND THE GREATER PORTION OF THE EYELIDS.

This paper was founded upon a case in which a girl in a factory was caught by the hair and scalped. He had not been able to find many such cases on record, but was of the opinion that the results heretofore obtained had not been very successful. He at first attempted, against his own judgment, to replace the scalp, but was unsuccessful; he then began skin-grafting, with which he had obtained very satisfactory results, which his excellent photographs of the case proved. He was of the opinion that small grafts placed close together, were better than large ones; that grafts with the subcutaneous tissue were the best kind; that auto-dermal grafts were better than dermal grafts; that grafts from a person near the same age as the patient were more successful. He thought that the grafts so placed were probably nourished by osmosis. He found that grafts in a new wound grew much faster than those in an old one.

Dr. J. A. Murphy, of Chicago, Illinois, emphasized the importance of taking dermal grafts with plenty of subcutaneous tissue.

The President thought that grafts taken from the scalp itself might have grown successfully.

Professor Schaefer, in closing, said that in his case the machinery had so mangled the scalp that it was entirely bloodless when examined. He also drew the distinction between skin-grafting and a plastic operation, which was, of course, an entirely different thing.

---

### SECTION ON DENTAL AND ORAL SURGERY.

### SECOND DAY—MORNING SESSION.

Dr. William Carr, New York, N. Y., gave a clinic on the

#### TREATMENT OF FRACTURES OF THE MAXILLÆ WITH MODIFIED INTERDENTAL SPLINT.

The majority of fractures of the inferior maxilla occur in the body rarely at the symphysis menti, but usually directly anterior or posterior to the mental foramen. A noticeable fact in connection with these fractures is that the victim rarely applies for treatment for several days succeeding the injury. He realizes that some of his teeth are loosened and also that he is painfully bruised, but does not seek surgical aid until he becomes alarmed by the increased inflammatory condition of the parts. There is but little difficulty in establishing a correct diagnosis, as usually the following symptoms are present—great pain in the effort to open and close the mouth, swelling, crepitus, inflammation, inability to masticate, and marked irregularity of the teeth.

Treatment.—It is identical with that of other fractures, namely, to bring the parts into apposition and retain them firmly until ossification is completed. For treatment of fractures of the maxillæ there is nothing superior to the interdental splint. When properly adjusted, speedy union may be secured without deformity of the jaw or irregularity of the teeth. Before taking the impression a careful examination of the parts should be made. Loose teeth and spicula of bone should be removed, and the parts should then be brought as nearly as possible to their normal position. An accurate impression should be made with impression compound or wax. The material used should be as warm as the patient can bear it, in order to prevent unnecessary pain, and also to prevent further displacement of the parts. The splint is made of vulcanite and covers all the teeth of the lower jaw, and all the teeth posterior to the canine in the upper jaw—leaving a space of about three or four lines through which the patient may receive nourishment. Small holes are drilled in the splint over the grinding surface of each molar for the purpose of ascertaining whether its adjustment is proper.

The splint should first be adjusted to the sound jaw, then gently bring the fractured jaw into position until it has passed about two-thirds of the length of the teeth—then with a quick, firm motion bring the parts into position. Next apply a four-tail bandage, which should be retained from three to five days; after this time, in the majority of cases, it may with safety be removed during the day, but should be replaced at night until the removal of the splint. The patient should be furnished with an ordinary rubber syringe, and instructed to keep the mouth thoroughly cleansed. For disinfectants I use peroxide of hydrogen, three per cent. solution, or a solution of bisulphate of soda in the proportion of ʒ j to ℥ j, of water.

In ordinary cases the splint should be retained for three or four weeks, according to the physical condition of the patient—unless unforeseen complications should arise. The application of the splint, combined with thorough cleanliness, will usually be all the treatment required.

The advantages, besides those previously stated, are that the patient experiences but little pain and inconvenience, and can, as a rule, attend to his business almost immediately after the splint is applied.

It is not necessary that all the teeth, nor, indeed, that any should be present in the mouth in order to make this splint serve its purpose. In the first case the rubber can be made to take the place of the missing teeth, and in the latter case a perfect adaptation of the splint to the alveolar ridges can be secured, and will be found to keep the parts in perfect apposition.

Should it be deemed advisable to place a splint in position within an hour or two after seeing the case, one can be constructed entirely of ordinary gutta-percha, with just enough wire inside to stiffen it. Dr. Carr demonstrated this last method—it is very simple and can be made by any surgeon.

A number of gentlemen examined the principle and pronounced it very satisfactory in every way, the main points being its simplicity of construction, its effectiveness, and the ease with which it is adjusted and worn by the patient.

Dr. E. Brasseur, of Paris, France, read a paper on

#### THE USE OF AIR IN DENTAL THERAPEUTICS.

The reader urged that the ordinary means, such as bichloride and biniodide of mercury and carbolic-acid crystals, for destroying microbes in the oral cavity and, especially, in carious cavities of teeth should be supplemented by the use of hot air.

Dr. C. A. Brackett, of Newport, Rhode Island, discussed the paper at some length, laying considerable stress on the efficacy of crystallized carbolic acid as a germicide in carious cavities in teeth,

Other discussions followed, by Professors James Truman and W. H. Morgan.

AFTERNOON SESSION.

Dr. Junius E. Cravens, of Indianapolis, Indiana, read a paper on on

THE MANAGEMENT OF PULPLESS TEETH.

This system is based on the proposition that a pulpless tooth is not necessarily dead. The pulp being devitalized, the tooth still retains life through its pericementum. The usual course of treating pulpless teeth with escarotics and irritants causes irritation and final destruction of the pericementum, and the result is that the tooth, instead of being preserved, will act as a foreign body, and will be thrown off by nature through abscesses; or, worse still, will lead to no end of nervous derangements.

The treatment suggested by the reader is to thoroughly cleanse the pulp-canal, and at once hermetically seal it with tin-foil.

The paper was discussed by Professor Thomas Fillebrown, of Portland, Maine. He did not agree with the essayist in the method outlined in the paper. The doctor gave a short synopsis of the method he employs in treating pulpless teeth, which, by the manner in which it was received by the section, seemed to be the one generally pursued.

Professor A. W. Harlan, of Chicago, Illinois, followed, and likewise objected to the views expressed by the essayist. A dead pulp produces no irritation in the canal; the disease which it causes is beyond. If you could mechanically displace an odor—which the speaker denied—and should then fill the root canal without any disinfection, disaster would inevitably follow unless there should be a fistulous outlet.

Dr. W. C. Barrett, of Buffalo, New York, in discussing the paper, stated whether viewed from the standpoint of pathology or etymology the paper is alike remarkable. That such a mass of absurdities could be presented at a meeting of the world's representatives in dentistry is to me astounding, and I protest against its acceptance as the standard by which to judge the intelligence of American dentists. Why the exploded dogmas of twenty-five years since should be gravely and in all sincerity presented at such a meeting as this, is, I must confess, something for which I was not prepared. The assertion that a closed chamber in which exists the septic débris and the products of decomposition of a tooth-pulp should not be opened and evacuated, I can scarcely bebelieve is made in calm earnest. The essayist has exhibited his complete ignorance of the progress of the past century.

Modern antiseptic pathology has taught us certain facts, and among these is the knowledge that the first step in the treatment of aseptic cavities is complete drainage; second, disinfection and the removal of all the products of disorganization; third, destruction of septic organisms; and finally, the complete sealing of the cavity against further infection. These comprise the essential steps in the treatment of septic root-canals. I will not insult the intelligence of those present by presuming to enlarge upon this and by going into the details of treatment, for this is not a body of tyros. But I do object to a consideration of the subject from the low standpoint of this extraordinary paper.

Professor T. E. Weeks, of Minneapolis, Minnesota, read a paper on

MATRICES AS ADJUNCTS IN FILLING TEETH.

The essayist reviewed the different appliances for simplifying what would otherwise be very laborious operations. A perfect matrix should be simple in construction, cheap, easily adapted, and not too stiff, so that when applied it will yield just enough to allow sufficient gold to pass beyond the walls of the cavity for a good finish.

Professor F. H. Guilford, of Philadelphia, Pennsylvania, in a few brief remarks, indorsed the sentiment expressed in the paper.

---

### SECTION ON CLIMATOLOGY AND DEMOGRAPHY.

### Second Day.

The section met at 11 A.M. The following resolutions, submitted by the president of the section, were discussed, and, after verbal amendments, unanimously adopted:

*Resolved*, That in the opinion of the section on Medical Climatology and Demography of the Ninth International Medical Congress, assembled in the city of Washington, September 5–10, 1887, it is important there should be established in every country a national department, bureau, or commission for the record of vital statistics upon a uniform basis, to include not only accurate returns of births and deaths, but the results of collective investigation by government officials of facts bearing upon the natural history of disease as manifested among men, women, and children separately, especially with regard to climatic and other discoverable causes of the several forms of disease—race, occupation, and residence being made matters of record—that necessary preventive measures may be determined and enforced for the preservation of the public health.

*Resolved*, That the Secretary-General be requested to have the expression of opinion communicated to the several governments.

Professor Charles Denison, of Denver, Colorado, read a paper on

THE PREFERABLE CLIMATE FOR PHTHISIS.

The paper was elaborately illustrated by maps, diagrams, and tables. Professor Denison believes that the climate to be preferred for the great majority of consumptives in the United States varies from between fifteen hundred feet elevation in the north in winter to ten thousand feet in the southern portion in summer. Certain contraindications exist against sending consumptive patients to high altitudes. The most prominent of these are advanced age of the individual; an excitable, nervous temperament; valvular lesions, with rapid action of the heart; marked and extensive emphysema; pneumothorax and hydro-pneumothorax; active pneumonia or hæmoptysis; high bodily temperature; extensive involvement of lung-tissue, and similar conditions.

He takes the affirmative side of the following five divisions named in the order of their relative importance: (1) Dryness as opposed to moisture; (2) coolness or cold preferable to warmth or heat; (3) rarefaction as opposed to sea-level pressure; (4) sunshine as opposed to cloudiness; (5) variability of temperature as opposed to equability.

Dr. John William Moore, of Dublin, Ireland, then read a paper on

THE SEASONAL PREVALENCE OF PNEUMONIA.

The conclusions—pneumonia has claims to consideration as a specific fever on the following grounds:

1. Its not infrequent epidemic prevalence, which is beyond dispute.

2. Its proved infectiveness.

3. Its occasional pathogenic origin in many cases.

4. Its mode of onset or "invasion," which exactly resembles that of the recognized specific fevers.

5. The appearance of constitutional symptoms before the development of local signs or symptoms.

6. The critical termination of the febrile movement in uncomplicated cases.

7. The presence of local epiphenomena in connection with the skin, as herpes, taches bleuâtres, and desquamation.

8. The development of sequelæ in some cases, such as nephritis, followed by renal dropsy and other conditions.

9. The discovery of a probable pathogenic bacillus, to which analogy points as pathognomonic.

Dr. Moore concludes his paper in these words: "The day is seemingly not far distant when we shall speak of pneumonic fever in precisely the same way as we use the term enteric fever at present; that is, to sygnify a zymotic or specific blood-disease, manifesting itself after the lapse of a certain time—the period of incubation—by physical phenomena, objective and subjective, connected in this instance with the lungs."

THE RELATIONS OF CERTAIN METEOROLOGICAL CONDITIONS TO ACUTE DISEASES OF THE LUNGS AND AIR PASSAGES.

Dr. Henry B. Baker, of Lansing, Michigan, read a paper on the above subject, which was illustrated with diagrams that showed curves for influenza, tonsilitis, croup, bronchitis, and pneumonia, which follow the curve for atmospheric temperature with surprising closeness.

He suggests that the explanation of the causation of these diseases has not been grasped before, because one of the principal facts has not been apprehended, namely, the fact that cold air is always dry air; on the contrary, it has been generally stated that when these diseases occur the air is cold and damp. He explains that while the cold air is damp relatively, it is always absolutely dry, and he thinks that its bad effects on the air-passages are mainly through its drying effects, which can best be appreciated by reflecting that each cubic foot of air inhaled at the temperature of zero, Fahr., can contain only one-half grain of vapor, while when exhaled it is nearly saturated at a temperature of about 98° F., and therefore contains about eighteen and one-half grains of vapor, about eighteen grains of which have been abstracted from the air-passages. Thus cold air, falling upon susceptible surfaces, tends to produce an abnormal dryness which may be followed by irritation and suppuration. He claims that coryza is sometimes so caused. Under some conditions the nasal surfaces are not susceptible to drying, the fluids being supplied in increased quantity to meet the increased demand made by the inhalation of cold air. In that case an unusual evaporation of the fluid leaves behind an unusual quantity of non-volatile salts of the blood, such as sodium chloride, and an unusual irritation results; he thinks influenza is the name commonly given to this condition.

The effects which the inhalation of cold air have on the bronchial surfaces depend greatly upon how the upper air passages have responded to the increased demand for fluids; because if they do not supply the moisture, it must be supplied by the bronchial surfaces; in which case bronchitis results. Finally, if the demands for moisture made by cold air are not met until the air cells are reached, pneumonia is produced.

He refers to statistics which he has published, showing that even the rise and fall of such contagious diseases as scarlet fever, diphtheria, and small-pox follow the same laws shown to control in the acute diseases of the air passages, and he offers the explanation that the irritations and exudations in the air passages caused by the inhalation of cold, dry air supply a nidus for the contagia, and are thus the predisposing causes of those diseases. As to whether or not pneumonia is a contagious disease he offers no evidence except that nearly all of the phenomena seem to be accounted for without the necessity of supposing a special contagium. For the abnormal accumulation of the non-volatile salts of the blood through evaporation of the fluids in the air-cells, so as to cause inflammation and exudation, time is required; therefore, de does not believe that a sudden and short exposure to cold can ordinarily produce pneumonia, except the short exposure follow or precede somewhat prolonged inhalation of cold dry air; although he thinks that lobar pneumonia may have just that causation, the reason for the chill and for the limitation of the area of the exudation being the disturbance of the nervous equilibrium associated with the more or less complete paralysis of the small blood vessels in that part of the lungs supplied by one particular nerve, some or all the endings of reflexions of which have been suddenly exposed to the enervating influence of warmth following the exposure to cold.

---

### SECTION ON MATERIA MEDICA AND THERAPEUTICS.

### SECOND DAY.

Dr. Hugh Hamilton, of Harrisburg, Pennsylvania, read a paper on

#### THE CHEMICAL PHILOSOPHY OF REMEDY.

Activity in bacteriology and advances in chemistry suggested that there is a chemical philosophy in remedy. Doubtless germs are active in altering, by fermentation, the normal organic constituents of the blood into noxious ones not infrequently fatal (Jaksch, 1887): "To assist in clearing the system of this life activity or aid in rapidly removing these effete bodies constitute the aim of remedy; so that *remedy* might be defined as the use of means to restore the body to healthy condition by prophylaxis—repair of in jury and thê correction of nutrition."

The success of antiseptic surgery shows the effects of the application of this principle to diseased conditions. "Germs contain albumen; so if subjected to the physical effects of vacuum, freezing, boiling, or incineration, suffer or perish. Chemically, they succumb to the use of mineral acids, alkalies, certain salts, and organic radicals: "In a word, the deprivation of oxygen, either directly by oxidation of another substance capable of attracting and retaining it, by the loss of hydrogen, by the subtraction or substitution of elemental or approximate radicals. Consequently we can *exclude*, *arrest development*, or totally *destroy* the bacteria." The ptomaines engendered by bacteria are divided into several classes, and upon subsequent elemental analysis show that they contain certain homologues of organic radicals (Cornil and Babes). The application of disinfectants suggested antiseptics, and leads us to anticipate their modified use in internal medicine.

Clinical experience shows that remedies, although often empirically selected, are those containing efficient oxidizers active appropriators of hydrogen, or by substitution of radicals succeed in destroying the pernicious products of germs, its spores, or the consequences of its mere existence in the vital fluid.

The paper was illustrated by diagrams and charts.

Dr. William Murrell, of London, England, said that the subject was one of very great interest. The philosophy of remedy was very generally passed over by the workers in therapeutics. The only ones who appeared to have done much in this field were Professor Wormley, of Philadelphia, and Dr. Stockman, of Edinburgh, who had been doing very valuable work in this branch.

Dr. Ralph Stockman said that the work referred to had been carried on in connection with a friend, he having assisted in matters of technique and experimentation. He had been struck with the paucity of information contained in textbooks, especially with regard to the causation of fever and emaciation. Most writers attribute these to the breaking up of the tissues under the effect of the disease. Dr. Philip, of Edinburgh, has recently made a very interesting investigation into this condition. He found that by taking the sputum of a phthisical subject, protecting it with great care from contamination by external agents, and maintaining it for a short time at the temperature of the body, that an alkaloid was obtainable by Stas' process, which was evidently an alkaloid, a ptomaine. This substance was also detected in the cavities of phthisical lungs. When this substance was injected into mammals it produced fever and progressive emaciation, which proceeded to a fatal termination, even where the injections were discontinued. In frogs the same results were obtained, except that recovery sometimes followed. It was also found that if belladonna and atropine were administered after the new alkaloid had been injected, that the pulse regained its strength, emaciation and fever were checked, and the animal recovered. This is a corroboration of the clinical value of atropine in the treatment of phthisis, to which Bartholow called attention some years ago.

Dr. Coghill was impressed with the value of this work. In confirmation of the remarks of the last speaker, he said that the dose of atropine which could be well borne in phthisis was very much larger than in health. This seemed to warrant the opinion that there was some counteracting agent present in the economy in phthisis which was not present in health.

Dr. J. S. Sinclair Coghill, of Ventnor, Isle of Wight, England, read a paper on

CHLORATE OF POTASH.

After a historical résumé of the introduction of this agent into modern therapeutics, he discussed the various theories which have been advanced as to its action, and rejected the idea that it was decomposed and yielded its oxygen to the blood. A number of experiments was detailed upon the human subject, from whom nearly all the salt given was afterward obtained from the urine. "As a salt exceptionally rich in oxygen, it has, without decomposition, the valuable property *per se* by its mere presence, apparently by oxygenating or aërating the blood, and so, by restoring or exalting this vital character of the circulating fluid, influencing to a corresponding degree the nutrition and functional activity by the various tissues and organs of the body. Beyond this it does not appear to have any specific action in any disease." The beneficial effect of this agent upon inflammations of the throat and other mucous membranes has been established by experience. The ordinary lozenges are too strong and are liable to exert a caustic action upon the mouth; he uses only two and a half grains in each trochee, with white sugar. This in combination with arsenic internally is almost a specific in clergyman's sore-throat. It is a valuable tonic and stimulant in cases of cardiac debility and impoverished blood, as in anæmia and chlorosis. It is remarkable what effect this has upon the development of the fœtus, when given during the whole course of pregnancy. Several very interesting cases were cited.

Dr. H. A. Hare, of Philadelphia, Pennsylvania, said that potassium chlorate does not yield oxygen to the blood, and cited a case of poisoning in which bacteria was found in the kidneys.

Professor J. Solis-Cohen, of Philadelphia, Pennsylvania, said that he was much pleased with the paper, and was in the habit of using chlorate of potash in diphtheria. He believed that all the chlorides were useful in this disease.

Professor Traill Green, the President, advocated the use of sodium chlorate as a less depressing agent, all potassium salts being depressing. He asked if this salt is used in England.

Dr. Murrell, London, England, said that since the publication in the *Lancet* of Dr. Sainsbury's paper, some years ago, the sodium salt was used very largely in preference to the potassium chlorate.

Dr. G. L. Magruder recommended potassium chlorate in catarrhal affections of the bowels in infants in combinations with chalk mixture.

Professor Frank Woodbury, of Philadelphia, Pennsylvania, said that a therapeutic agent need not be decomposed in the body in order to influence nutrition (*i. e.*, sodium chloride). He believed that the potassium chlorate has a limited range of therapeutic usefulness, within which it cannot be substituted by any other salt. In some cases it is very depressing, and its indiscriminate use should be condemned, as collapse may be caused in an ordinary catarrhal pharyngitis, and thus lead to the idea that the case is really diphtheria. It is important to watch the renal secretion, since the salt is not very soluble, and where there is a deficieney of water, crystals of the salt may be deposited in the tubules and give rise to irritation and congestion of the kidneys.

Dr. Charles D. F. Phillips, of London, England, read a paper entitled

THE ACTION OF CERTAIN DRUGS ON THE CIRCULATION AND SECRETION OF THE KIDNEY.

This contained a number of very interesting experiments, made with Roy's onkometer, with caffeine, sparteine, strophanthin, digitaline and ulexin.* He concluded that the flow of urine is not so much dependent on the blood pressure as on the rate of flow of the blood in the renal vessels. With regard to this point, it is necessary to remember that, although such drugs as strophanthin producing a great increase in the force of the cardiac beats, yet these are very much slowed, so that it is quite possible that although the heart's action is stronger, yet the total amount of blood sent through any given organ, such as the kidney, in a given time, may remain the same. Whereas such a drug as digitalis, producing as it does a rise of blood-pressure and a contraction of the kidney vessels, may cause an

*Ulexin is an alkaloid from the gorse, *ulexis europæus*

increased quantity of blood to pass through the renal vessels. On this view one could find the explanation of digitalin being a diuretic, and strophanthin not being one.

Inasmuch, however, as spartein has not so marked diuretic action, we must also assume that digitalin must have some peripheral action on the secretory apparatus of the kidney.

His results were tabulated briefly as follows, in three divisions:

(A) DRUGS THAT FIRST CONTRACT, AND AFTERWARD DILATE THE KIDNEY.

(1.) Caffein—in small doses—induces in the stage of contraction a fall of blood pressure—in that of expansion, a slight rise; during the former the flow of urine may be arrested; during the latter it is always increased, such increase depending on dilatation of renal vessels.

(The possible arrest of secretion during the first stage is special to caffein, and may be induced by large or repeated doses).

(2.) Ulexin—one-sixth grain, greatly raises blood pressure during the first stage (that of contraction); in the second, expansion is much greater in degree but shorter in duration than under caffein, and is accompanied by brief but marked increase in urinary flow; the effective dose is limited by its toxic action on respiratory centers. Practically, excess of caffein induces only the first stage—excess of ulexin only the second.

(B) SUBSTANCES THAT DILATE THE KIDNEY, BUT TO LESS EXTENT AND MORE SLOWLY THAN CAFFEIN AND ULEXIN

are dextrose, urea, sodium chloride, and acetate, and probably all constituents of the urine.

(C) DRUGS THAT CONTRACT THE KIDNEY WITHOUT SUBSEQUENT EXPANSION.

(1) Digitalin, with *increased* secretion of urine (probably resulting from general heightened blood pressure).

(2) Spartein, with *diminished* secretion (in health at least.)

(3) Strophanthin causes slight temporary contraction, with no marked increase of secretion.

(4) Apocynëin, similar temporary contraction, and no definite incease of secretion.

(5) Turpentine; (6) adonidin; and (7) barium chloride give similar results.

In conclusion, it seemed to him that the plethysmographic method of experimentation is a valuable one for determining the exact action of drugs on the circulation, and one that deserves more attention than it has hitherto attracted.

Dr. Murrell referred to the importance of this study of diuretics, since so little is known comparatively of their effects. Diaphoretics are well understood, but the action of diuretics remains to be worked out. He could not understand the points of superiority of the instrument used over Marey's tympanum.

Dr. Phillips said that Roy's onkometer was simpler, easier to work, and more accurate in its results.

Professor Woodbury inquired if any estimation had been made of the solid ingredients of the urine excreted during the experiments. He regarded water as merely incidental. The urine of snakes is solid. It is of primary importance, in determining the value of a diuretic agent, that its effect upon the excretion of the urinary salts (urea, urates, creatine, creatinine, and allied bodies) shall be ascertained.

Dr. Phillips said that these experiments were yet in their infancy and incomplete, but at a future time the chemical composition of the urine will be communicated.

Dr. Samuel S. Wallian, of New York, read a a paper on

THE NEGLECT OF NON-MEDICINAL THERAPEUTICS, in which he urged the abandonment of drugs, and resort to baths, massage, electricity, and hygiene.

---

### SECTION ON DISEASES OF CHILDREN.

### Second Day—Morning Session.

Dr. Moncorvo's paper was read on

HEREDITARY SYPHILIS AND RICKETS IN BRAZIL.

In an extensive practice in Rio de Janeiro, and the province of that name, he belived that hereditary syphilis furnishes sixty per cent. of the cases of infantile disease. It is the most important factor of infant mortality, either directly or by the severity which it imparts to the diseases of children, and rickets makes its appearance in forty-five per cent. of the children that come under his observation. This does not accord with the opinion of Dr. Charles West, who stated in the congress of 1881, that while syphilis was common in Brazil, rickets was unknown.

More than two-thirds of the rickety children in his practice show signs of syphilis and it is rare to find an hereditary syphilitic child whose bones are not deformed by rickets.

Although not prepared to demonstrate the etiology of rickets on these grounds, he believed that hereditary syphilis is an important factor.

A paper by Dr. William Stephenson, of Aberdeen, Scotland, on

THE RATE OF GROWTH IN CHILDREN.

But little had heretofore been known in regard to the rate of growth in children, and nothing whatever of its clinical bearings. Many important questions arise in this connection. For instance, if a boy in a given year adds to his weight double or treble the number of pounds which he does in another year, is he on account of this increase of cell-activity the more or the less able to bear a strain, such as school pressure or physical labor?

By combining the tables of Dr. Bowditch and those of the Anthropological Committee of the British Medical Association, Dr. Stephenson had tried to construct a standard of the rate of growth from the fifth to the eighteenth year, for all the English-speaking races.

In the charts exhibited, the graphic line representing the annual increase in weight presents a curve of similar type in girls as in boys, but differing in the times of maxima and minima. The most striking difference is seen in the fact that the maximum rate of growth occurs in girls from the eleventh to the thirteenth year, and in boys from the fourteenth to the sixteenth year.

From his study of the tables and charts the author is of the opinion that the critical and trying character of the period known as puberty, is due to the fact that the great activity of growth which occurs then makes a serious demand on the system, rather than to the fact that the reproductive organs are about to reach complete development. Reference was made to important results to be obtained from comparisons in rate of growth between children of the poor and rich, and of the professional, commercial, and artisan classes.

A paper was then read by Professor Victor C. Vaughan, of Ann Arbor, Michigan, on

THE USE OF COWS' MILK IN THE ARTIFICIAL FEEDING OF INFANTS.

Three years ago the writer had isolated the active principle from poisonous cheese. He had named it tyrotoxicon. Later he found the same principle in milk, ice-cream, and other articles of food. In experimenting with this poison it was found that its action on the lower animals produced the phenomena of cholera infantum. The symptoms and the post-mortem appearances were identical. From this it is easy to understand the prevalence of cholera infantum among the very poor, where fresh, wholesome milk is almost unknown.

Not a few medical teachers advise the prohibition of milk during the progress of cholera infantum, basing their opinions on clinical experience. The same view had been reached by the author of the paper through a long series of laboratory experiments, which show that normal milk inoculated with a small portion of poisoned milk and kept a few hours at the temperature of the body becomes itself poisonous.

Dr. Lewis P. Bush, of Wilmington, Delaware, advocated the use of the milk of a young and healthy cow, isolated from others, as in a herd there was a possibility of the contraction of some disease which might injure the milk.

Dr. R. B. White, of Ennis, presented the following rules which his experience had suggested:

1. Test with litmus, and add lime water, if required, before every meal.

2. Limit the amount given in twenty-four hours.

3. Allow only bottles large enough for a single meal, four to six in number; those not in use to be washed in boiling water and kept in an alkaline solution or suspended in the sunlight.

4. The best nursing bottle is a two-to-six-ounce flask, with a rubber nipple drawn over its mouth.

5. Bring the milk to the boiling-point, and then keep it on ice.

Dr. A. E. Goodwin, of Rockford, Illinois, was in the habit of insisting on the milk of one healthy cow well fed and stabled, on boiling the milk and keeping it in a sealed glass vessel at a temperature not higher than 60° F., and diluting it with from one-third to one-half of water with, perhaps, the addition of a little malt.

Dr. W. D. Booker, of Baltimore, Maryland, stated that there can be little doubt of the injurious effects of micro-organisms upon milk, and the best way to prevent this is not to trust to keeping the milk cool, but to first boil the milk in a flask supplied with a sterilized cotton stopper or a suitable sterilizer. It can thus be et aside and kept for aconsiderable time in a pure condition.

He believed it was important, in considering the injurious effects of decomposing milk upon children, not to overlook the danger of other injurious articles of food, which we know to be indigestible by children, and which are probably more often the first cause of the indigestion.

The President, Professor J. Lewis Smith, asked the writer of the paper in regard to the post-mortem appearances in the animals who had died from the effects of tyrotoxicon, and especially in regard to the mucous membrane, which, in patients dying from cholera infantum, is pallid after an illness of twelve or twenty-four hours, and injected if the disease had lasted three or four days.

Professor Vaughan replied that the mucous membrane of the stomach and intestines had been, as a rule, pale, and even almost white. In cases where the animal had survived a longer time, the membrane had been congested, but never in a very marked degree. The President added that, contrary to the opinion of some good observers, he entertained the conviction that the disease is inflammatory in its nature.

---

Afternoon Session.

Dr. William P. Northrup, of New York, read a paper on

THE PATHOLOGICAL ANATOMY OF LARYNGEAL DIPHTHERIA AS RELATED TO INTUBATION.

In autopsies of children who died after having worn the O'Dwyer tube from three to seven days, there was found merely an abrasion of the superficial epithelium. This was the invariable rule until the occurrence of an epidemic of measles, in which there were many deaths from complications of pneumonia, scarlet fever, diphtheria, and nephritis. In these children, debilitated by complications, the lower end of the tube thrown against the anterior wall of the trachea, produced instead of the slight abrasions noticed in the other cases, ulcerations which, in five cases out of twenty-six, exposed the cartilage.

Dr. Northrup summarized his paper as follows:

1. The cases here studied are 116 in number, 90 of which were sporadic, 26 epidemic. They all occurred in the New York Foundling Asylum, an institution which has the constant care of eighteen hundred children from the ages of a few weeks to five or seven years.

2. In the sporadic cases the laryngeal tube of Dr. O'Dwyer caused no ulceration worthy of consideration.

3. In the epidemic cases, numbering twenty-six, there were five serious ulcers.

4. The causes of death have been mostly extension of pseudo-membrane to the bronchi, and pneumonia.

5. I have been able to find no evidence that milk or other foreign material has found its way into the lungs. The pneumonia is broncho-pneumonia, and not schluck pneumonia.

A paper by Dr. E. Bouchut, of Paris, France.

ON TUBAGE OF THE LARYNX IN STRICTURE AND IN THE ASPHYXIA OF CROUP

was read. He reported that in the year 1858, he had tried the effect of tubage of the larynx in a number of children. He had three recoveries in ten cases. He relates one case in detail. The child was eighteen months of age. The symptoms were very serious. The face was livid, and the diminution of sensibility indicated approaching dissolution. A silver canula was introduced on the end of a sound, with a silk thread attached for extraction. It was coughed up and replaced, and on the sixth day was finally removed, and the child made a complete recovery.

Dr. Joseph O'Dwyer, of New York, N. Y., read a paper on

INTUBATION OF THE LARYNX.

He began experiments in 1880, at the New York Foundling Asylum. At that time tracheotomy was in disfavor at the asylum, because for some time its usefulness had not been demonstrated by a single recovery. The difficulty and danger of tracheotomy deter many physicians from the operation, and not a few even of the laity fail to understand how a child's suffering can be relieved by cutting its throat.

He first used a prostatic catheter introduced by the nostril. It was found impracticable, partly because the patient would remove it with the hands. The long tube suggested a short one, and its practicability was almost at once demonstrated by the construction of a tube one end of which extended into the trachea, while the other occupied the vestibule of the larynx, permitting the epiglottis to close over it in the act of swallowing. But what was to hold it down in place against the expulsive effort of a cough? First a bivalve form was given to its lower extremity, permitting of its expansion after introduction. But the tube was thus made a trap for fragments of pseudo-membrane. Longer tubes reaching nearly to the bifurcation of the trachea were kept in place better, but after a cough the long tube would be found thrown partially out of place, and had to be pushed back with the finger. A second shoulder was then added to the tube, below the expansion or head at its upper extremity. This shoulder kept the tube from being repelled, but the abruptness of its upper border made extraction very difficult.

The abrupt shoulder then gave way to a gradually tapering enlargement, which was found to accomplish all that could be desired. A tube constructed in this way, when projected upward by coughing, will slip back into position by the pressure of the vocal bands on the sloping sides, aided by its weight. This retaining swell is only made thick enough to hold the tubes loosely in the larynx, in order to permit of their easy expulsion in case of sudden occlusion by masses of pseudo-membrane too large to pass through. Owing partly to this and partly to the difference in the size of the larynx in different children of the same age, they will sometimes be expelled even when unoccupied by false membrane, and before the laryngeal stenosis is fully relieved. If it were not for this, such a tube could be indefinitely worn. A larger and shorter tube would not be expelled, but it would make serious pressure on the lining membrane of the larynx. Such a tube may be temporarily applied with advantage as a laryngeal dilator, not in ordinary severe croup, but in slow cases where little help is required.

Intubation is apparently, but not really, a simple operation. With more than the usual dexterity and coolness, and an easy case, it will be called by the physician who tries for the first time, a very simple thing. With less dexterity and a difficult case to manage, it will be called a difficult operation.

When established, and perfected, and in common use, intubation can never be considered a satisfactory remedy, in view of the complications and the very nature of membranous croup. The first results, if good, will create enthusiasm; if bad, distrust.

In comparing tracheotomy and intubation, the question is not which will save most life in a given number of cases submitted to treatment, but which operation can be performed or will be permitted in the greater number of cases.

A paper was then read on

INTUBATION OF THE LARYNX, ITS ADVANTAGES AND DISADVANTAGES, WITH STATISTICS OF THE OPERATION, by Professor F. E. Waxham, of Chicago, Illinois, while strongly advocating intubation, the writer would not overlook its disadvantages. The operation is a difficult one in its performance. The soft tissues may be wounded, or the trachea perforated. It is more difficult to extract than to insert the tube. A serious trouble may ensue from the difficulty of swallowing when the tube is in place. These are the dangers. None are so grave that they cannot be overcome.

On the other hand, it has many advantages over tracheotomy. It can be done almost instantly. There is no loss of blood, no pain, no shock, no open wound with the possibility of septicæmia or erysipelas. There is no drying of mucus in the tube, and no necessity for cleansing the tube. Less attention is required in the after-treatment. Finally, we can save as large a number of adults as, and a much larger number of children than, by tracheotomy.

After presenting a series of statistics, the writer closed with the statement that intubation has been performed one thousand times within two years, and that two hundred and sixty-nine lives have been saved from certain death.

Dr. Charles G. Jennings, of Detroit, Michigan, expressed high appreciation of the labors of Dr. O'Dwyer and Professor Waxham, and believed this method to be of great value. His own experience, however, had led him to prefer tracheotomy. His thirty-six tracheotomies had been followed by seventeen recoveries, while he had applied the tube in twelve cases without a recovery. Professor Waxham's statistics contain about one thousand cases, with twenty-six per centum of recoveries. He believed that certain operators in both operations have records far above the average, and that the successful tracheotomists have a larger proportion of recoveries than the physicians performing intubation who rank as the most successful. He believed that the personal equation is an important element in considering the comparative value of the two procedures.

Although intubation possesses the advantages alleged in the papers read before the section, it remains true that tracheotomy has advantages which should not be forgotten. After an incision the trachea is accessible for the removal of membrane and dried mucus, and direct medication by instillations and powders. Adequate nourishment after intubation is almost impossible, while there is no such difficulty after tracheotomy.

An obvious advantage of intubation is that it can be performed by many physicians who would not undertake a tracheotomy and in this way relief, impossible otherwise, will be afforded in a great many cases. His own experience, however, leads him to urge tracheotomy in all cases offering reasonable chance of success, and to reserve intubation for the cases in which operative interference will give nothing more than euthanasia, and for those patients whose parents refuse consent to tracheotomy.

Dr. T. J. Pitner, of Jacksonville, Illinois, questioned the correctness of Professor Waxham's closing statement that the 249 cases of recovery following intubation were so many lives saved by the operation. This assumes that, that all cases of stenosis from croup or diphtheria are, without operative procedures, necessarily fatal. He did not, however, question the utility and great importance of this operation, which appears to him to be preferable to tracheotomy.

Dr. Northrup stated, in answer to questions, that in thirty-three cases he had never met the slightest difficulty in inserting the tube, and had not failed in a single case to fully and promptly relieve the dyspnœa. He had met with no accident, and probably gentlemen present had in the aggregate used the tube several hundred times had never met with untoward accidents. He thought the only real drawback to the success of intubation lay in its interference with feeding. It is sometimes difficult to keep up full nutrition when swallowing is so impeded.

In one of his cases the physician in charge had retired hopeless, without doing tracheotomy, and being ignorant of intubation. The child was exhausted and comatose. The tube was inserted.

The child returned to consciousness. At the end of forty hours he coughed out the tube. It was not reapplied, and the child made a perfect recovery.

Dr. O'Dwyer, in answer to questions, said that, in his opinion, statistics will be of very little value in settling the question between tracheotomy and intubation until a very large number have been obtained. Aside from the question of saving life, intubation will be resorted to in the most hopeless cases, those in which tracheotomy would not be thought of, for the sake of securing euthanasia.

---

### SECTION ON MILITARY AND NAVAL SURGERY AND MEDICINE.

### Second Day—Morning Session.

The first paper was read by Dr. Robert Reyburn, of Washington, D. C., entitled

#### ARE WOUNDS FROM EXPLOSIVE BALLS OF SUCH A CHARACTER AS TO JUSTIFY INTERNATIONAL LAWS AGAINST THEIR USE?

The proper discussion of the subject, the speaker said, was largely influenced by the changed conditions of modern, as compared with ancient, warfare. The chief endeavor in the battles of ancient times was to kill as many of the enemy as possible in a given time. The same end is attained in modern warfare; but it has been found that an enemy wounded sufficiently to be *hors de combat* is practically a much greater source of weakness to his comrade than if he were slain in battle. Every wounded man requires the services of at least two able-bodied men for his care and sustenance until he again becomes fit for military duty. The speaker graphically described the characteristic wounds produced by explosive projectiles, which are generally followed by extensive sloughing, which reduces the chances of recovery. Such projectiles were in use during the War of the Rebellion, but were soon abandoned; some thirty-three thousand of Gardner's explosive bullets were distributed during the early part of the war, but were soon withdrawn. During the year 1868, by an agreement made between the principal nations of Europe, at an international military conference held at St. Petersburg in October of that year, all the great powers resolved to abstain from the use of explosive projectiles in war under the weight of four hundred grammes. The author concluded by saying that common humanity demanded that the use of such projectiles be prohibited by international law.

A paper on the same subject, by Dr. Charles W. Voorhees, of New Brunswick, New Jersey, was read by the author, whose opinions were, in the main, those of the preceding speaker. He thought that the wounds produced by explosive projectiles, aside from the great destruction of tissues attending them, were doubtless aggravated by the chemicals to which the explosive properties were due. He did not wish to be understood as advocating child's play in war, but so long as war was a necessary evil, it should be as humane in its methods as possible.

The discussion of the papers was opened by Dr. Marston, of the British War Office, who said that he was not aware that explosive bullets were in use by any civilized nation at the present day; they were in use in India and Africa for the killing of large game, but he had not known of their use since the Crimean war.

The President, Professor H. H. Smith, stated that it was not generally known that the late President Lincoln, early in the year 1863, had issued a notice, or perhaps only a suggestion, that the use of explosive projectiles be discontinued. He was glad to see this question brought before the consideration of the congress, and as the United States had not been represented at the International Military Congress, at St. Petersburg, he advised that the powers be notified through their representatives of the entire acquiescence of the present congress with the views and agreements then enunciated.

Dr. Jeffrey A. Marston, of England, then read a paper on

#### AGE AND ACCLIMATIZATION OF SOLDIERS IN REFERENCE TO SERVICE.

With regard to age, the author said that a man was best fitted for service, at least in India, between the age of twenty-seven and thirty years. He alluded to the well-known fact that the younger men, although capable of enduring great hardships and privations for a short period, are inferior to adults in their powers of endurance in long-continued efforts.

The influence of climate upon the soldier was discussed at length, the speaker more particularly describing the effects of Indian climate upon the newly arrived soldier. He presented tables showing the mortality among soldiers in Indian service, stating that the prevailing disease among the soldiers is enteric or typhoid fever. This disease may be said to have no geography, extending, as it does, from the Rocky Mountains to the Himalayas. It prevails among Europeans, and is also more frequently fatal during the hot and cold seasons; according to the experience of Indian surgeons, the greatest period of susceptibility lies between the age of eighteen and thirty years. The liability of death during his first year of Indian service by this disease is greater than by all other diseases.

The British troops suffered greatly in Egypt during the campaign of 1882; in the Soudan campaign of 1884, the troops suffered less from this cause, being daily supplied with condensed water; but in 1885, in spite of this same precaution and of great attention to sanitary details, for some reason still unaccounted for, the sickness from this disease was excessive.

During the first year of the young soldier's sojourn in India, he is likely to be attacked by a fatal form of pneumonia. But of the prevailing affections next to enteric fever, hepatitis heads the list, this disease being very commonly met with. Cardiac disorders come next in the order of prevalence.

In the discussion which followed, Dr. Morse K. Taylor, United States Army, stated that his experience agreed with that of Dr. Marston in regard to the prevalence of heart affections among soldiers; this fact, he thought, was not sufficiently recognized by our own surgeons.

Surgeon-General G. T. Langridge, of the British Army, fully endorsed the view of Dr. Marston with regard to the greater power of endurance of the adult men over the younger; these latter, he said, were constantly breaking down when marching, particularly when exposed to the heat. During the Afghan campaign the troops suffered more from enteric fever than from any other disease.

In the absence of the authors, the next three papers were read by title. The first of these was entitled:

"Is it desirable that each soldier in time of war, should personally carry a first field-dressing for a wound? If so, it is advisable that a preliminary wound-dressing should form part of the equipment of every soldier on taking the field? Of what shall it consist, and in what part of the soldier's equipment should it be carried?" by Sir Thomas Longmore, of Netly, England, Professor of Military Surgery at the Army Medical School. The paper was accompanied by a dressing devised by the author for this purpose.

The second paper by Professor Von Esmarch, of Kiel, Germany, entitled "On the First Provisional Dressing on the Battle-Field," and was also accompanied by a simple antiseptic dressing devised for this use.

The third, entitled, "On the Antiseptic Treatment of Wounds in War," was by Dr. M. W. C. Gori, of Amsterdam, Holland.

Dr. John Anderson, of London, England, read a paper

### ON HEAT-STROKE IN INDIA.

After adverting to the labors of Professor H. C. Wood, of Philadelphia, Pennsylvania, upon this subject, he proceeded to a definition of the term heat-stroke, and divided his subject, according to the degree of severity of the disease, into ardent fever, heat, apoplexy, and sunstroke. The first is characterized by a high fever of short duration, and by great exhaustion; the second, by all the signs of apoplexy, and causing death by asphyxia; the third, by great cardiac debility and nervous manifestations.

These are all pathologically allied, of course. The speaker briefly alluded to the influence of alcohol in the causation of the disease, which is generally supposed to be due to the direct action of heat upon the meninges of the brain, but the condition of the glandular organs, of that regulator of the heat, the skin, were factors which should not be overlooked.

With regard to the prophylaxis of the disease, protective measures, of course, were all-important, moderate eating, the wearing of thin clothing, and the importance of free ventilation of houses and camps. The author inveighed against the use of alcohol, and stated that the best drink which could be devised for soldiers, while on the march, was cold tea flavored with lime-juice. Regarding the medicinal treatment of cases of heat-stroke, the first indication is to relieve the excessive heat, and for this purpose he had had great experience with the use of a neutral salt of quinine, the hydrobromate, administered hypodermatically in doses of two, three or four grains, repeated according to indications. The speaker emphasized the necessity of using a thoroughly clear solution, filtered if necessary; when this precaution was taken, any untoward symptoms from the use of the needle, other than slight local irritation in some rare instances, were exceedingly uncommon in his experience. As for the occurrence of tetanus as a result of these punctures, he himself had never seen a case of it. The speaker was at a loss to explain the manner in which the quinine reduced the temperature so rapidly in such cases, but thought it probably due to its action upon the cardiac ganglia. After the administration of quinine, the portal congestion usually existing should be relieved by mercurials, and the nervous condition combated by means of the bromides of potassium and ammonium.

---

### AFTERNOON SESSION.

Dr. Marston, of England, opened the discussion of the paper by indorsing the view of his compatriot with regard to the efficacy of quinine in the disease, and reiterated the author's cautions in using a perfectly clear solution.

There had been a number of cases of tetanus published in the report of the army in India as due to the use of the needle; such a complication could be avoided by the use of a pure solution and clean instruments. In conclusion, he alluded to the fact that persons who had had sunstroke were thereafter liable to pyrexiæ, without apparent cause. Strong men became nervous and timid, and suffer from insomnia and dreams; they are occasionally subject to neuralgias and excruciating headache after exposure to the sun; he had even noticed, in some cases, paroxysmal attacks of mania. To such suffers he always advised removal to cooler climates.

Professor Henry Ernest Goodman, of Philadelphia, Pennsylvania, had noticed, during the late war, many cases of so-called heat-stroke, in which he almost invariably found some cardiac trouble, sometimes accompanied by a *bruit* at the apex or base; he thought that in these cases the cardiac affection accounted for the heat-stroke, so-called. In his experience during the war, cases of true heat-stroke were exceedingly uncommon.

Dr. Moses K. Taylor, U. S. Army, fully concurred with the preceding speaker in his views, which were in accord with his own observations during the late war.

Dr. Sherwood, of the U. S. Pension Office, spoke of the results of sunstroke as shown by the records of the Pension Office. Many claims have been, and are still received, allegating nervous diseases, such as epilepsy, paralysis agitans, and even locomotor ataxia as the result of sunstroke. He was unable to trace the connection between these diseases. The literature of this subject is exceedingly meagre, and in order to determine the exact relation between sunstroke and nervous affections, if any there be, he enjoined physicians, both civil and military, to keep a record of such cases, and to follow them in their complications as far as possible.

Dr. James Collins, of Philadelphia, Pennsylvania, expressed the opinion that occipital headache was frequently attributable to sunstroke. For the treatment of the disease he had used quinine, hypodermically, in a solution of the bi-muriate rendered neutral by the addition of a small quantity of urea.

Dr. George T. Langridge, of England, had found the solution of quinine recommended by Dr. Anderson very useful in similar cases—in the Afghan and Boer campaigns, as well as in India. In the latter country, when the disease resisted all ordinary forms of treatment, quinine was almost a specific.

Dr. Eli A. Wood, of Pittsburg, Pennsylvania, had found that after removing the clothing the application of ice externally, and even in the rectum, had proved of greater service at his hand than any medicinal treatment.

Dr. Max J. Stein, of Philadelphia, Pennsylvania, belived that manipulation and rubbing the body with ice produced good results. He believed that a temperature of 111° F., mentioned by Dr. Anderson as necessarily fatal, did not, according to Dr. Austin Flint, always so result.

Dr. W. H. Lloyd, of England, agreed with Dr. Anderson regarding the influence of alcohol in the causation of this disease. His experience in the navy had been that most of the causes of heat stroke—those occurring on shipboard in the Red Sea at least—were among the cooks, stokers, bakers, etc., who were men of intemperate habits.

Dr. Anderson, of England, author of the paper, thought that no surgeon would neglect the usual means of treatment, but that he had desired to lay special stress upon the hypodermatic use of quinine as the readiest and best means at our command of reducing the high temperature.

---

### SECTION ON LARYNGOLOGY.

### Second Day—Morning Session.

J. P. Klingensmith, M. D., of Blairsville, Pennsylvania, read a paper on

### HAY-ASTHMA.

In few diseases does he find so diverse opinions or so many methods of treatment as in hay-fever. Dr. W. H. Daly's paper in 1881, first demonstrated the underlying naso-pharyngeal causes. The exciting cause does not work where nasal mucous membrane is healthy. One must remove the pathological cause before a cure can be be effected. Dr. Hock, of Freiburg, Germany, had placed much importance upon the pathological condition of the nasal mucous membrane as the fundamental cause. From personal experience, Dr. Klingensmith would place local irritation, hypertrophies, deflected septum, etc., as the starting point, and dust, pollen, light, heat, etc., as the exciting cause. There must be an abnormal condition before local irritants have power. Always make careful examination of anterior and posterior nares. Radical treatment is called for, especially where there is more or less occlusion.

The cautery and Allen's or Jarvis' snare will bring about the best results under these circumstances. Sensitive areas he treats by the continued use of acetic acid or cautery. He uses it at a cherry-red heat; for if too hot one will have hæmhorrhage follow, and if too cool it will produce greater pain. Once in a week gives time to allay irritation by use of Dobell's solution, etc., before another sitting. He begins treatment at least two weeks before an expected attack, and even treats or operates when the disease is at its height. Out of thirteen cases, he cured nine and greatly benefited four.

Dr. Lennox Browne, of London, England, said that he had heard no new ideas on the subject since the president's paper of six years ago. He does not think that sexual irritation can cause or in any way interfere with hay-asthma, but may do so in nasal troubles. He believes in local treatment and electricity. Dull, red heat he likes best.

Professor E. F. Ingals, of Chicago, Illinois, says that it makes no difference whether the electrode is at a white or red heat as far as hæmorrhage is concerned, provided that one does not break the circuit before the wire is removed from the tissues.

Professor W. E. Casselberry, of Chicago, Illinois, agrees that it makes no difference whether red or white heat is used. The cause of disease is located in diseased mucous membrane or sensitive areas, and the case must be treated several months, till all are removed or destroyed.

Dr. J. MacKenzie, of Baltimore, Maryland, stated a case of a woman who had been under his care, who always had attack of hay-fever whenever exposed to the pollen of rose, and even the sight of it would cause a violent attack. He caused a perfect artificial rose to be made, and one morning when the patient came to the office, entirely free from hay-fever, he placed the rose near by, and in less than a minute her nostrils were occluded, eyes reddened and suffused, and she was in the midst of a bad attack. Air was pure, and there was no dust on the flower.

After having been informed of the trick, fresh pollen put on the flower had no effect, and since then she can uses roses without trouble. Sensitive areas are most common on the septum; sympathethic and vaso-motor nerves are those involved. Rose and hay-fever and cough, autumnal fever, and asthma are all the same nasal neurosis.

Professor J. Solis-Cohen, of Philadelphia, Pennsylvania: There are too many specialists and not enough practitioners. Lack of equal nerve-tension and action of vaso-motor system upon blood-supply to vessels irritates and predisposes to hay-fever.

Treat the constitution, and especially the nervous system, through interval till next attack.

Dr. Ridge, of Camden, New Jersey, said he was a victim, and in his case it was hereditary for several generations. Any dust or pollen will cause an attack, and cocaine gives best results in relieving, especially when used in saturated solution of boracic acid (gr. xv. to ℥ j).

Quite a large portion of the section acknowledged themselves victims of hay-fever. Inhaling a mixture of the following: acid carbol., chloroform, camphor, gave one the greatest relief in one case. Imagination plays an important part as well.

Professor Stockton, of Chicago, Illinois, gave as causes, (1) highly nervous temperament; (2) diseased air-passages; (3) peculiar external irritants (varies with person). Cold to his own head and neck would cause sneezing for half an hour and other symptoms resembling hay-fever. It could be cured for a time, but paroxysms would return.

Dr. Thomas closed the discussion: It is not simply hyperæsthesia, though the nervous factor greatly increases the severity of the attack, and treatment must be constitutional, and especially directed to the nervous system.

---

### Afternoon Session.

Dr. N. Rankin, of Alleghany, Pennsylvania, read a paper entitled

#### SOME REMARKS ON THE HISTORY OF RHINOLOGY.

Since the nasal cavities play so important a part in respiration, it is surprising that rhinology should have been so neglected in the past. As organs of smell they are to protect the lungs from deleterious gases and assist organs of taste, stimulating appetite and even digestion. The reader compared the perfect instruments of to-day with those of thirty years ago, when a tongue-depressor and clumsy polypus forceps worthy of the village smith had to suffice for many. The art is old, even the Egyptians had some idea of treatment. Professor Czermak, of Pesth, conceived the rhinoscope, after which much was learned of what was before neglected and obscure. Now local applications can be made to the posterior nares with more ease than formerly to the pharynx. The writer paid tribute to the memory of the late Dr. Louis Elsberg as the father of rhinology and laryngology in America.

A special discussion was opened by Professor E. F. Ingals, of Chicago, Illinois, on the subject of

#### EPISTAXIS.

He opened the discussion, he said, for the purpose of bringing out new ideas on the subject, if possible, from others.

Ordinary cases are of no moment, and alarming only in young children and where the system is run down. Severe cases may demand constitutional treatment of ergot, gallic acid, etc., in additions to local applications. Most of cases readily checked by cocaine or tannin. Persulphate of iron is irritating; it causes hard, coagulated masses, beneath which the bleeding contin ues and which interfere with the use of more effective remedies. An effort should always be made to locate the bleeding-point, and which should be cauterized with galvano-cautery, nitrate of silver, iodine, or pure carbolic acid.

In the discussion that followed various methods were suggested for mechanically controlling the hæmorrhage.

### SECTION ON PSYCHOLOGICAL MEDICINE.

### Second Day—Morning Session.

Dr. E. C. Spitzka, of New York, N.Y., read a paper on

#### MILIARY ANEURISM.

He reported a case which came under his observation in 1886, that of a girl twenty-four years of age, who suffered from insomnia and anorexia and great motor weakness, being scarcely able to cross her room unaided. She had suffered from what appeared to be hallucinations at night for seven years, and during that period it was noticed that from time to time she dropped articles which she was carrying. She would often totter and sway from side to side. From March, 1886, she became poorly nourished. Her menses ceased in July, and remained absent until her death. Her brother had also died of a cerebro-spinal disorder, and her father had died at an advanced age with many of the same symptoms. On post-mortem examination there was found no evidence of softening processes having occurred at any time, although there was found slight grayish discoloration in parts of the substance of the cord.

Dr. Spitzka next reported a case of

#### DEFORMITY OF THE BRAIN, with specimen of the cerebellum, in a child aged five. He gave some very interesting details of the anatomical peculiarities, and stated that there was scarcely an organ in the abdominal or thoracic cavities which was normal.

Dr. Savage, of London, England, discussed Dr. Spitzka's first case and cited a somewhat similar one, though without the direct inheritance spoken of in Dr. Spitzka's case. It was that of a lad who developed all the symptoms of disseminated sclerosis—in fact was for many years the stock case in the hospital just as soon as the lecturing season commenced. This case was sent to an asylum; was excited and maniacal. After six or eight months he lost control over his sphincters, and presented other symptoms that were typical of the disease referred to; and this was diagnosed after his movements and peculiarities had been carefully noted. He finally died, and upon post-mortem examination no insular sclerosis was anywhere visible, neither were there allied changes; there was absolutely nothing present that would indicate insular sclerosis. He said it was now admitted that in cases of nervous disease it is rather the order of the disease than the quality that is worthy of consideration; that a widely spread disease affecting the motor or the general tract, especially the motor tracts, will give incoordination, and would give rise to symptoms which in former years would have been attributed to the cerebellum.

Dr. Daniel Clark, of Toronto, Canada, read a paper on

#### REMISSIONS AND INTERMISSIONS IN INSANITY.

He said there can be no vital and psychic energy without its presence and co-operation. It is an indispensable condition. When the rigor mortis of death sets in it takes its flight, hence the evidence of its intimacy with, and necessity to, vitality. It has, in normal physiological operations, seasons of remissions and intermissions, and it determines their intensity and duration in organic life. In chronic pathological conditions the same law exists, but it necessarily, by virtue of low vitality attended with excessive energy, makes the intervals more extended and the symptoms more pronounced in the ever-recurring periodicity and alternations. Dr. Clark treated of a trinity of forces—chemical, psychic, and vital forces—and believed the lower forms embraced the greater.

Dr. Blandford, of London, England, asked Dr. Clark to explain his theory and principle more fully. He differed with Dr. Clark on many points of his paper. He had seen many patients have intermissions of exactly the same form of mental disorder, and who were not subject, as Dr. Clark had claimed, to different forms of mental disease at each remission; and instanced one case familiar to him, of a patient who had been sent to one of the English asylums on thirty-five different occasions, suffering with precisely the same form of mental disorder, mania, upon each commitment.

Dr. Clark stated that his experience had been different from that of Dr. Blandford. He had never seen a patient suffering from *folie circulaire* who enjoyed during intermissions normal mental health. Patients had said as much to him; they had been able to transact business during intermissions, but had lost their former grip on affairs.

Dr. Savage differed quite widely from the sentiments expressed by Dr. Clark, particularly as related to animal magnetism, which, he said, was not at present well defined. The question of the correlation of forces was an important one, and should receive much attention. Dr. Savage cited several cases in which the remissions in *folie circulaire* were complete. He also cited the case of a patient whose disease was diagnosed as general paresis, and who appeared to be steadily going down hill, but who, after the development of a large carbuncle upon his shoulders, quite recovered, and lived outside transacting business for seven or eight years, when he died of some nervous disease.

Dr. Ferguson next discussed Dr. Clark's paper. He did not believe it possible to explain the rhythmical phenomena of intermissions and remissions of mental disease by Dr. Clark's theory.

Dr. C. H. Hughes, of St. Louis, Missouri, thought the obstacles which enter into the consideration of Dr. Clark's paper consisted in the barrier which physiology has placed in the way of allying physical organism to chemical organism, and which consists in the basil-motor mechanism and the part which it plays in neuro-pathology and neuro-physiology.

Dr. Clark was glad that his paper had succeeded in evoking discussions upon the subject of the phenomena of remissions and intermissions. He had not assumed that the key presented by him would open the lock. On the other hand, he did not see that the gentlemen who followed him offered any solution of the mystery at all. He believed that when a man suffered from an attack of insanity a post-mortem examination would, in every case, reveal changes in the brain, no matter whether it was claimed the man had recovered or not. Recovery is only a relative term.

The next paper of the session was by Dr. Horace Wardner, of Anna, Illinois, "Occupation for the Insane." He cited some cases from his asylum, upon whom the effect of judicious occupation was marked.

Dr. Bower, of Bedford, England, followed with a paper upon

OCCUPATION FOR THE INSANE IN ASYLUMS FOR THE PRIVATE CLASS IN ENGLAND.

There had been a change in these asylums since the time Dr. Bucknill had written that they were "castles of indolence," and in his own asylum Dr. Bower had succeeded so well in his plan of insisting upon his patients occupying themselves that he had lost only two patients who thought honest work derogatory.

The patients have attended to the cows, rabbits, and pigs, and in wet weather have done sawing, some painting, carpentry, and chaff-cutting, pumping and other work not of a menial character. The most suitable work has been copying legal writing. The women sew, knit, do fancy-work, china painting, etc.; some held the house-keeper to prepare fruit-preserves and pudding. Seventy-five to eighty-five per cent. of Dr. Gower's patients were occupied from two to three hours daily.

Dr. Andrews, the President, said there had been an enormous increase in the amount of occupation by patients in American asylums within ten years. The importance of the subject was now everywhere admitted. Seventy-five per cent. ot the patients in the Buffalo asylum are employed, and he believed a like percentage in other asylums. We use moral measures only in inducing our patients to work. We tell them it is for their good, that they are the happier for it, and we rarely have any trouble in the matter.

---

AFTERNOON SESSION.

The first paper was read by Dr. T. W. Fisher, of Boston, Massachusetts, on

MONOMANIA AND ITS MODERN EQUIVALENTS.

He gave the nomenclature adopted by various writers and alienists in this country and abroad, and in the course of his paper cited some interesting cases ot paranoia which had come under his observation. In Dr. Fisher's opinion there is:

First. A common form of primary insanity, with systematical delusions.

Second. There are many cases secondary to inebriety.

Third. Cases are reported secondary to mania and melancholia.

Fourth. They occur when no heredity can be proven.

Fifth. Hallucinations of hearing occur in three-fourths of all cases.

Sixth. Episodical excitement is very common.

Seventh. Monomania is the best English term for them.

Eighth. Such cases should be separately classified and reported.

Dr. Walter Channing, of Boston, Massachusetts, next read a paper by Dr. Bannister, of Kankakee, Illinois,

ON THE CLASSIFICATION OF INSANITY.

In Dr. Bannister's opinion all the classifications of insanity are necessarily more or less defective. A perfectly scientific or satisfactory one is an impossibility in the present state of our knowledge. The best one, in his judgment, was the clinical or, perhaps more correctly, the symptomatological one. Dr. Bannister then outlined in detail such a classification as in his judgment would best serve the purpose.

Dr. Channing then read his own paper on

THE INTERNATIONAL CLASSIFICATION OF MENTAL DISEASES, which he illustrated with a chart. He said the plan he had brought forward was that sanctioned by the Association of Asylum Superintendents at Saratoga, in May, 1886, and that in the opinion of foreign as well as American alienists, there should be a classification upon which all could unite. He not claim perfection for the classification here outlined, but thought it one which would answer the purpose very satisfactorily.

Dr. Yellowlees, England, thought that the classification would not be sufficiently clear. He deplored the fact that, owing to the incomplete knowledge of the subject of mental disease, alienists were still compelled to classify forms of insanity by their outward manifestations. Mania, melancholia, and dementia are simply labels for symptoms. Chronic mania often mixes itselfup with dementia. We know what we mean when speaking of mania, melancholia, and dementia, but beyond this the classification is valueless. Primary delusional insanity is not sufficiently truthful, as it often comes after melancholia. One cannot tell oftentimes between primary dementia and the acute stupor of melancholia.

Dr. Duquet did not understand why puerperal mania should be a distinct form in the classification any more than gouty or rheumatic insanity.

Dr. C. H. Hughes, of St. Louis, Missouri, said that alienists should go forward in their efforts to secure a clear and descriptive classification. Mania, melancholia, and dementia sufficed for courts of law, for jurymen, but science should not be kept down to such a classification from such considerations. The work of inaugurating reforms in classification was a thankless one. Every alienist has peculiar ideas of his own upon this subject, and does not readily incline to those of others.

Dr. Hughes then read an elaborate paper, entitled

THE TRUE NATURE AND DEFINITION OF INSANITY.

which was discussed by Dr. Yellowlees and Dr. Channing.

---

### SECTION ON SURGERY.

### Second Day—Morning Session.

Dr. John Homans, of Boston, Massachusetts, read a paper entitled

THREE HUNDRED AND EIGHTY-FOUR LAPAROTOMIES FOR VARIOUS DISEASES, a *résumé* of the writer's experience. In these cases he was in the habit of using drainage-tubes, the latter being cleansed once in about four hours, but he did not consider they drained the abdominal cavity, although Keith, of Edinburgh, has in his possession about two gallons of fluid secured in this manner from one patient. In his own practice, he had had one case of tetanus after laparotomy, the patient dying at the end of six days; one case of stone in the bladder; from hair falling in forming a nucleus. The greatest number of consecutive recoveries he had had was thirty-eight. He considered that suppurative cysts of the ovary very rare, only one having come under his notice, and he believed that they did not exist unless exposed to the air or a mucous surface. The removal of sessile tumors was quite a knack, and only acquired after long experience. The preference of procedure of the writer was the extra-peritoneal, using the wire ecraseur, and if the bladder were injured during the operation a soft catheter was retained in the urethra. In five cases of bleeding fibroids, one he had cured, the second relieved in part, and the third received no benefit. Another case in which the uterus was removed, the patient died. In one case of abscess of the ovary, the patient recovered; the writer called attention to that condition in which fæces exude from the umbilicus through a tube called the "omphalo-mesenteric remains," one case coming under his care. The patient afterward died from marasmus.

One case of tubercular peritonitis he had cured. He had had five cases of artificial anus, with three cures; one case of cystic tumor in which, during the operation and opening of the sac, the woman gave a violent cough, and the cyst was forced clean out by the effort. In one case of laparotomy, performed January 27, 1886, the patient was confined in December of the same year. A case of fibroid tumor of the uterus was gradually expelled through the wound in the abdominal wall. The speaker saw no reason why contractions should not occur in this manner as with any other foreign body in the uterus; the removal of uterine appendages in his own practice for nervous diseases had not given good results, only one case being cured; a woman who was very violent during her menstrual period, and who had commenced sexual intercourse at seven years of age, (?) was relieved of her mania, but her mind was weak. He had had two cases of myxo-lipoma, one in a man and the other in a woman; the man died eight hours after the operation. The speaker had performed laparotomy upon him once before, but refused to re move the tumor; the second time the man begged to be relieved of it, and the attempt wa made, fifty-two pounds being removed, and other portions were left. He had performed laparotomy once for perityphlitic abscess, and the patient recovered.

Dr. Addinell Hewson, of Philadelphia, Pennsylvania, presented a paper, which was read by the secretary, making the claim that the main point in laparotomy was the closing of the abdominal wound without sutures. This was done by means of binders' paste and a certain prepared gauze, called

DONNA MARIA'S GAUZE, which was first made some forty years ago; this gauze, however, costs $5 per yard. The writer was not partial to wet dressings, and the present paper advocated dry dressings.

Dr. I. M. Matthews, of Louisville, Kentucky, read a paper entitled

WHEN IS COLOTOMY JUSTIFIABLE?

The writer had had twenty years' experience in rectal surgery, and he did not consider colotomy justifiable in cancerous disease of the rectum when located three inches from the anus; again, not in stricture beyond the reach of the finger, nor in aneurism, nor in cases of specific origin. In most of these operations life was not prolonged; he had seen as many live as long without it as with it. The speaker considered the pain in cancer to be inherent. In the division of stricture, he did not consider there was any danger, as the hæmorrhage could be controlled. In small ulcers of the gut, he did not believe in operating, antiseptic and internal treatment being preferable. In congenital occlusion of the rectum he did not think it should be recommended, but that the perineal operation should be done. When the sigmoid flexure is blocked, the speaker thought it justifiable. The question then arises, What is to be done in the majority of cases for stricture? Linear rectotomy, not colotomy.

The paper was dicussed by Professor Dawson, of Cincinnati, Ohio, who agreed generally with Dr. Matthews. He had really never performed colotomy which gave him satisfaction. In syphilitic cases of stricture he had found they could be treated constitutionally.

Dr. Samuel Benton, of London, England, remarked that if he could get beyond the cancer he performed the operation, but if the growth was high up he performed colotomy. It had not been his experience that colotomy should not be performed in cancer of the rectum; his last case lived sixteen months after the operation; he considered pain was relieved by colotomy. Cases of benign stricture he treated by electrolysis.

### SECTION ON GYNÆCOLOGY.

### Second Day—Morning Session.

Thomas More-Madden, F.R.C.S., Ed., Dublin, Ireland, read on

THE CAUSES AND TREATMENT OF BARRENNESS.

Few gynæcological questions come so constantly before us, and none probably are of greater practical importance, than those connected with sterility, involving, as they do, not merely the physical health of the patient, but also in many instances affecting the happiness and welfare of married life. For, at least in the country where he practices, child bearing is still generally, and I believe rightly, held to be one of the chief functions of a woman's conjugal life; while to be sterile is commonly regarded as the protean source of marital troubles.

In his paper is given, in tabular form, a statement of the causes of sterility in five hundred and twenty-eight of the cases of infecundity which, occurring in married women within the child-bearing period, which had come under his observation in the gynæcological department of a hospital. The cases may be roughly divided into two classes.

(1) Those in which barrenness was occasioned by sexual impotency or some physical impediment from the vulvar orifice to the ovaria. (2), Cases of true sterility, or conceptive incapacity from deficiency congenital or acquired, structural disease, arrested developments, supra-involution, etc., of the uterus, or from analogous morbid conditions of its appendages. (3) Cases of barrenness from constitutional causes. (4) Cases in which the causes of infecundity were apparently moral rather than physical, such as sexual incongruity, etc.

According to this table the most frequent cause of sterility is stenosis of the cervical canal. He believes that the operative treatment of such cases simple as it is deemed by some, requires more consideration than it generally receives, and frequently proves worse than useless from the disregard of certain details and precautions which he considers essential. He recommends the use of a method of procedure and the adoption of instruments which he has found advantageous in the curative treatment of stenosis in 380 cases of obstructive dysmenorrhœa and sterility traceable to this cause.

The essential features of the method of treatment are the separation by cutting and simultaneous forcible expansion of the affected parts, followed by dilatation during the period of cicatrization, so as to prevent their subsequent contraction, and thus secure the permanent patency of the erst occluded passage. To obtain this result he uses three instruments, viz., a special form of uterine director which can, generally, be introduced into any cervical canal however narrow, and along which a serrated, triangular-guarded knife is made to travel up through the os internum; and thirdly, a uterine dilator of great power, by which any required degree of cervical expansion may be effectually secured and accurately gauged.

The influence of uterine flexions in the prevention of pregnancy and the treatment adopted in cases of sterility dependent thereon are next described. So also is the management of aphoria when it results, as is frequently the case, from chronic endometritis. The methods found most serviceable in infecundity due to vaginal, uterine, and ovarian causes are briefly reviewed. More fully dwelt on is the subject of conceptive incapacity from morbid conditions of the fallopian tubes, as he regards stenosis, as well as occlusion of those ducts by vaginitis and its results, such as hydro- and pyo-salpinx, far more common causes of sterility than usually thought. He also holds that such tubal diseases may often be removed without the resort to the serious operative procedures as the removal of the uterine appendages, by some surgeons considered invariably necessary and by them freely employed in such cases. He therefore referred at some length to those less heroic measures, such as aspiration and catheterization of the fallopian tubes, the feasibility and successful results of, which he said he had clinically demonstrated.

Finally, the question of sterility arising, as it not infrequently does, from constitutional disorders, and instances apparently irrespective of any physical cause, and the method of dealing with such cases, is discussed.

Dr. S. C. Gordon, of Portland, Maine, said he did not believe that cases existed where there was not enough canal for the semen to pass up. He believed with Dr. Madden that vaginismus had very much to do therewith. We must remember that we cannot raise large crops on barren soil. No one has done more for the relief of these conditions than Graily Hewitt, who is now with us. The uterus is in an abnormal position, and you must return it to its normal position, as is his custom to do so effectually with his pessary. Above and beyond this comes the fallopian tubes and ovaries. The points concerned are, first, vaginismus ; second, uterine or pelvic congestion. He is convinced that it is the fallopian tubes and ovaries, more than the stenosis of the cervical canal that are at fault.

Professor Graily Hewitt, of London, England, described this method of treatment carefully, and expressed his conviction that the good done was through the straightening of the uterus, not the dilatation.

Dr. Lapthorn Smith, of Montreal, Canada, thought that in many cases the difficulty did not lie in the vagina, cervix, uterus, fallopian tubes, or ovaries, but in the testicle.

Dr. Daniel T. Nelson, of Chicago, Illinois, thought with Dr. Smith that in many cases the male was at fault. If the mucous membrane of the female is pale, anæmic, contracted, cicatricial, containing little blood, the sperm is not nourished, or if so, only for a few days. Sea-bathing often does good.

Professor A. Reeves Jackson, of Chicago, Illinois, read a paper on

THE MODERN TREATMENT OF UTERINE CANCER.

Correct views of pathology and accurate diagnosis form the only rational grounds for proper treatment of disease. The modern treatment of cancer is based on the theory of its local origin, and implies the possibility of its complete removal. If this theory be true, failure to cure depends upon the essential inadequacy of the means used, or their untimely or inefficient employment. All remedial means are inadequate which have not the power to remove the diseased structures. The object of the treatment may be palliative or radical, the determination depending upon the location and extent of the disease and the general condition of the patient. Palliative measures are always available, while radical measures are not always safely applicable. Medical agents taken internally may be beneficial as palliatives, but are useless, so far as we know, in removing or modifying the progress of disease.

Conclusions: 1. Any operation for cancer which does not completely remove the disease will be followed by recurrence.

2. During life the limit of cancerous disease originating in any part of the uterus cannot be known; hence no operative procedure can guarantee complete removal.

3. In view of this fact, no operation is justifiable which greatly endangers life, provided other and safer methods are available.

4. Vaginal hysterectomy is more dangerous, in a certain sense, than the disease against which it is used; that is, a given number of patients afflicted with uterine cancer will live longer without than with the operation.

5. Other methods of treatment, attended by not more than one-sixth to one-fourth the mortality of vaginal hysterectomy, are equally efficient in ameliorating the symptoms and retarding the progress of the trouble, and they have been followed by as seemingly good results as regards recurrence. Hence they should be preferred.

6. Vaginal hysterectomy does not avert or lessen suffering; it destroys and does not save life. It is, therefore, not a useful but an injurious operation, and as such is unjustifiable.

Professor Graily Hewitt, of London, England, read on

THE RELATIONS BETWEEN CHANGES IN THE TISSUES AND CHANGES IN THE SHAPE OF THE UTERUS.

In order to determine more precisely the true relation existing between changes in the tissues of the uterus and changes in its form and shape, concerning which wide differences of opinion prevail, it is evident that the initial stage of these changes offer the widest field for inquiry.

In describing uterine tissue-changes, the term "chronic metritis" is generally employed. It is desired to call attention to a tissue-change sometimes observed on or soon after the arrival of puberty, especially in young women who have been inadequately nourished, consisting in undue softness of the uterine tissues, and associated with them in the beginning of uterine suffering. This undue softness is not "inflammatory" in its nature. It is associated with great flexibility of the uterus, and generally with marked flexion. The author first described it ten years ago, and has repeatedly remarked it since. It has recently been noticed by Dr. Charles D. Scudder, under the term "mollities uteri."

The recognition of the liability to occurrences of this initial change in the uterine tissues is to be regarded as very important, in the explanation of the origin and increase in degree of flexions of the uterus. In such cases the uterus being abnormally flexible, the flexion may be easily and gradually intensified by any ordinary exertion, but will be more likely to be much exaggerated and perpetuated by any severe and suddenly acting mechanical disturbance, The process by which the uterus becomes permanently flexed may thus be slow or rapid.

Hardening of the uterus occurs sooner or later. After hardening, the flexion is persistent.

In some few cases the flexion may be persistence of a congenital condition, or due to absence of developmental growth at the time of puberty without undue softness being present.

In multiparæ a somewhat analogous condition is present in what is known as "defective uterine involution," the uterine tissues being soft and wanting in resistance. As is generally admitted, slow flexions frequently originate at such times and under such circumstances.

The author contends that the interference with circulation present with uterine congestion is, in most cases, due to association of a weak blood-current and mechanical compression of uterine tissue, due to flexions present in such cases. The uterus being unduly soft, plastic, and mouldable, takes a flexed shape, which often becomes perpetuated by the hardening process described by Jacobi as the result of chronic metritis. One consequence of the latter is the presence of sclerosis of the uterine parenchyma. It is to be remarked that the incidents of some of the cases related by Jacobi favor the view that the flexion and displacement were operative in producing the menstrual sub-involution, rather than the cervical catarrh, which Jacobi assigns as the principal cause.

As regards endometritis, he considers the condition so described as more generally due to congestive hypertrophy of the uterine lining, and to retained irritating secretion, and that, excluding gonorrhœal and syphilitic cases, the endometritis is secondary rather than primary.

## Figures and Tables

**Figure f1:**
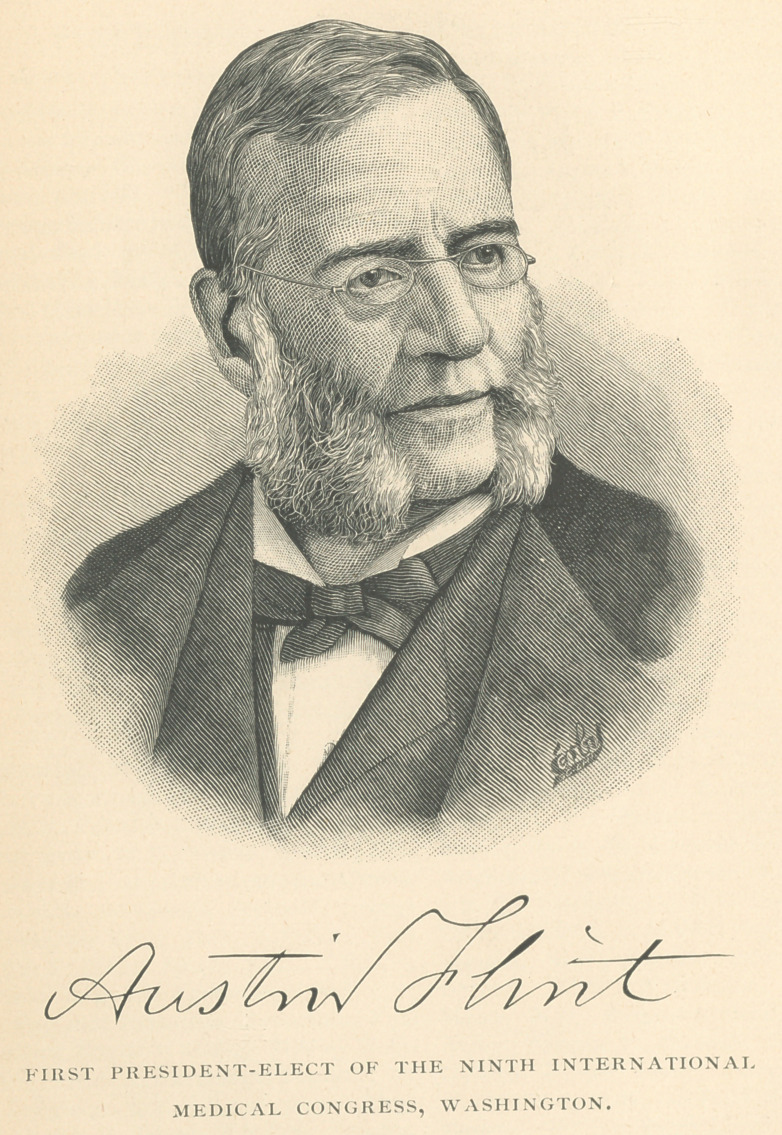


**Figure f2:**